# The Genetic Architecture of Behavior in the German Shepherd Dog

**DOI:** 10.1002/age.70221

**Published:** 2026-11-10

**Authors:** Voichita D. Marinescu, Kenth Svartberg, Marcin Kierczak, Katarina Tengvall, Matteo Bianchi, Jagoda Jabłońska, Per Arvelius, Sharadha Sakthikumar, Fabiana H. G. Farias, Åsa Karlsson, Mats E. Pettersson, Erik Wilsson, Erling Strandberg, Åke Hedhammar, Kerstin Lindblad‐Toh

**Affiliations:** ^1^ Department of Medical Biochemistry and Microbiology Uppsala University Uppsala Sweden; ^2^ Science for Life Laboratory Uppsala University Uppsala Sweden; ^3^ Department of Animal Biosciences Swedish University of Agricultural Sciences Uppsala Sweden; ^4^ ERASMUS PLUS Program Uppsala Sweden; ^5^ Swedish Armed Forces Dog Training Center Märsta Sweden; ^6^ Department of Clinical Sciences Swedish University of Agricultural Sciences Uppsala Sweden; ^7^ Broad Institute of MIT and Harvard Boston Massachusetts USA

**Keywords:** canine behavior, dog behavior, genome‐wide association studies, GWAS, neuroticism, personality trait, shyness‐boldness continuum

## Abstract

Studying canine behavioral genetics could provide important insights for dog breeding and for understanding both canine and human behavior. To identify genetic determinants of behavior in German Shepherd Dogs, we used a cohort of 373 dogs to search for genome‐wide associations with behavioral variables and personality traits measured by the Dog Mentality Assessment (DMA), a standardized dog behavior evaluation widely used in Sweden. Multidimensional scaling using genetic data revealed two clusters identified by merits as working and show sub‐populations differing in most of their DMA scores. These behavioral differences were strongly correlated with BOLDNESS—a higher‐order trait considered a measure of the shyness‐boldness personality continuum in dogs. A region with highest allele frequency differences between sub‐populations was found on CFA24: 23.5–25.5 Mb containing genes for coat color specification and previously reported candidate regions of selection. The strongest association signals were found for traits/behavioral variables correlating with BOLDNESS as, for example, on CFA1 within a region encompassing the *TCF4* gene for which, in humans, multiple associations with neuroticism (a reverse equivalent in humans of BOLDNESS in dogs) were reported. Signals of association were found in regions containing genes with important roles in the nervous system and associations with comparable personality traits, such as neuroticism, in humans. Our results indicate that within‐breed selection in GSDs has had an effect on the shyness‐boldness personality continuum, with working dogs scoring higher in all aspects of BOLDNESS—being more playful, prone to chase after toys, curious, fearless and socially interested in strangers compared with show dogs.

## 1 Introduction

The domestic dog ( *Canis familiaris* ) has been subjected to artificial selection from the time of its domestication to the present day. Early during domestication, selection probably favored traits crucial for human–dog social interaction. Later, during breed creation, selection emphasized traits beneficial for utilitarian tasks (such as hunting and herding) but also for esthetics (King et al. 2012; Lord et al. 2014; Wynne 2021). This shaped the typical appearance and behavior of a breed leading to the establishment of numerous breeds with striking morphological and behavioral differences (Mehrkam and Wynne 2014; Eken Asp et al. 2015). Domestication and breed creation have shaped the genome of modern breed dogs such that it contains a limited number of relatively short haplotypes embedded in longer blocks of high linkage disequilibrium (LD) (Lindblad‐Toh et al. 2005; Karlsson and Lindblad‐Toh 2008; Webster and Karlsson 2009), making it possible to identify relatively strong association signals in dog cohorts of much smaller sizes compared to humans.

The dog represents a powerful model for studying genetic diseases (Hayward et al. 2016) and also behavioral genetics (Morrill et al. 2022). Like humans, dogs display differences in temperament, social behavior, anxiety, aggression, and compulsive disorders (Hall and Wynne 2012). The Dog Mentality Assessment (DMA) was developed by the Swedish Working Dog Association as a tool for assisting in breeding working dogs (Svartberg 2002) and has been used to assess relevant aspects of dog behavior related to domestication and breed formation (Svartberg 2006; Hansen Wheat et al. 2019; Garamszegi et al. 2020). Over the years, DMA results for over a hundred thousand dogs from numerous breeds have been recorded providing a rich database of behavioral phenotypes for further studies (Pennisi 2007). The DMA is based on a standardized setup with 10 subtests where the dog is assessed by a trained rater using 33 protocol items—henceforth referred to as behavioral variables (Table S1). Factor analysis (Svartberg 2002), showed that the DMA behavioral variables could be aggregated into five or six (depending on breed group) first‐order factors—*PLAYFULNESS*, *DISTANCE PLAYFULNESS*, *CURIOSITY/FEARLESSNESS*, *CHASE‐PRONENESS*, *SOCIABILITY* and *AGGRESSIVENESS*. One second‐order factor labelled BOLDNESS, positively correlated to all first‐order factors except *AGGRESSIVENESS*, was also identified. Because the DMA factors have been found to be stable over repeated tests and correlated with everyday behavior as well as working dog test success, they have been considered as personality traits in dogs (Svartberg 2002, 2005; Svartberg et al. 2005). BOLDNESS was suggested as a representative in dogs of the shyness‐boldness continuum identified in humans and also in animal species from different taxa, including the wolf ( *Canis lupus* ) (Fox 1984; Kagan et al. 1988; Sloan Wilson et al. 1994). BOLDNESS has also been interpreted in terms of human personality traits and suggested to correspond to a dimension ranging from high Extraversion/low Neuroticism to low Extraversion/high Neuroticism (Svartberg 2002; Svartberg 2022).

Similarity between breed groups indicates that the structure of the DMA traits is general for dogs and has been maintained despite varied selection pressures during breed selection. Heritability estimates within breeds ((Strandberg et al. 2005) GSD, (Saetre et al. 2006) GSD and Rottweiler) reaching values above 0.2 for several traits and a maximum of 0.25 for BOLDNESS, suggest a genetic component for the traits, and indicate that considerable changes along behavioral dimensions could take place over relatively few generations. This is supported by results suggesting that breed differences in the DMA traits are caused by selection in the recent past and not by early selection during breed creation (Svartberg 2006). Moreover, recent within‐breed selection for different purposes led to establishing divergent types such as the *field* and *show/pet* types in golden and Labrador retrievers, which differ behaviorally in DMA traits related to BOLDNESS (Sundman et al. 2016).

The within‐breed selection for different purposes is evident in the German Shepherd Dog (GSD), a popular breed with a large range of uses including police and military work for defense and detection (Wilsson and Sinn 2012; Santariová et al. 2023). The breed originated at the end of the 19th century with dogs being selected from the outset for work, performance and temperament (Tenner 2017). However, an increased focus on appearance and fashion also led to breeding for participation and success in dog shows based on conformity to the morphological breed standard. As a result, an early split between *working‐* and *show*‐type dogs was already apparent in 1909 (Tenner 2017). These two types are well established in the modern breed, with *show*‐type dogs displaying morphological characteristics that, for example, affect movement and posture manifested in a characteristic slope and curvature of the top line, and angulations of the front and hind limbs (Humphries et al. 2020). There are indications that breeding for both working dog and show qualities in GSDs influences BOLDNESS, but in opposite directions (Meyer 2014).

In this study, we analyzed DMA traits/behavioral variables in a cohort of 373 genotyped GSDs. We performed exploratory factor analyses (EFA) on DMA data from a large sample of GSDs (*n* = 23,109) to confirm previously identified behavioral traits. We revealed strong population stratification within our cohort using multidimensional scaling (MDS) analyses of the genotype data, and we subsequently evaluated the within‐breed score differences in DMA traits/behavioral variables. We performed genome‐wide association studies (GWAS) for all DMA traits/behavioral variables to search for signals of associations common to all dogs and for those specific to the within‐breed sub‐populations, enabling us to evaluate also the consequences of divergent selection within breeding groups with a relatively homogeneous genetic background. We then placed the genes overlapping the associated regions in the perspective offered by the relevant biological knowledge available for them in mice and humans.

## 2 Materials and Methods

Complete method descriptions are provided in Supporting Information Methods. A brief summary is presented below.

We used a cohort of 373 German Shepherd Dogs (GSDs) assessed behaviorally using the DMA (Svartberg 2002) described in Table S1 and genotyped on the Illumina 170 K CanineHD Bead Chip (Illumina, San Diego, CA). The cohort had a female/male ratio *F/M =* 2 and the age in days at the time of the DMA had a median *age*_md_ = 496, a mean *age*_m_ = 538 and a standard deviation *age*_sd_ = 136.5.

All analyses were performed in the R statistical analyses suite (R Core Team 2019) version 3.6.1 using various packages as indicated. EFA was performed to identify first and second order factors using DMA data from 23,109 Swedish GSDs. For each dog, the value of the factor scores was computed as a weighted sum across all 33 behavioral variables and all loadings. From the resulting data, standardized trait/behavioral variables scores for the 373 GSDs genotyped using the Illumina 170 K CanineHD Bead Chip were extracted and used for further analyses. Throughout the paper, we used coordinates of the dog genome based on the CanFam3.1 reference genome assembly (Hoeppner et al. 2014). Prior to GWAS, carried out using the R package *GenABEL* (Aulchenko et al. 2007), we performed quality control and covariate analyses. To identify population stratification, we used multi‐dimensional scaling of the computed genomic‐kinship matrix between all dogs in our cohort (expressed as a distance matrix) followed by clustering analysis using the R package *mclust* (Scrucca et al. 2016). The resulting two clusters were identified as *working* and *show* sub‐populations by overlapping the certificates of merits of the dogs. To identify the genetic source of the population structure, at each position in the genome we evaluated the observed allele‐count differences between the two sub‐populations and tested their significance using Fisher's exact test for counts. The population assignment was used as a fixed effect in the GWAS for the entire cohort. GWAS was also performed for the two sub‐populations separately. The QC and covariate analyses were performed separately for all three GWAS sets.

Significance thresholds were determined empirically for each GWAS run by performing 1000 permutation tests on mixed model residuals and setting the significance threshold for the distribution of the resulting minimal empirical per‐marker *p* values to *α* = 5% using Student's *t*‐test. Significance was also checked against a threshold of –log_10_ *p* = 4.7 computed based on the average haploblock size in the dog genome (Lindblad‐Toh et al. 2005). SNPs scoring lower that the significance threshold but above –log_10_ *p* = 4 were reported as suggestive. We did not apply a correction for multiple testing for the number of GWAS we ran, since the DMA traits/behavioral variables analyzed are inter‐dependent (Figure S2A, Table S2A) and correlated (Figure S2B, Table S2B) and cannot be considered independent. Additional analyses of candidate regions were performed using the package *cgmisc* (Kierczak et al. 2015). For reproducibility, we make the *GenABEL* and *cgmisc* packages available as a Docker container that can be pulled directly from the GitHub repository (see https://github.com/cgmisc‐team/cgmisc). For genes of interest, information on loss‐of‐function phenotypes in mouse models was retrieved from the Mouse Genome Database (Blake et al. 2021) and that on human variant associations from the NHGRI‐EBI Catalog of human genome‐wide association studies (Sollis et al. 2023).

## 3 Results

Figure S1 summarizes the aims of our study and lists the sections presented below with their corresponding figures and tables.

### 3.1 EFA of DMA Scores for GSDs

EFA performed using all 33 DMA behavioral variables (Table S1) for 23,109 GSDs registered in the Swedish Kennel Club (SKK) retrieved six first‐order factors and one second‐order factor (Figure S2A, Table S2A, Supporting Information Methods), equivalent to what has been found previously (Svartberg and Forkman 2002). The first‐order factors were identified as *SOCIABILITY*, *PLAYFULNESS*, *CHASE‐PRONENESS*, *DISTANCE PLAYFULNESS*, *CURIOSITY/FEARLESSNESS*, and *AGGRESSIVENESS*, and the second‐order factor as BOLDNESS. With the exception of *AGGRESSIVENESS*, we found a high degree of correlation among the first‐order factors and between them and BOLDNESS (Figure S2B, Table S2B).

To test whether, in terms of DMA scores, our GWAS cohort of 373 genotyped GSDs is a representative subset of the larger population of 23,109 SKK‐registered GSDs, we compared the loadings from the EFA and the correlation coefficients between traits computed for both sets. The values were very similar (Table S2B, Figure S2), indicating that, in terms of behavior scores, our GWAS cohort is a representative subset of the larger SKK‐GSD population.

### 3.2 The Genotyped Cohort and Population Stratification

Since strong population structure was previously noted for a sub‐sample of our cohort (Tengvall et al. 2013), we used genotype data for all 373 GSDs to perform a MDS projection of genomic kinship distances between individuals. Subsequent model‐based clustering of this projection indicated two sub‐populations (Figure 1A). By comparing the proportion of different titles earned by the dogs in each cluster and their parents, we could ascribe them based on merits (Figure 1B) to a *working*‐type population (*n* = 222) and a *show*‐type population (*n* = 132), with a border sub‐population (*border*, *n* = 19), for which the cluster assignment was uncertain. A Wilcoxon rank sum test with continuity correction showed that proportions of *working*‐type titles (Swedish Working Champion—Bruksprovschampion, BCH; Police dog—Polishund, PH) were significantly higher in the *working*‐type population (*p*_BCH_ = 1.1 × 10^−7^ and *p*_PH_ = 6.3 × 10^−9^), while *show* titles (Finnish, Norwegian and Swedish Show Champion—Utställningschampion, UCH) were higher in the *show*‐type population (*p*_FI_UCH_ = 4.7 × 10^−3^; *p*_NO_UCH_ = 1.8 × 10^−4^ and *p*_SE_UCH_ = 1.4 × 10^−44^). The *working*‐type population shows lower mean degree of relatedness (Figure 1C, *d*_mean_ = 0.49, *p* < 2.2 × 10^−16^, Wilcoxon rank sum test with continuity correction) compared to the *show*‐type population (*d*_mean_ = 0.47).

**FIGURE 1:**
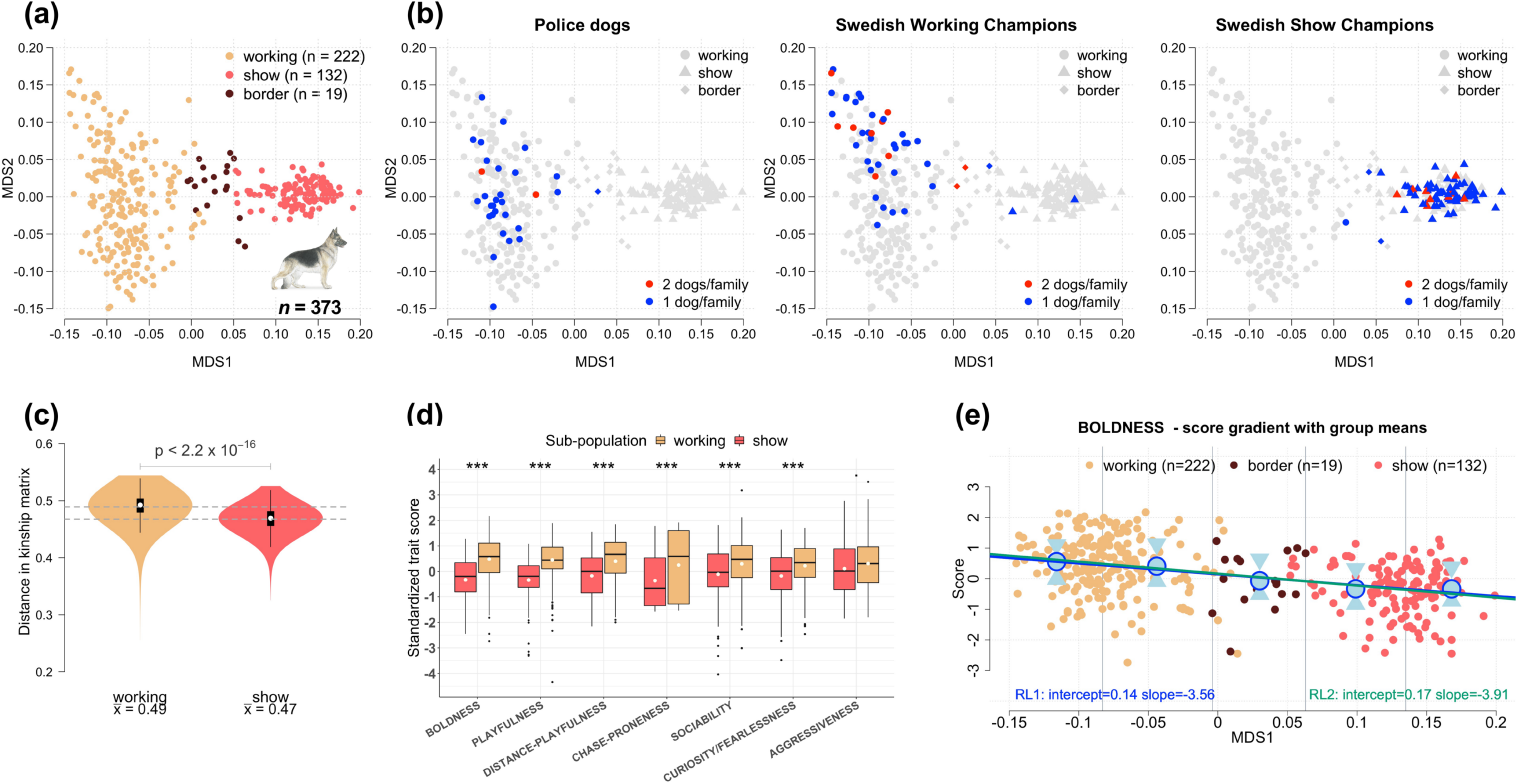
The GSD GWAS cohort. (a) Two‐dimensional MDS projection of genomic kinship matrix among the 373 genotyped GSDs showing a strong population stratification with two distinct clusters referred to as *working*‐type (beige dots, each dot corresponds to an individual) and *show*‐type (red dots). Black dots represent the 5% most uncertainly classified individuals treated as a separate *border* sub‐population. (b) *Working* and *show* sub‐populations were assigned by overlapping titles and merits earned by individual dogs (and their parents, red symbols for 2 dogs/family) on the MDS projection of the genomic kinship matrix. All Police dogs in the cohort (Polishund, PH) belonged to the *working*‐type sub‐population as did all Swedish Armed Forces dogs and nearly all Swedish working dog champions (Svensk Brukshundchampion, SE BCH), while all dogs with Swedish show champion titles (Svensk Utställningschampion, SE UCH) belong to the *show*‐type population. (c) Differences in the average degree of relatedness between the *work* and the *show* sub‐populations. (d) Comparative boxplot distributions of scores in the *show* and the *working* sub‐populations for the second‐order trait BOLDNESS and the first‐order traits sorted by the magnitude of score difference (*Z*). Mean values are represented with white dots and median values with black bars. Significance stars (‘***’ *p* ≤ 0.001, ‘**’ *p* ≤ 0.01, ‘*’ *p* ≤ 0.05, ‘.’ *p* ≤ 0.1) refer to the results of a Mann–Whitney U test of difference in means between the score values for the trait in the *working* versus the *show* sub‐population (Table S3). (e) Score gradients along the first MDS dimension (MDS1) in the three sub‐populations exemplified for BOLDNESS. Each dot corresponds to the BOLDNESS score of one individual. Perpendicular bars represent division points within sub‐populations based on low versus high MDS1 values. Blue filled circles represent score group means and triangles mark the positions of the first and third quartile within each distribution. Blue (RL1) and green (RL2) lines represent regression lines fit through the resulting group mean points obtained by dividing the cohort in five groups (see Supporting Information Methods), showing very similar values of the intercept and slope.

The distribution of standardized scores for BOLDNESS and the six first‐order factors in the *show* and the *working* sub‐populations (Figure 1D) indicates that *working*‐type dogs have statistically significantly higher mean and median score values for all traits except *AGGRESSIVENESS* compared with *show*‐type dogs. To evaluate the difference in score for each trait/behavioral variable between the two sub‐populations, we employed a Mann–Whitney U test and used the resulting adjusted *Z* score as a measure of magnitude (Table S3). The largest score differences were obtained for *PLAYFULNESS* (*Z* = 8.91) and BOLDNESS (*Z* = 7.86). Given the importance of BOLDNESS as a measure of the shyness‐boldness personality continuum in dogs, we also computed for each of the six traits and 33 behavioral variables the relationship to BOLDNESS (Boldness relationship, BR) as the Spearman rank correlation coefficient *r*_*s*_ between the scores for the trait/behavioral variable and the scores for BOLDNESS across the 373 GSDs in our study (Table S3). We found that in our cohort, the trait/behavioral variable score differences between the *working* and the *show* sub‐populations (*Z*) were highly correlated with BR values (*r* ^*2*^ = 0.92, *p* < 2.2e‐16) as shown in Figure 2. Traits/behavioral variables most positively correlated with BOLDNESS include *DISTANCE PLAYFULNESS* (*Z* = 6.02, BR = 0.86) and *PLAYFULNESS* (*Z* = 8.91, BR = 0.73), while traits most negatively correlated with BOLDNESS include *6d. Remaining avoidance behavior* (*Z* = −3.75, BR = −0.4) and *7a. Startle reaction* (*Z* = −0.34, BR = −0.41).

**FIGURE 2:**
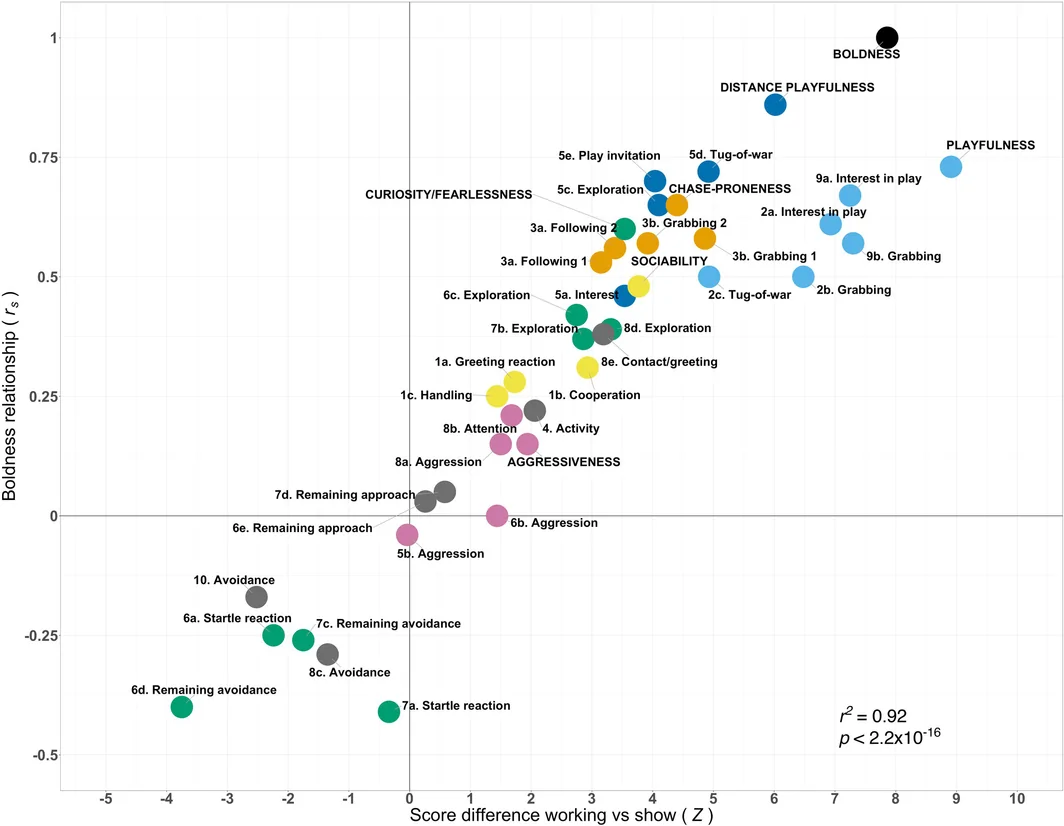
The correlation between the degree of score difference and the Boldness relationship for all traits/behavioral variables. The degree of score difference between the *working* and the *show* sub‐populations (*Z*) for the 40 traits/behavioral variables shows high correlation (*r* ^2^ = 0.92, *p* < 2.2 × 10^−16^) with the Boldness relationship (*r*_s_). Behavioral variables are color coded according to the traits on which they load as represented in Figure S2; those not loading on any of the traits are colored in gray.

When using the values of the first MDS dimension (MDS1) across our cohort (a reflection of the genetic differences between sub‐populations) to divide the cohort into five groups (*working*‐type dogs with low and high MDS1 values, *border*‐type dogs, and *show*‐type dogs with low and high MDS1 values) we noticed that, for each trait/behavioral variable, the mean score values across the five groups define a gradient with a slope directly proportional to the amplitude of score difference between the two sub‐populations. Figure 1E exemplifies the score gradient for BOLDNESS.

In order to identify genetic loci potentially contributing to the observed population structure we performed a genome‐wide scan for allele frequency differences between the *working* and the *show* sub‐populations. A ~2 Mb long region between CFA24: 23.5–25.5 Mb CanFam3.1 (Hoeppner et al. 2014) showed the strongest difference in allele frequencies. The top haplotype (Figure 3A, Table S4) reaches nearly complete fixation in the *show* sub‐population (Figure 3B inset—maximum fixation index F_*ST*_ = 0.62 for three SNPs, Figure 3D). The top‐divergent SNP showing the highest allele frequency difference (BICF2P1122354, −log_10_ *p* = 40.8, in magenta in Figure 3F), is located upstream of the *TRPC4AP* (*transient receptor potential cation channel subfamily C member 4 associated protein*) gene. Its C allele is completely fixed in the wolf population examined by (Axelsson et al. 2013) and segregates in the *working* sub‐population (*f*_C_ = 0.64), while the alternative allele A is nearly fixed (*f*_A_ = 0.99) in the *show* sub‐population.

**FIGURE 3:**
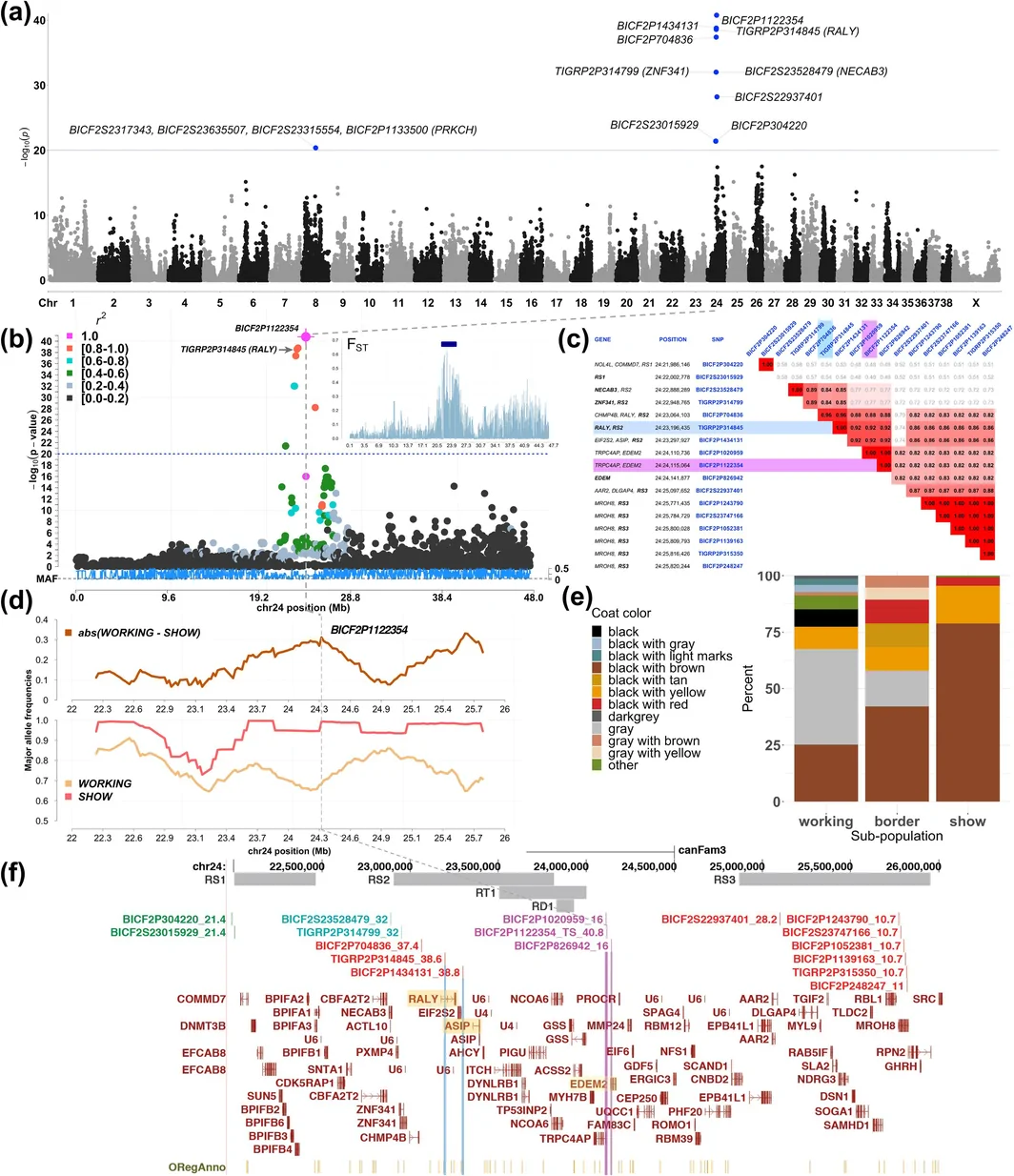
The genomic region with greatest differences by allele frequency between the *working* and the *show* sub‐populations. (a) Manhattan plot of −log_10_ *p* values obtained using a Fisher's exact test for counts comparing the theoretical and observed reference allele counts between the *working* and the *show* sub‐populations at each position in the genome. The region showing the greatest differences is located on CFA24. (b) LD Manhattan plot of −log_10_ *p* values within the CFA24: 23–26 Mb genomic fragment showing the region with greatest differences in allele frequency containing the top divergent SNP (BICF2P1122354) in perfect (*r* ^2^ = 1) or high LD (*r* ^2^ ≥ 0.8) with 17 other SNPs. Similarly, an *F*_ST_ scan (inset) in 1 Mb windows across CFA24 shows a corresponding peak between positions CFA24: 22–26 Mb (blue rectangle). (c) LD (*r* ^*2*^) matrix for the 17 SNPs in the CFA24: 22–26 Mb region that are either in high (*r* ^*2*^ ≥ 0.8) LD with the top‐divergent SNP (highlighted in magenta) or have −log_10_ *p* > 20. For each SNP, genes located upstream or downstream are displayed in italics and genes in which a SNP falls in bold. Three regions of selection on CFA24 previously reported in GSDs (Friedrich et al. 2019) are indicated by *RS1*, *RS2* and *RS3*. The SNP in *RALY* is highlighted in blue. (d) Absolute differences in major allele frequencies (top panel, brown) and major allele frequencies per sub‐population (bottom panel, beige—*working* and red—*show*) smoothed using a sliding overlapping window of 21 markers with a 1 marker step. A ~2 Mb region of near complete fixation between positions CFA24: 23.5–25.5 Mb is apparent in the *show* sub‐population. (e) Coat color distribution across the three sub‐populations. Coat colors present at < 1% within each sub‐population were omitted. (f) Genes located within the CFA24: 22–26 Mb genomic region. To highlight the strength of the LD with respect to the top‐divergent SNP, IDs are displayed using the color convention for *r* ^*2*^ of Panel B with the ‐log_10_ *p* values appended as a suffix. Previously documented regions of selections for dog domestication (RD), tameness in foxes (RT) and recent selection between UK and Swedish GSDs (RS) are represented with gray bars (see Supporting Information Results SR1a for details).

Within a larger ~4 Mb long region between positions CFA24: 22–26 Mb 17 SNPs were either in high LD (*r* ^2^ ≥ 0.8, computed across the entire cohort) with the top‐divergent SNP and/or reached in our allele frequency difference scan values of −log_10_ *p* > 20 (Figure 3B,C). Three SNPs in high LD with the top‐divergent SNP are located in, or close to, genes with known roles in coat color specification in dogs and mammals. From left to right in Figure 3F these are: BICF2P704836 (*r* ^2^ = 0.88, rank 4 by *p* in the top divergent SNPs, Table S4) ~110 kb upstream of the *RALY Heterogeneous Nuclear Ribonucleoprotein* (*RALY*), TIGRP2P314845 (*r* ^2^ = 0.92, rank 3) in the first intron of *RALY*, and BICF2P1434131 (*r* ^2^ = 0.88, rank 2) ~9 kb upstream of the *Eukaryotic Translation Initiation Factor 2 Subunit Beta* (*EIF2S2*) gene and ~98 kb upstream of the *Agouti signaling protein* (*ASIP*) gene. The proximity of positions with very high differences in allele frequency between the two sub‐populations to the *ASIP* locus involved in coat color specification prompted us to assess the distribution of coat colors within sub‐populations (Figure 3E). *Show* dogs in our cohort display three predominant coat colors—*black with brown* (~79%), *black with yellow* (~17%) and *black with red* (~4%), while *working* dogs show a greater variety of coat colors with the most common one being *gray* (~42%).

The CFA24: 22–26 Mb region also contains several previously reported (Friedrich et al. 2020; Freedman et al. 2016; Kukekova et al. 2018) candidate regions of selection in GSDs, dog domestication and tameness in foxes (represented as gray bars in Figure 3F; labelled for simplicity with *RS, RD* and *RT* in the order given in Table S5 that presents their coordinates and reporting studies). The mapping of the most divergent SNPs on these regions (Figure 3F) is detailed in Supporting Information Results SR1a.

### 3.3 GWAS Results

Differences at the genetic and behavioral score levels observed between the *working* and the *show* sub‐populations prompted us to search not only for association signals common to all dogs in our cohort but also for those specific to each sub‐population. Therefore, for each of the 40 traits/behavioral variables analyzed (33 behavioral variables, 6 traits and one second‐order trait BOLDNESS), we performed GWAS in three separate runs: for the entire cohort and for the *working* and the *show* sub‐populations separately. Genome‐wide significant associations (*p* lower than a threshold determined empirically based on 1000 permutations) were reported if found in any of the three runs. For 28 of the 40 traits/behavioral variables, at least one significant association was found in at least one of the three GWAS runs (Figure S3A). When evaluating the GWAS results, we also considered suggestive SNPs that scored below the threshold of significance in their respective runs but had a ‐log_10_ *p* value > 4. In many cases, the same SNPs were found as significant/suggestive in multiple GWAS runs for the same or a different trait/behavioral variable. The total number of unique significant/suggestive SNPs found was equal to 737 (Table S6), out of which 100 SNPs were retrieved as significant in at least one GWAS run for any of the traits/behavioral variables. The significant SNPs are distributed across 22 autosomes and, in many cases, concentrated in distinct chromosomal regions (Figure S3B), a pattern more apparent when the suggestive SNPs are also considered. Taken together, the distribution of significant and suggestive SNPs is spread across the entire set of 38 autosomes (Figure S3C).

Several SNPs were retrieved as significant/suggestive for three or more related traits/behavioral variables including *SOCIABILITY*, *PLAYFULNESS*, *CHASE‐PRONENESS*, *DISTANCE PLAYFULNESS*, BOLDNESS, and some of their components (Supporting Information Results SR2, Tables S7A and S7B).

Several significant associations were identified for traits/behavioral variables that were related to BOLDNESS to a high degree and showed large differences in scores between the two sub‐populations (i.e., have high absolute values for BR and *Z*, Table S3, Figure 2). At the boldness end of the shyness‐boldness axis, traits/behavioral variables most positively related to BOLDNESS for which significant associations were identified belong to: (1) BOLDNESS, *DISTANCE PLAYFULNESS* and *5d. Tug‐of‐war*, (2) *PLAYFULNESS* and its components, (3) *3b1. Grabbing* (a component of *CHASE‐PRONENESS*) and (4) *SOCIABILITY* and its components. At the shyness end of the shyness‐boldness axis, significant associations were found for *6d. Remaining avoidance* *behavior* (a component of *CURIOSITY* */* *FEARLESSNESS*), showing the second highest negative correlation with BOLDNESS and the highest negative difference in scores (*Z* = −3.75) between the *working* and the *show* sub‐populations. Association signals for all five groups are presented in Table S8.

#### 3.3.1 BOLDNESS, *DISTANCE PLAYFULNESS* and *5d. Tug‐of‐War*

In the *show* sub‐population, signals of association for *5d. Tug‐of‐war*, *DISTANCE PLAYFULNESS* and BOLDNESS fall within the same region on CFA1: 19.5–20.9 Mb (Figure 4A) and consist of a total of 14 significant and/or suggestive SNPs (Figure 5A, Tables S6 and S8). The top SNPs are different between *5d. Tug‐of‐war (*BICF2S23248133, CFA1: 20,228,210, −log_10_ *p* = 5.53) and *DISTANCE PLAYFULNESS* and BOLDNESS (BICF2P1398938, CFA1: 20,032,821, −log_10_ *p* = 5.14 and −log_10_ *p* = 5.46, respectively) and their LD patterns are slightly different (Figure 4B). The CFA1: 19.5–20.9 Mb region harbors the genes *Transcription Factor 4* (*TCF4*), *Coiled‐Coil Domain Containing 68* (*CCDC68*) and *RAB27B, Member RAS Oncogene Family* (*RAB27B*). The top SNPs for *DISTANCE PLAYFULNESS* and BOLDNESS are located ~28 kb upstream of *TCF4* and the top SNP for *5d* is intronic to the gene (Figure 5A, Table S8).

**FIGURE 4:**
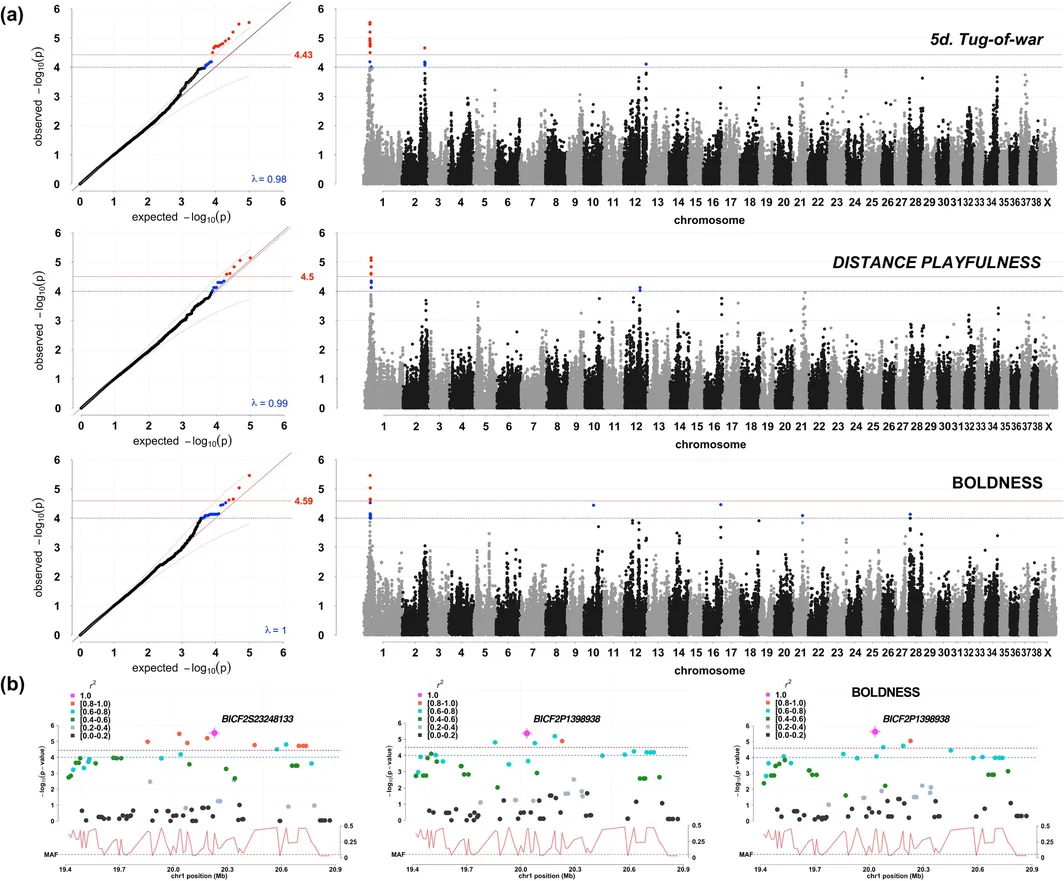
GWAS results in the *show* sub‐population for *5d*. *Tug‐of‐war, DISTANCE PLAYFULNESS* and BOLDNESS. (a) Q‐Q and Manhattan plots for *5d*, *DISTANCE PLAYFULNESS*, and BOLDNESS showing an overlapping association signal on CFA1. *5d* shows an additional signal of association on CFA2. (b) LD Manhattan plots for the three traits/behavioral variables within the CFA1: 19.4–20.9 Mb region. LD values were calculated with respect to the corresponding top SNPs represented in magenta and marked with a cross (BICF2S23248133 for *5d*, and BICF2P1398938 for *DISTANCE PLAYFULNESS* and BOLDNESS).

**FIGURE 5:**
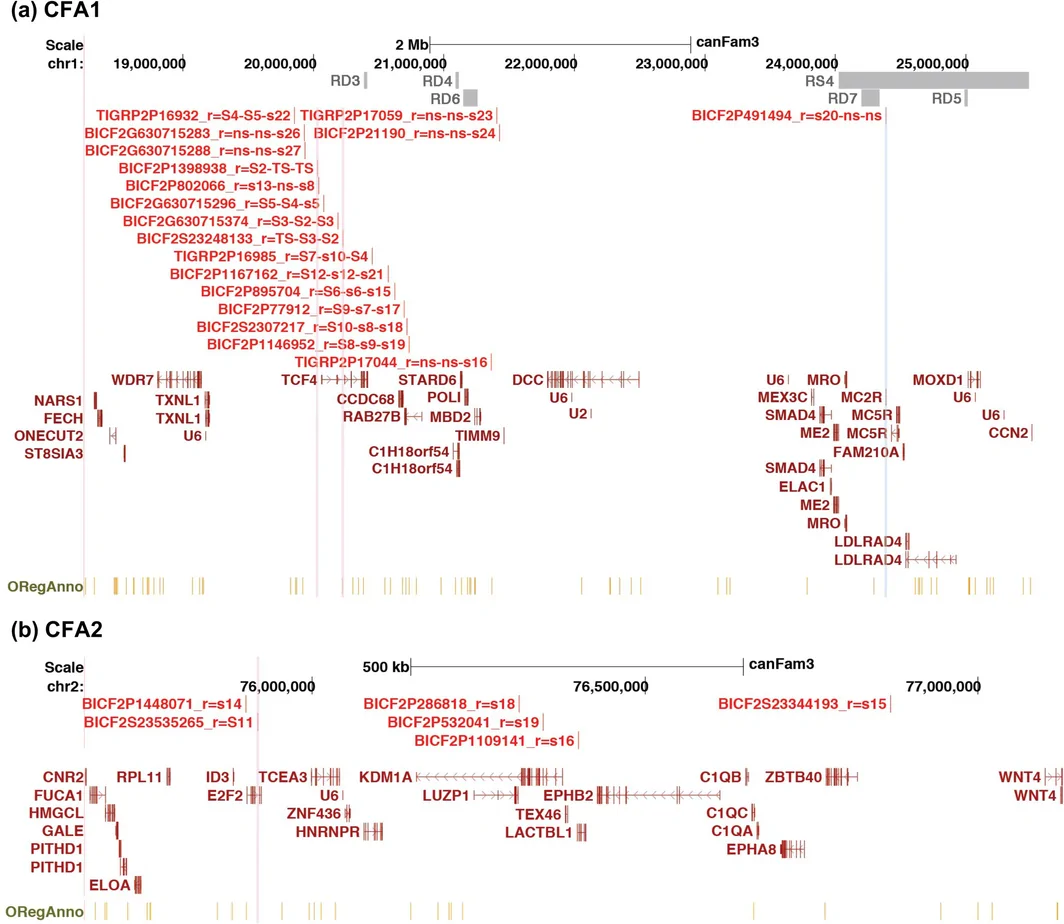
The genomic region for the association signals on CFA1 and CFA2 obtained for *5d. Tug‐of‐war*, *DISTANCE PLAYFULNESS* and BOLDNESS in the *show* sub‐population. (a) The IDs of the SNPs within the association signal on CFA1 identified for the three traits/behavioral variables are annotated to indicate their significance and rank (by *p*, within the significant or suggestive SNPs, as appropriate) in the respective GWAS listed in the order *5d*‐*DISTANCE PLAYFULNESS*‐BOLDNESS. *TS* stands for top SNP, *S* for significant, *s* for suggestive and *ns* for not significant. In a hypothetical example, _r = TS‐S3‐s2 would signify that the SNP is the top SNP for *5d*, the third by *p* among the significant ones for *DISTANCE PLAYFULNESS* and the second by *p* among the suggestive ones for BOLDNESS. The positions of the top SNPs are highlighted with magenta lines and the position of the suggestive SNP obtained for *5d* alone with a blue line. (b) Significant and suggestive SNPs on CFA2 identified for *5d* only are annotated to indicate their GWAS rank (*S* stands for significant, *s* for suggestive). The position of the significant SNP on CFA2 is highlighted with a magenta line.

In the *show* sub‐population, *5d* alone shows two additional signals on CFA1 and CFA2. On CFA1, a suggestive SNP (BICF2P491494, −log_10_ *p* = 4.01) is located ~1.2 kb upstream of the *melanocortin 2 receptor* (*MC2R*) gene (blue highlight in Figure 5A). On CFA2 (Figure 5B), the association region consists of one significant SNP (BICF2S23535265, −log_10_ *p* = 4.66 above the permutation threshold but below the 4.7 threshold based on average haploblock size) that is intronic to the *E2F Transcription Factor 2* (*E2F2*) gene and four suggestive SNPs. Genotype–phenotype relationships for the associated SNPs of interest on CFA1 and CFA2 are presented in Supporting Information Results SR3 and Figure S4.

The CFA1: 20–26 Mb region (from the *TCF4* to the *CCN2* loci) contains several previously reported regions of selection for dog domestication and regions of recent selection in GSDs. These are described in Supporting Information Results SR1b, are represented as gray bars in Figure 5A and are labelled for simplicity with *RD* and *RS*, respectively, in the order given in Table S5 that presents their coordinates and reporting studies.

Results for the additional traits/behavioral variables most positively related to BOLDNESS for which significant and suggestive associations were also found, namely, *PLAYFULNESS* and contributing variables, *3b1. Grabbing* (component of *CHASE‐PRONENESS*), *SOCIABILITY* and its components, are presented in Supporting Information Results SR4.

#### 3.3.2 *6d. Remaining Avoidance Behavior* Contributing to *CURIOSITY/FEARLESSNESS*

For *6d*, at the shyness end of the shyness‐boldness axis, a significant association was obtained in the *working* sub‐population on CFA15 (Figure 6A). Five top SNPs (Tables S6 and S8), located between CFA15: 10,767,562–10,888,783 are in perfect LD with each other (*r* ^*2*^ = 1) and have identical significance levels (−log_10_ *p* = 6.1). Together with 52 other SNPs in high LD (*r* ^*2*^ ≥ 0.8) with them (including two significant and 50 suggestive), the top SNPs define a region of ~4 Mb between CFA15: 8.5–12.5 Mb (Figure 6B) that contains genes such as *ELAVL4, OSBPL9, SHISAL2A, FAF1, AGBL4, NRDC, TUT4, ORC1, SCOA7, ZYG11B* and *ZFYVE9* (Figure 6D). One of the top SNPs, BICF2S23130195, is intronic to the *ELAV Like RNA Binding Protein 4 (ELAVL4*) gene, while the remaining four top SNPs fall within ~121 kb in the 3′ region of the gene (Figure 6D). Two additional significant SNPs located upstream of the top five SNPs, are intronic to the *Shisa Like 2A* (*SHISAL2A*) and the *oxysterol binding protein like 9* (*OSBPL9*) genes, respectively. The minor allele G of the top SNP BICF2S23512032 is present in one or two copies in 22 *working* dogs that have higher phenotypic scores (corresponding to decreased fear responses due the negative sign of the *6d* loading on *CURIOSITY/FEARLESSNESS*). The same effect is seen for the minor allele of the other four top SNPs (Figure 6C). The values of the major allele frequencies for the SNPs within the CFA15: 8.5–12.5 Mb genomic fragment (Figure 6E) indicate that the region downstream of the top SNPs (that includes multiple suggestive SNPs in LD with the top SNPs) shows near fixation in the *working* sub‐population but not in the *show* sub‐population.

**FIGURE 6:**
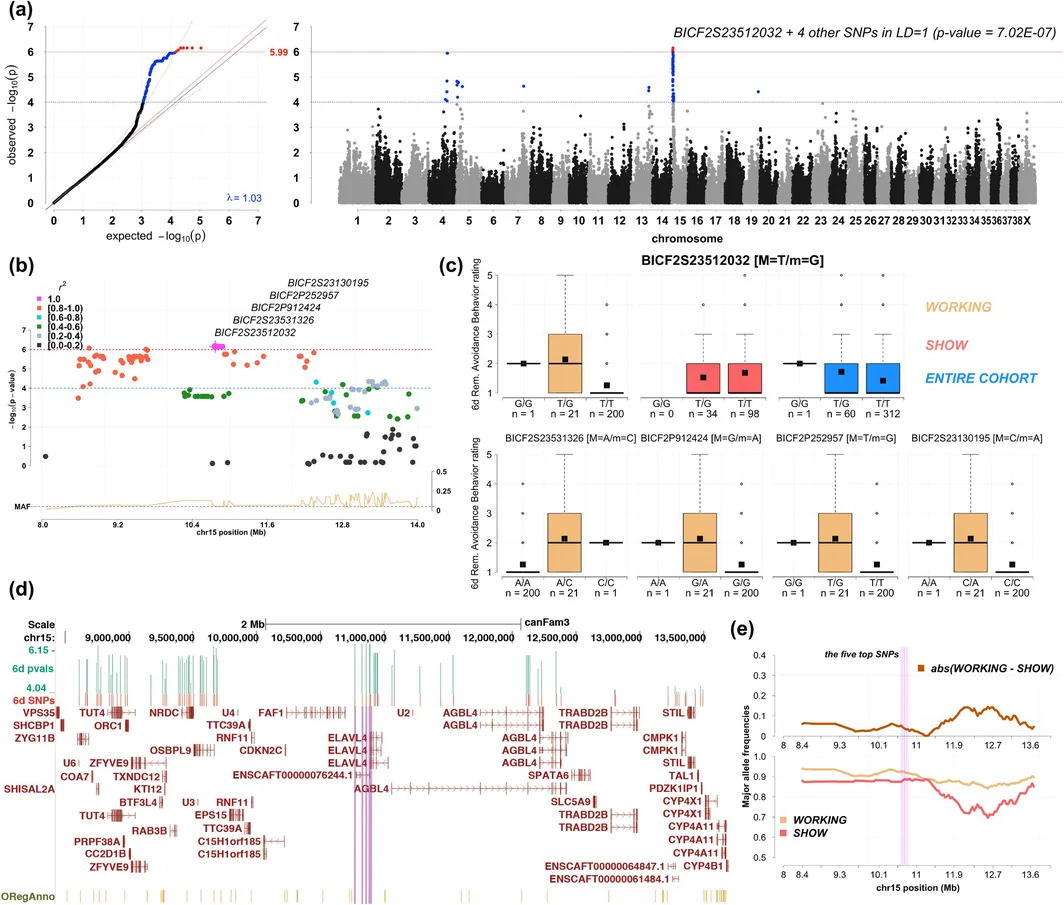
Significant association signals in the *working* sub‐population for *6d. Remaining avoidance behavior* contributing to *CURIOSITY/FEARLESSNESS*. (a) Q‐Q and Manhattan plots for *6d* in the *working* sub‐population showing a significant signal of association on CFA15. (b) LD‐Manhattan plot showing the SNPs in LD with the first (by position) of the five top SNPs (BICF2S23512032, represented in magenta and marked with a cross). All five top SNPs are in perfect LD (*r* ^*2*^ = 1) with each other. (c) Genotype–phenotype plots using the original *6d* DMA ratings on a 1–5 scale for the allele combinations of (1) the top SNP BICF2S23512032 in the *working*/*show* sub‐populations and the entire cohort (top row) and (2) the other four top SNPs BICF2S23531326, BICF2P912424, BICF2P252957 and BICF2S23130195 in the *working* sub‐population. M stands for the major allele, m for the minor allele. In the boxplot distributions, mean and median values are indicated by square black dots and black lines, respectively. (d) The region of association presented alongside two UCSC custom tracks displaying the ‐log_10_ *p* values (first track, green) for the identified significant and suggestive SNPs (second track, red). (e) Major allele frequency plots for the SNPs within CFA15: 8–14 Mb in the *working* (beige line) and the *show* (red line) sub‐populations and the absolute difference between them (brown line). The positions of the five top SNPs are indicated with magenta lines.

## 4 Discussion

Our study used genotype information and behavioral DMA data for a well‐defined population of 373 Swedish GSDs. MDS projection of genetic distances between individuals followed by model‐based clustering revealed the presence of two clusters (Figure 1A) identified by mapping certificates of merits as *working* and *show* sub‐populations (Figure 1B).

Morphological differences between the *working* and *show* types of GSDs have been previously documented (Humphries et al. 2020). Our analyses show that they also display behavioral differences related to the shyness‐boldness axis as measured by the higher‐order trait BOLDNESS. The *working* dogs are, as a group, bolder in all aspects (i.e., they are more playful, curious, prone to chase after a fleeing toy, fearless, and easier to socially engage with a stranger compared with *show* dogs). Differences in DMA behavior between types within a breed, for example, the *field* and *show*/*pet* types of the golden retriever and Labrador retriever breeds, have previously been reported (Sundman et al. 2016), and, as in our study, they pertain to aspects of BOLDNESS. The association between high BOLDNESS and *working* dogs is consistent with previous DMA studies on *working* dog trial performance (Svartberg 2002) and breed differences (Svartberg 2006). It is also in line with results from the Swedish Armed Forces breeding program, where the strongest predictors of success in the training program for *working* GSD were “Confidence”—related to curiosity and low fear, and “Physical engagement”—related to high interest in playing (Wilsson and Sinn 2012). Meyer (2014) reported results from a questionnaire survey where Swedish breeders were anonymously asked about their breeding goals. Matching of questionnaire results from 163 breeders of GSDs with DMA data from 4131 bred dogs indicated a clear relationship between the breeding goals of the breeders and BOLDNESS. Breeding for show qualities were associated with generally low BOLDNESS, while breeding for trial and training qualities, including working dog qualities, were coupled with high BOLDNESS, especially *PLAYFULNESS* and *CHASE‐PRONENESS*. Together, these findings suggest that differentiated breeding within the GSD breed has had an effect on the shyness‐boldness axis.

We retrieved significant association signals for traits/behavioral variables most positively and most negatively related to BOLDNESS. At the boldness end of the shyness‐boldness axis (Figure 2) we obtained genome‐wide significant signals for BOLDNESS, *DISTANCE PLAYFULNESS* and *5d*. *Tug‐of‐war*, but also for *PLAYFULNESS* and its contributing behavioral variables, *3b*. *Grabbing 1* (contributing to *CHASE‐PRONENESS*) and *SOCIABILITY* and its components. At the shyness end, we obtained significant association signals for *6d*. *Remaining avoidance* *behavior* (contributing to *CURIOSITY* */FEARLESSNESS*), a measure of how long the dog needs to recover from a fearful experience. The regions and genes corresponding to these signals of association (Table S8) may have played a role in generating the behavioral differences between the *working* and the *show* GSD subtypes.

The significant region of association on CFA1 overlapping the *TCF4* gene was identified in the *show* sub‐population for all three traits/behavioral variables BOLDNESS, *DISTANCE PLAYFULNESS*, and *5d. Tug‐of‐war* (Figure 4). But for *5d* alone, we also retrieved a suggestive SNP (Figure 5A) located 1.2 kb upstream of the gene encoding MC2R, formerly known as the Adrenocorticotropic Hormone Receptor—ACTHR. Scores for *DISTANCE PLAYFULNESS* and BOLDNESS are computed using loadings from multiple other traits/behavioral variables than *5d*, and this could obscure a weak signal associated specifically with *5d*. MC2R is a G protein‐coupled receptor expressed in the adrenal cortex where it binds the adrenocorticotropic hormone (ACTH) in the first step of activating the signaling pathway leading to the synthesis of glucocorticoids (Switonski et al. 2013), stress hormones represented in humans and dogs by cortisol. Tonoike et al. (2022) found associations with three polymorphisms in *MC2R* supporting the connection between cortisol and the ability of the dog to make behavioral adjustments based on interaction with humans.

The genomic regions of interest on CFA1 and CFA24 harbor multiple, previously reported, candidate regions for selection in GSDs, dog domestication, and tameness in foxes (Friedrich et al. 2020; Freedman et al. 2016; Kukekova et al. 2018) (Table S5). To interpret the significance of their presence within our regions of interest we could consider that the basic behavioral differences due to domestication are decreased fear and increased sociability, including decreased aggressive behavior towards humans (Kukekova et al. 2022). Within the DMA framework, this strongly resembles *SOCIABILITY* that could be defined as a dimension ranging from rejection of contact (that could include biting and withdrawal) to excessive greeting. But since sociable dogs are often also explorative, fearless and playful (i.e., generally bold) it could be argued that the domestication effects on behavior resemble a general increase in BOLDNESS, as defined by the DMA. This is supported by the finding that selection against aggression in domesticated animals often leads to an increase in exploratory behavior (Kunzl and Sachser 1999; Kunzl et al. 2003).

Many genes located within the associated regions have important roles in neurodevelopment, display behavioral and neurological phenotypes in loss‐of‐function mouse models and are associated in humans with psychological/psychiatric traits and/or neuropsychiatric disorders (Table S8). Three examples of such genes are *UNC5D, ELAVL4* and *TCF4*. *UNC5D* (CFA16, Figure S5B, association for *2b*. *Grabbing*), encodes a member of a family of receptors for netrins—secreted molecules with bifunctional role in attracting or repelling neuronal growth cones during the development of the nervous system (Keleman and Dickson 2001). *ELAVL4* (CFA15, Figure 6D, association for *6d*. *Remaining avoidance behavior*), encodes a RNA‐binding protein that controls the metabolism, stability, degradation and translation of many mRNAs with important roles in the development, plasticity and function of the nervous system (Silvestri et al. 2022). In humans, variation in *ELAVL4* has been associated with several traits including schizophrenia, unipolar depression, and attention deficit hyperactivity disorder (Sollis et al. 2023). *TCF4* (CFA1, Figure 5A, association for BOLDNESS, *DISTANCE PLAYFULNESS* and *5d. Tug‐of‐war*), encodes a broadly expressed basic helix–loop–helix transcription factor that is an important regulator of the epithelial–mesenchymal transition and of neurodevelopment (Forrest et al. 2014). In humans, mutations in *TCF4* cause the Pitt‐Hopkins syndrome characterized by developmental delays, moderate to severe intellectual disability and behavioral differences among other defects (Rappaport et al. 2017), while variation in the gene is associated with neuroticism, schizophrenia, bipolar disorder, post‐traumatic stress disorder and major depression disorder (Teixeira et al. 2021). Loss‐of‐function *TCF4* mouse models show abnormal associative learning, impaired contextual conditioning behavior and spatial learning, abnormal habituation to a new environment, decreased anxiety‐related response, and social withdrawal among other defects (Table S8, Blake et al. 2021).

Given the large number of loci and candidate genes identified and the expected polygenic architecture of behavioral traits we performed enrichment analyses to identify patterns related to over‐representation of biological pathways or expression in particular tissues. KEGG pathway enrichment analysis (Table S9) of the genes overlapping or located immediately upstream and downstream of the significant and suggestive markers in Table S8, showed statistically significant enrichment for pathways such as *Cushing's syndrome*, *glutathione metabolism* and *insulin resistance* all of which have been associated with changes in behavior in dogs or other models. Cushing's syndrome (hyperadrenocorticism) caused by prolonged exposure to high levels of cortisol was associated in dogs with behavioral changes such as anxiety, aggression, and fear‐related behaviors (Mârza et al. 2024). Glutathione metabolism is directly linked with glutamine metabolism as glutamine is converted into glutamate, a direct precursor for glutathione synthesis. In humans, changes in glutamine homeostasis lead to neuronal glutamine deficiencies in the prefrontal cortex that are associated with depression, chronic stress, and cognitive impairment (Baek et al. 2024) while increased glutamine concentrations in the left dorsolateral prefrontal cortex are associated with neuroticism and trait anxiety (Hasler et al. 2019). Moreover, in dogs, plasma levels of glutamine and the metabolic intermediate γ‐glutamyl glutamine (γ‐Glu Gln) were found to be elevated in fearful dogs across breeds (Puurunen et al. 2018). This is consistent with the fact that the candidate genes included in the glutathione metabolism pathway *GPX7* and *TXNDC12* corresponded to suggestive markers obtained for *6d. Remaining avoidance behavior*. In mice, brain insulin resistance affects dopamine turnover and induces anxiety and depressive‐like behaviors (Kleinridders et al. 2015). Tissue enrichment analyses showed that 44% of the candidate genes are highly expressed in the prefrontal cortex and/or the spinal cord with a third of them (33%) being highly expressed in the prefrontal cortex (Supporting Information Results SR6). In humans, the prefrontal cortex represents a critical “cognitive hub” also involved in emotional and social behavior, attention, working memory and inhibitory control among other processes (Chini and Hanganu‐Opatz 2021). Even though the DMA traits may be more related to motivation and emotion rather than to cognition, the ability to perform well in the DMA and well as a working dog is strongly dependent on higher cognitive functions (Bray et al. 2021; Hare and Ferrans 2021).

In terms of human personality traits, BOLDNESS in dogs has been interpreted as a dimension ranging from high Extraversion/low Neuroticism to low Extraversion/high Neuroticism (Svartberg 2002; Svartberg 2021, 2022). Several genes within the CFA1: 20.0–24.5 Mb region containing the signals of association for BOLDNESS, *DISTANCE PLAYFULNESS*, and *5d. Tug‐of‐war*, show phenotypes in loss‐of‐function mouse models and/or associations in humans with anxiety (*TCF4* and *MC2R*) and neuroticism (*TCF4*, *CCDC68*, *RAB27B*, and *MBD2*, Table S8). In humans, for *TCF4*, the NHGRI‐EBI Catalog of human genome‐wide association studies (Sollis et al. 2023) reports numerous associations with neuroticism, risk‐taking behavior, and social interaction. Within a region of ~1 kb around the *TCF4* gene, located in dogs on CFA1 (plus strand, Figure 7A) and in humans on chr18 (minus strand, Figure 7B), the variation we found in dogs for BOLDNESS, *DISTANCE PLAYFULNESS*, and *5d* and that associated with neuroticism in humans (Table S10) showed a comparable pattern of distribution with respect to the *TCF4* gene regions. Within the same region, associated SNPs intronic to *RAB27B* are also present in both dogs and humans. In both panels of Figure 7, SNPs are represented with blue and red bars if they are associated with a decrease or an increase in the phenotype, respectively. However, when comparing the two patterns, one must keep in mind that *5d* in the DMA (as an indicator of bold personality with low neuroticism) is the reversed equivalent of human neuroticism, and therefore a different sign is to be expected for a variation with equivalent effect. Additional significant/suggestive SNPs retrieved in different GWAS runs for BOLDNESS and BOLDNESS‐related traits/behavioral variables map to or in proximity to genes for which an association with neuroticism in humans has also been reported (Table S8, Supporting Information Results SR5). These findings support the previous observation that BOLDNESS in dogs is reversely equivalent to neuroticism in humans and point towards the relevance of studying canine behavior for understanding also human personality traits.

**FIGURE 7:**
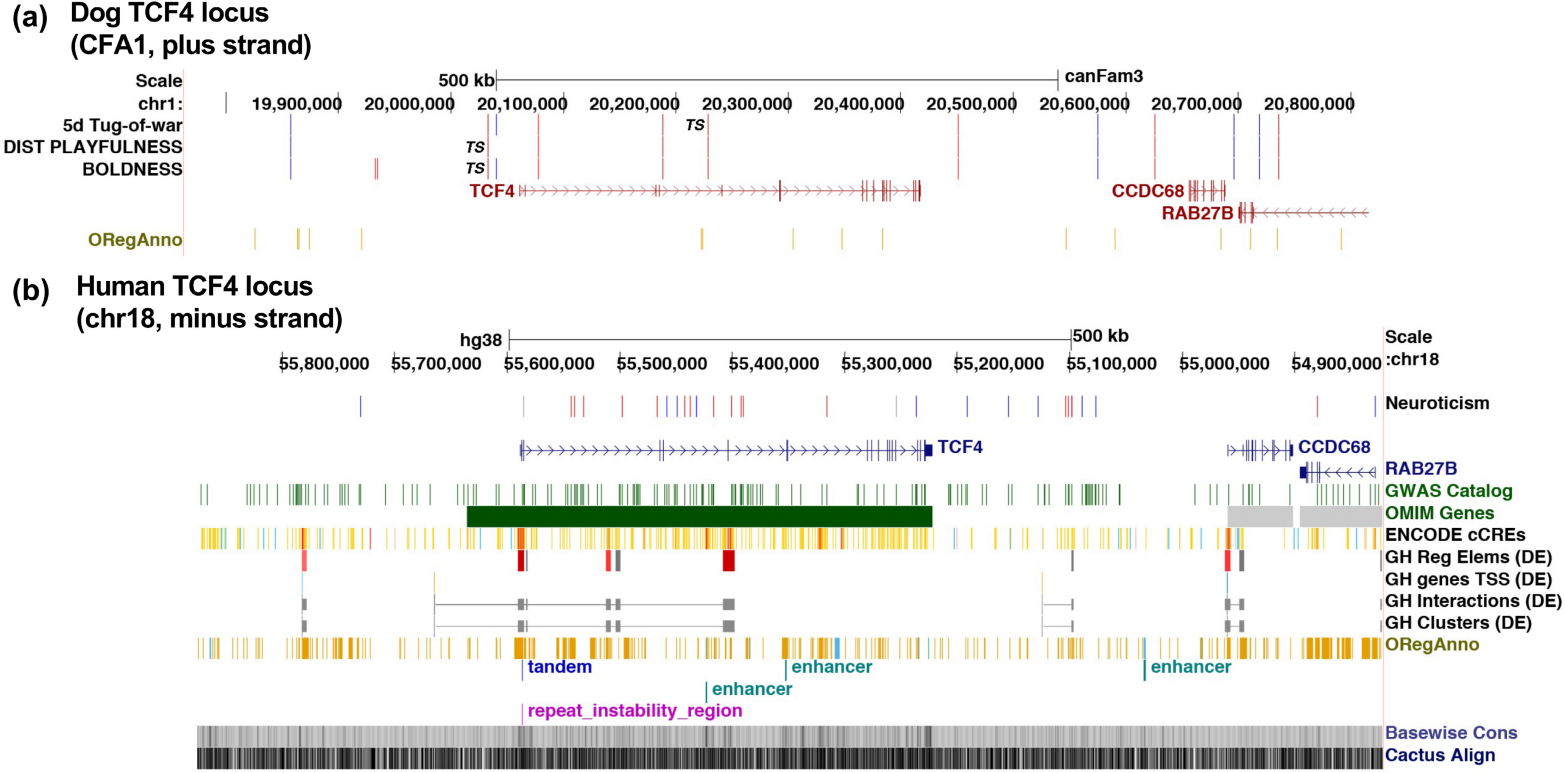
Cross‐reference of variation associated with *5d. Tug‐of‐war, DISTANCE PLAYFULNESS* and BOLDNESS in dogs and neuroticism in humans at the *TCF4* locus. (a) Dog *TCF4* gene locus on CFA1 (plus strand, selected region of 1,053 kb) showing as separate UCSC custom tracks the positions of the significant and suggestive SNPs retrieved for *5d*, *DISTANCE PLAYFULNESS* and BOLDNESS. The top SNP for each trait/behavioral variable is labelled with *TS*. For both species, SNPs are represented with blue and red bars if they are associated with a decrease or increase in phenotype, respectively. However, in dogs, *5d*, *DISTANCE PLAYFULNESS* and BOLDNESS correspond to low neuroticism in humans, and, therefore the sign of a similar phenotypic effect (and thus the color of the bar) will be opposite in the two species. (b) Human *TCF4* gene locus on chromosome 18 (minus strand, selected region of 1,053 kb) with mapped SNPs reported to be associated with neuroticism in the NHGRI‐EBI GWAS catalog (Sollis et al. 2023). SNPs for which information on their phenotypic effect is not available are represented with gray bars. For several SNPs, opposing effects on the same phenotype were found in different studies.

Several aspects could be considered when trying to understand, from a behavioral perspective, the differences between the *working* and the *show* sub‐populations. In the *working* sub‐population, selection for BOLDNESS was stricter and against individuals not able to complete training or perform work duties, while, in an opposite direction, the less bold personality of the *show* type could represent an adaptation. GSDs more likely to be accepted in the Swedish Armed Forces training program for military work display a highly active personality type (Foyer et al. 2014), making them more difficult to be handled by owners and less suitable as companion dogs. In contrast, *show* dogs may have been selected to be more suitable as companion dogs and hence to be less active and easier to handle in the ring and everyday life. In our study, this could be indicated by lower BOLDNESS.

Differences in selective pressures between sub‐populations will also be reflected in the GWAS results. If the selection at a position associated with a trait/behavioral variable is less stringent, the variability in allele frequencies may be higher, thus enabling the GWAS signal to be detected; conversely, stringent selection at that position could lead to fixation, making the signal undetectable or the marker subject to filtration in the preliminary quality control steps.

It is also possible that the behavioral differences, at least partly, occurred inadvertently. In the *show* sub‐population, selection for morphological characteristics based on high conformity to the breed standard (e.g., coat color, curvature of the top line, angulation of the front and hind limbs) might have been predominant. Inadvertently, this could have created more fearful and avoidant dogs, as it was showed in GSDs that some of the selection regions carried associations with both morphological and behavior traits (Friedrich et al. 2020). Genes responsible for the specification of coat color and coat attributes are present within the regions highlighted in our study. *MC2R* (CFA1) is located ~40.9 kb upstream of, and is syntenic in humans, mouse, cattle and dog with the *melanocortin 5 receptor (MC5R*) gene, found in dogs to be associated with fur length and shedding (Hayward et al. 2016). So variation at the *MC2R*‐*MC5R* locus could have been maintained by selection for coat properties. In the absence of phenotypic information on coat length and coat attributes for the dogs in our cohort we could not evaluate whether selection for coat properties has an effect on the signals upstream of the *MC5R* locus including the one at the *TCF4* locus. Future analyses, beyond the scope of this paper, such as a fine mapping of the entire CFA1: 19–26 Mb region and a GWAS performed on a larger, better characterized phenotypical cohort, may provide valuable further insights.

Within the CFA24: 23.5–25.5 Mb region (Figure 3F) the SNP with the highest allele frequency difference between the *working* and the *show* sub‐populations is located 4.2 kb upstream of the transcription start site of the *transient receptor potential cation channel subfamily C member 4 associated protein* (*TRPC4AP*) gene that in mice is associated with skin health and hair follicle cycling (DiTommaso et al. 2014) and has a loss‐of‐function phenotype characterized by abnormal spatial working memory and increased anxiety‐related response, among other defects (Blake et al. 2021). The larger CFA24: 23–26 Mb region contains the *ASIP* gene (Figure 3F) with known roles in coat color specification in mammals, including dogs (Eizirik and Trindade 2021). Variation in two regulatory modules controlling independently the expression of distinct *ASIP* isoforms in the ventral part of the body and in the hair cycle, respectively, gives rise to five distinguishing canine color patterns—dominant yellow, shaded yellow, agouti, black saddle and black back (Bannasch et al. 2021). The three regions of selection in GSDs present within CFA24: 22–26 Mb (*RS1*, *RS2* and *RS3*, Table S5, Figure 3F, Supporting Information Results SR1a) were found to be associated with coat color in British but not Swedish GSDs (Friedrich et al. 2020). In that study, the UK GSDs were primarily pet dogs with predominantly saddle and tan/black and tan coat color, while the Swedish GSDs dogs were all *working* dogs bred for the Swedish Armed Forces with predominant sable coat color (Friedrich et al. 2020). In our analysis, the much more homogenous distribution of coat colors of the *show* sub‐population (Figure 3E) is consistent with the higher average genetic similarity of its cluster in the MDS analysis (Figure 1A,C). Further studies employing low‐coverage genotyping and fine mapping to genotype the causal *ASIP* variants for the different coat color patterns in our cohort would allow for a finer dissection of the coat color distributions in the two sub‐populations.

Also, approximately ~100 kb downstream of the CFA24: 22–26 Mb region, in the GWAS for the entire cohort we identified a suggestive association for *2b.Grabbing* (BICF2S23133032, CFA24: 26,984,165, −log_10_ *p* = 4,13) located in intron 11/31 of the *RALGAPB* gene encoding Ral GTPase Activating Protein Non‐Catalytic Subunit Beta. *RALGAPB* is highly expressed in the human prenatal cortex and *de novo* mutations in the gene were associated with autism spectrum disorder and other related neurodevelopmental disorders such as intellectual disability, developmental delay and epilepsy (Shah et al. 2020).

The CFA24: 23–26 Mb region (Figure 3F) also contains several SNPs and genes associated with hip dysplasia in dogs and humans. A SNP, BICF2S2367279 (ss7212922163), located immediately downstream of the *RS3* region of selection and 25.6 kb upstream of the *src proto‐oncogene, non‐receptor tyrosine kinase* (*SRC*) gene involved in bone formation, had the strongest association with canine hip dysplasia (CHD) in GSDs (Fels and Distl 2014), an association found to be genome‐wide significant also in Samoyed and Labrador retrievers (Mikkola et al. 2021). Therefore, in addition to genetic determinants of behavior, other variation could have been maintained in the sub‐populations due to selection at this region. To understand the role that other genes in this region may have in the morphological and behavioral differences between the *working* and the *show* sub‐populations, it would be necessary to perform further studies including fine mapping of the region in much larger sub‐population cohorts zooming into the genes and positions associated with the highest divergence in allele frequency.

An added layer of complexity in interpreting genetic differences underlying behavioral differences in dogs and especially those between sub‐populations is represented by gene–environment interactions (Sartore et al. 2025). Working and show dogs were subjected to different selection criteria, training and experiences and these could influence DMA results. Even within the working GSD population, it was found that GSDs belonging to owners with prior experience in working dog training (e.g., had trained and competed previously) obtained higher Boldness scores and higher working dog merits (Svartberg 2002). This could be an effect of working dog training prior to the DMA, but it could also be due to selection of the individual (from a specific genetic line, a specific kennel or a specific outstanding puppy in the litter). We did not have information about training and environmental exposure for the dogs in our cohort so we could not take these factors into account in our statistical model or in downstream analyses. However, is it unlikely that these represent a major confounding factor in the interpretation of our results since the GWAS performed on the entire cohort aimed at identifying DMA‐gene associations common to all dogs regardless of training and environmental exposure, while the GWAS performed in the sub‐populations focused on dogs with comparable selection criteria and training.

Our study shows that due to the complexity of the genetic architecture underlying personality traits, it is important to study the individual behavioral variables contributing to a trait and, in the case of breeds with documented population structure, to also look for associations within the respective sub‐populations. While we based our genetic analyses on a limited number of Swedish‐owned GSDs, we show that in terms of trait/behavioral variables scores (Figure S2B), our cohort is representative of the larger group of 23,109 SKK‐registered GSDs. However, as the DMA is administered almost only in Sweden, it is difficult to directly verify these findings using GSDs from other countries.

In numerous instances, signals identified in genome‐wide association studies of dog behavior (Zapata et al. 2016; Ilska et al. 2017; Friedrich et al. 2019; Sarviaho et al. 2020; Morrill et al. 2022; Matsumoto et al. 2023), to cite just a few, map to genes with roles in neurodevelopment and associations with neuropsychiatric disorders in humans, but they rarely converge on the same chromosomal regions. Likely, using much larger cohorts and more similar behavioral tests would yield stronger and more overlapping signals. Our study uses a well‐established system consisting of an extensive battery of behavioral variables objectively measured by trained raters in test conditions that are highly similar. The hierarchical nature of the trait‐behavioral variable relationships in the DMA allows for complex analyses of the different behavioral variables loading on a trait and serves as an internal control for evaluating a signal in the progression from behavioral variable to trait and to the second‐order factor BOLDNESS, as seen in the convergent association signals for *5d*, *DISTANCE PLAYFULNESS* and BOLDNESS, and for several components of *PLAYFULNESS*.

### 4.1 Limitations of the Study

Understanding the genetic determinants of behavior is challenging due to the complexity of behavioral traits, the difficulty of describing them quantitatively and the multiple layers of complexity contributed by the genetic architecture. The DMA used in this study is a well‐established procedure that provides a quantitative description of several personality traits. However, a certain degree of noise inherent to subjective phenotyping is unavoidable in data of this type. This imposes high requirements on analytical tools and may reduce statistical power as compared to phenotypes obtained in conditions where very few variables are out of experimental control. Moreover, behavioral variables are quantified on a discrete, step‐wise scale from 1 to 5, introducing some loss of precision due to discretization. Methods currently used for genome‐wide association analyses are not optimized for this type of scale and this may negatively affect statistical power given certain sample sizes. In addition, polygenic and individual effects (e.g., environment, gene–gene interactions) that involve higher‐order interactions and contribute to the complexity of the genetic architecture underlying personality traits put strong requirements on the sample size necessary to reach statistical power as the effect sizes of particular interactors may be relatively small. Although our sample size of 373 individuals is relatively small compared to the total number of SKK‐registered DMA‐tested GSDs (leading to relatively modest *p*‐values for the reported associations) we show that it is sufficient for detecting loci associated with behavioral variables and personality traits even in the presence of population structure. However, this requires that the structure is properly accounted for, in our case by employing the stratified‐association scheme with population of origin included as a fixed effect in the mixed model and with genetic relatedness modelled as a random effect.

It is worth noticing that the subgroup analyses were based on smaller sample sizes than the primary analyses, resulting in reduced statistical power to detect modest genetic effects. This limitation is particularly relevant given the number of traits and loci evaluated and increases the likelihood of false‐negative findings in the subgroup analyses. Conversely, the large number of association tests creates a potential for false‐positive findings, which we addressed through genome‐wide significance thresholds. Therefore, subgroup results should be interpreted cautiously. In particular, lack of statistical significance should not be interpreted as evidence for the absence of an association. Larger, independently sampled cohorts will warrant confirmation of the observed associations and will enable us to more reliably assess genetic effects within individual subgroups.

## 5 Conclusions

Our study provides a systematic approach for dissecting the complexity of canine behavioral traits and for identifying their genetic determinants. It also provides insights into the effect that selection for *work* versus *show* within the GSD breed has had on behavior related to BOLDNESS. The regions of association we identified for BOLDNESS and its highly related traits/behavioral variables on CFA1 contain genes with documented roles in neurodevelopment and associations with neuroticism in humans, as well as regions of selection for dog domestication and regions of recent selection in GSDs. Given the interpretation of BOLDNESS as a measure of the shyness‐boldness personality continuum in dogs, this highlights the relevance of using the dog as a behavioral genetics model for understanding also human personality traits.

## Author Contributions

**Voichita D. Marinescu:** conceptualization, data curation, formal analysis, investigation, methodology, visualization, writing – original draft, writing – review and editing. **Kenth Svartberg:** conceptualization, formal analysis, investigation, methodology, supervision, writing – original draft, writing – review and editing. **Marcin Kierczak:** conceptualization, data curation, formal analysis, investigation, methodology, visualization, writing – original draft, writing – review and editing. **Katarina Tengvall:** data curation, formal analysis, methodology, resources, writing – review and editing. **Matteo Bianchi:** methodology, writing – review and editing. **Jagoda Jabłońska:** formal analysis, writing – review and editing. **Per Arvelius:** data curation, resources, writing – review and editing. **Sharadha Sakthikumar:** formal analysis. **Fabiana H. G. Farias:** resources. **Åsa Karlsson:** resources. **Mats E. Pettersson:** methodology, writing – review and editing. **Erik Wilsson:** data curation, resources, writing – review and editing. **Erling Strandberg:** writing – review and editing. **Åke Hedhammar:** conceptualization, data curation, supervision, writing – original draft, writing – review and editing. **Kerstin Lindblad‐Toh:** conceptualization, funding acquisition, investigation, supervision, writing – original draft, writing – review and editing. No generative AI or AI‐assisted technologies were used in the research and the preparation of the paper.

## Funding

Computations and data handling were enabled by resources in project SNIC2017/7‐385, provided by the Swedish National Infrastructure for Computing (SNIC) at UPPMAX, partially funded by the Swedish Research Council through grant agreement no. 2018‐05973. KLT is a Distinguished Professor of the Medical Research Council (541‐2013‐8161 and 2023‐00391), a KAW scholar (2023.0307) and a Torsten Söderberg Academy professor in Medicine 2024.

## Conflicts of Interest

The authors declare no conflicts of interest.

## Supporting information


**Data S1:** Supplementary Figures
**Figure S1:** Overview of the project.
**Figure S2:** Factor diagram and correlation coefficients between traits.
**Figure S3:** GWAS results.
**Figure S4:** Genotype–phenotype relationships for SNPs associated with *5d. Tug‐of‐war*, *DISTANCE PLAYFULNESS* and BOLDNESS (pertainig to Supporting Information Results SR3).
**Figure S5:** Significant and suggestive association signals for *PLAYFULNESS* and its contributing behavioral variables.
**Figure S6:** Significant GWAS results in the *show* sub‐population for the *3b. Grabbing 1* behavioral variable contributing to *CHASE‐PRONENESS*.

**Data S2:** Supplementary Tables.
**Table S1:** The Dog Mentality Assessment (DMA).
**Table S2:** Factor loadings and correlation coefficients between traits.
**Table S3:** Boldness relationships and amplitude of score differences between the *working* and the *show* sub‐populations for all traits/behavioral variables.
**Table S4:** Top 100 most divergent SNPs by allele frequency difference between the *working* and the *show* sub‐populations.
**Table S5:** Previously documented regions of selections for domestication, tameness and recent selection in GSDs overlapping regions of interest in our study.
**Table S6:** Significant and suggestive SNPs obtained for the 28 traits/behavioral variables with at least one significant association.
**Table S7:** (A and B) Multi‐trait SNPs retrieved as significant/suggestive for three or more distinct traits/behavioral variables.
**Table S8:** Annotation for association signals obtained for traits/behavioral variables at the extremes of the shyness‐boldness axis.
**Table S9:** Enrichment analyses for the candidate genes.
**Table S10:** Positions and effect of SNPs associated with *5d. Tug‐of‐war, DISTANCE PLAYFULNESS* and BOLDNESS in dogs and neuroticism in humans at the *TCF4* locus.

**Data S3:** Supplementary Methods.

**Data S4:** Supplementary Results.

**Data S5:** File containing the following information for the traits/behavioral variables presented in the main and supplementary Results sections: the distribution of scores in the entire cohort and in the *working* and the *show* sub‐populations; score gradients along the MDS1 dimension in the three sub‐populations; Q‐Q and Manhattan plots for the GWAS results obtained in the three runs: for the entire cohort and the *working* and *show* sub‐populations, respectively; Manhattan plots for the results obtained by meta‐analyses with fixed effects and random effects using the GWAS results obtained for the *working* and the *show* sub‐populations.

## Data Availability

Data used for the analyses could be retrieved from the Supporting Information Tables and from the ScienceLifeLab Data Repository at https://doi.org/10.17044/scilifelab.33411484.
