# Supplementary material for: The Genetic Architecture of Behavior in the German Shepherd Dog
Source: Anim Genet. 2026 Nov 10;57(6):e70221. doi: 10.1002/age.70221 (PMC13752456; doi:10.1002/age.70221)
Supplement: Supplementary file 1 [file AGE-57-0-s001.pdf]

SUPPLEMENTARY METHODS

Behavioral data

All dogs used in this study were assessed using the DMA, a behavioral evaluation developed and used by the Swedish Working Dog Association mainly as a tool for breeding of working dogs (Svartberg, K. and Forkman, B. 2002). In the DMA, the dog is exposed to different standardized situations in 10 subtests and its behavioral reactions are assessed by a trained rater according to a protocol containing 33 items (here referred to as behavioral variables). Administered with only short breaks between subtests, the DMA takes 40-50 minutes to complete per dog and involves the dog (of minimum 12 months of age), a handler (often the owner), an assessment leader, the rater and several figurants. The ratings are made as objectively as possible according to the intensity of the behavioral reaction on a scale from 1 to 5, where a low score equals a low intensity reaction. A detailed description of the DMA is presented in Table S1 that follows the description in (Svartberg, K. and Forkman, B. 2002) and (Svartberg, K. 2022), as well as the description of the Swedish Working Dog Association (SWDA 2021). A video recording of the DMA could be viewed at this link: https://vimeo.com/917811308/11bb0a046b.

Exploratory Factor Analyses (EFA)

To identify factors and loadings specific to the GSD breed, we used as input standardized scores for all DMA behavioral variables (Table S1) for 23,109 GSDs registered in the Swedish Kennel Club (SKK) and performed EFA in the R statistical analyses suite (Team, R.C. 2019) version 3.6.1 with the package *psych* version 2.1.9 (Revelle, W. 2021). We computed the polychoric correlation matrix (function *psych::polychoric*) for all 33 behavioral variables in the DMA and used it to identify 1) six first order factors, number supported by the Velicer's minimum average partial (VMAP) test and previous studies (function *psych::fa*, oblimin rotation), and 2) one second-order factor, BOLDNESS (function *psych::fa.multi,* oblimin rotation). The first-order factors were identified based on previous reports (Svartberg, K. and Forkman, B. 2002) as being *SOCIABILITY, PLAYFULNESS*, *CHASE-PRONENESS, DISTANCE PLAYFULNESS*, *CURIOSITY/FEARLESSNESS* and *AGGRESSIVENESS*. The behavioral variable scores were transformed into *Z*-scores prior to computing the factor scores. For each dog, aggregated scores for the first order factors were computed as a weighted sum across all 33 behavioral variables and all loadings, *i.e.* the sum of the product between the standardized value for each variable and its loading. The values for the second-order factor BOLDNESS were computed as the weighted sum of the scores of the six first order factors multiplied by the loadings for BOLDNESS. For *CURIOSITY/FEARLESSNESS* the sign of the weighted sum was multiplied by -1 such that high values expressed high curiosity and fearlessness. The resulting aggregated scores for the first and second order factors were standardized again across the 23,109 GSDs. From the

resulting standardized trait/behavioral variables scores, we extracted the values for the 373 GSDs for which genotype data was available and used them for further analyses and in the GWAS. To check whether our GWAS cohort was a representative set for the larger SKK GSD population, we performed EFA and computed factor loadings in the same manner but using as input only the DMA scores for the 373 genotyped GSDs.

Study population

In the current study, we included 373 German Shepherd Dogs (GSD) genotyped in three different batches: from the Swedish Armed Forces training program (*n=14*, Batch_1 in the dataset), from privately-owned individuals (*n=237*, Batch_2) collected as part of another project (Tengvall, K. *et al*. 2013) and from privately-owned individuals selected specifically for this project (*n=122*, Batch_3). Batch was used as a covariate in all GWAS analyses. Before assembling the final cohort, dogs were filtered to eliminate individuals with first degree relatedness using *king* (Manichaikul, A. *et al*. 2010) and sex-check discrepancies using *plink2* (Chang, C.C. *et al*. 2015) with the "--dog" option. Dogs younger than 1 year and older than 3 years at the time of the DMA were also eliminated. The female/male ratio was F/M = 2 for the entire cohort of 373 GSD dogs, F/M = 1.5 and F/M = 3.6 for the *working* and *show* sub-populations, respectively. This bias is due to the fact that samples used in the study were collected primarily from breeding colonies where many more females are used and tested in the DMA. However, the relationship between working dog performance and DMA-Boldness on an individual level has been identified in both GSD males and females (Svartberg, K. 2002) and further analyses on first order traits (Svartberg K, personal communication) showed that we expect to find the same relationship between DMA traits and working performance in both sexes. Moreover, in all GWAS runs in which a statistically significant correlation was observed between the DMA score for the trait/behavior variable and sex (Table S6), sex was used as a covariate in the statistical model.

The age in days at the time of the DMA had a median $age_{md} = 496$, a mean $age_{m} = 538$ and a standard deviation $age_{sd} = 136.5$. The corresponding values per genotyping batch were: Batch_1 (F/M=1.3, $age_{md} = 643$, $age_{m} = 673.1$, $age_{sd} = 203.2$), Batch_2 (F/M = 2.6, $age_{md} = 499$, $age_{m} = 539.4$, $age_{sd} = 137.5$) and Batch_3 (F/M = 1.4, $age_{md} = 485$, $age_{m} = 519.8$, $age_{sd} = 116.2$).

Phenotypes and covariates

To examine which additional variables may influence the traits/behavioral variables we performed additional non-parametric tests. Specifically, for each trait/behavioral variables we checked in entire cohort, and in the *working* and *show* sub-populations separately, whether sex of an individual (Wilcoxon's test with continuity correction) and the logarithm of the age in days at the time of DMA (Kendall rank correlation coefficient) have a significant effect on the traits. If so, the parameter was

used as a covariate in the GWAS analysis. The sample batch was used as a covariate in all runs. Covariates used for the traits/behavioral variables showing at least one significant association in any of the runs are listed in Table S6. Coat color designations for the dogs in our study represent the English translations of the Swedish coat color names as they appear in the SKK database (SKK Hunddata, https://hundar.skk.se/hunddata/). These coat color descriptions are supplied by the dog owners and are not verified further by the SKK.

SNP genotyping and quality control

We isolated genomic DNA from whole blood samples using QIAmp DNA Midi kit (Qiagen Inc., CA, USA) and genotyped it using the Illumina 170K CanineHD Bead Chip (Illumina, San Diego, CA) resulting in a mean call rate of 99.7% (SD = 0.7%) across all SNPs. Throughout this study, we used SNP coordinates based on the CanFam3.1 reference genome assembly (Hoeppner, M.P. *et al*. 2014). Genotype data from three different genotyping batches were merged in R version 3.6.1 (Team, R.C. 2019) using the *GenABEL::merge.gwaa.data* function from the *GenABEL* package version 1.8-0 (Aulchenko, Y.S. *et al*. 2007). We performed preliminary quality control (QC1) using standard *GenABEL* functions. We filtered out all markers with minor allele frequency (MAF) smaller than 1% as non-informative. We also removed all markers with call-rate below 95% and all individuals with per-individual call-rate lower than 95%. From all pairs of individuals sharing 95% or more markers IBS (identity-by-state, weighted by allelic frequencies), we removed the individuals with lower call-rate. Individuals with too high (FDR = 0.01) autosomal heterozygosity were also excluded. IBS checks were based on 2,000 autosomal markers randomly selected across the entire autosomal genome. We used relaxed thresholds for QC1, to avoid losing false negatives given the limited size of our cohort, but later, when analyzing the GWAS results, we ran additional quality controls on the results (see below).

Detecting population structure

Following the initial quality control (QC1) step, we computed a pairwise genomic-kinship matrix using the *GenABEL::ibs* function with weights proportional to the observed allele frequencies. Next, we transformed the computed genomic-kinship matrix into a distance matrix and applied multi-dimensional scaling (MDS) to project pairwise (between-individuals) genomic distances in two dimensions. After visualizing the distances, we noticed a stratification of the study population in line with previous reports (Tengvall, K. *et al*. 2013) and we verified it using model-based clustering. We used finite normal mixture modeling as implemented in the R package *mclust* version 5.4.9 (Scrucca, L. *et al*. 2016). To select the optimal model and the number of clusters, we used Bayesian Information Criterion—BIC (Fraley, C. *et al*. 2012). Using the optimized model, we assigned each individual to a cluster. For the subsequent subpopulation-wise genome-wide analyses, we treated

individuals with cluster-assignment uncertainty ≥ 95th percentile as a separate sub-population (*border*, Fig. 1A-B); dogs in the *border* sub-population were not included in the GWAS analyses. To identify the gradient in scores across the first MDS dimension (MDS1) we divided the cohort in five sets (Fig. 1E) based on the MDS1 values: *working*/*show* sub-population dogs with low/high MDS1 values and the *border* subpopulation (RL1 regression line, Fig. 1E). To account for the slight overlap of sub-populations within the *border* segment, we also performed the analysis by dividing the cohort in 5 groups based solely on partitioning the MDS1 values in 5 bins (RL2 regression line, Fig. 1E). The values for the intercept and slope for the regression lines obtained by the two methods are almost identical for all traits/behavioral variables.

Identifying the genetic source of the population structure

To better understand the source of the observed population structure, we implemented a procedure for detecting allele frequency differences between the well-defined *working* and *show* sub-populations. We eliminated the *border* sub-population ($n = 19$, 5% of the dogs) from analysis due to its uncertain sub-population assignment. At each position in the genome, we compared the observed sub-population-specific counts of an arbitrarily chosen reference allele to the counts expected under the null H_0: *no difference in allele counts between subpopulations* and we tested the significance of the observed allele-count differences between the sub-populations using Fisher's exact test for counts. We used an arbitrary threshold of $-\log10p \geq 20$ to select positions with greatest allele frequency differences. Absolute differences in major allele frequencies for regions of interest were also plotted by smoothing their values using a sliding overlapping window approach over a window size of 21 markers (10 upstream and 10 downstream of the central marker) and with a 1 marker step. Values for the fixation index F_{ST} were computed using the *compute.fstats* function in the R package *cgmisc* version 2.9.11 (Kierczak, M. *et al*. 2015) using the parameter *est.type = 'Hudson'*. For the sake of reproducibility, both *GenABEL* and *cgmisc* packages are available as a Docker container and can be pulled directly from GitHub ghcr.io repository. For details see https://github.com/cgmisc-team/cgmisc.

Genome-wide association analyses

We performed all analyses using R version 3.6.1 (Team, R.C. 2019) and the *GenABEL* R package, version 1.8-0 (Aulchenko, Y.S. *et al*. 2007). To assess the level of genomic inflation, we measured the genomic inflation factor **λ** and visualized observed *vs*. expected distribution of *p* values using quantile-quantile (Q-Q) plots.

In order to account for population structure and potential cryptic relatedness, we used a two-stage hierarchical linear mixed model (LMM) framework (implemented using *GenABEL*) in our association analyses. First, we modelled the degree of genetic relatedness as a random effect using

the pairwise genomic-kinship matrix derived from clean SNP data with weights proportional to observed allele frequencies. This is a standard approach to constructing GRM (Genomic Relationship Matrix) used for capturing polygenic variance and controlling for cryptic relatedness, technically equivalent to GRM used in other popular linear-mixed model frameworks like *GEMMA* (Zhou, X. and Stephens, M. 2012) or *GCTA* (Yang, J. *et al.* 2011). In addition, to explicitly account for the pronounced macro-stratification between *working* and *show* lineages (Fig. 1A), we first fitted an LMM incorporating the trait covariates, batch, and a GRM random effect. To further account for residual lineage-specific allele-frequency differences while avoiding collinearity in the primary LMM, association tests were subsequently performed on the residuals from this model, with sub-population cluster assignment (derived using *mclust*) included as a fixed effect. This two-step strategy is functionally analogous to standard LMM approaches that combine a GRM, capturing fine-scale genetic relatedness as a random effect, with principal components or discrete lineage clusters, capturing broader population stratification as fixed effects. It therefore provides robust control of false-positive associations arising from the pronounced lineage structure. This was confirmed by the fact that the Q-Q plots obtained in the GWAS on the entire cohort show no inflation after the stratification procedure (Supplementary Information File 5).

Also, to account for the complex population structure, we established the significance of the obtained results in an empirical way, by performing permutation tests on mixed model residuals. We performed n = 1,000 permutations, each involving evaluation of all markers against the permuted phenotypes, and used the distribution of the *minimal p* values, *i.e.* the lowest single-marker p observed in each permutation, to set our significance threshold (at α = 5%) using Student's t-test. These *minimal p* values are inherently adjusted for the number of markers tested, since each value represents the extreme outcome out of an entire permutation. Also, we did not apply a correction for multiple testing for the number of GWAS we ran, since the DMA traits/behavioral variables analyzed are correlated and inter-dependent and cannot be considered independent. We also checked that the p value of a significantly associated marker is also lower than the threshold based on the average haploblock size of 1Mb in a dog breed's genome (Lindblad-Toh, K. *et al.* 2005). For ~2.4Gb of the canine genome this yields ~2,400 independent haplotype blocks. At α=5% the threshold for genome-wide significance becomes: $-\log_{10}(0.05/2400) = 4.7$. We considered, as suggestive, associated SNPs with $-\log_{10}p$ greater than 4.

Data for the entire cohort and for the *working* and the *show* sub-population, respectively, was treated as three separate datasets (*i.e.* subjected to their own quality control, covariate analyses and significance thresholding) and for each trait/behavioral variable the GWAS was run separately on the entire cohort and the two sub-populations. For each trait/behavioral variable, METASOFT 2.0.1 (Han, B. and Eskin, E. 2011) was used to perform meta-analyses with fixed and random effects using as input the GWAS results obtained for the *working* and the *show* sub-populations, in order to

compare the results with those obtained in the GWAS for the entire cohort. For the traits/behavioral variables presented in Results, Manhattan plots for the meta-analyses results are presented alongside the GWAS results in Supplementary Information File 5. Given that we used relaxed thresholds for QC1 as not to lose weak signals, before interpreting the results we eliminated from further analyses SNPs outside the Hardy-Weinberg equilibrium (FDR < 0.2) and SNPs with MAF < 5% in all three GWAS runs as they could represent possible false positives. Nineteen significant SNPs that passed the threshold of significance in their respective runs but have MAF < 5% in all three GWAS runs are listed in italics at the end of Table S6 as candidates that would require further validation before being considered. The R package *CMplot* version 4.0.0 (LiLin, Y. 2022) was used to generate the plots for the SNP densities and the Manhattan plots for the allele frequency difference scan and meta-analyses results. For several plots the R package *ggplot2* (Wickham, H. 2016) version 3.3.5 was also used.
To avoid the high rates of genotyping errors, ambiguous probe mapping and statistical confounding caused by hemizygosity in males leading to false-positive associations, X-chromosome markers were omitted from the kinship matrix construction, and any associated signals were eliminated from downstream analyses.

Cross-reference with mouse and human data

All mouse loss-of-function phenotypes presented in Table S8 were retrieved from the Mouse Genome Database (Blake, J.A. *et al.* 2021). Human GWAS data consisting of SNPs reported as being associated with different traits were retrieved from the NHGRI-EBI Catalog of human genome-wide association studies (Sollis, E. *et al.* 2023) by querying for genes and genomic regions of interest. Gene associations with neuropsychiatric disorders in humans were retrieved from the GeneCards (Stelzer, G. *et al.* 2016) and the MalaCards (Rappaport, N. *et al.* 2017) databases.
To compare, at the *TCF4* gene locus, the distribution of variation we found for BOLDNESS, *DISTANCE PLAYFULNESS* and *5d. Tug-of-war* in dogs with the variation associated with neuroticism in humans (Fig. 7), we zoomed in on a sequence of 1,053kb encompassing the *TCF4* gene. Within it, for each trait, we indicated by use of a custom UCSC track, the positions of variants with bars color-coded based on the reported phenotypic effect of the variant. This was inferred from the sign of its *effB* coefficient or a similar measure reported and was indicated with blue for a decrease in phenotype, red for an increase and gray if this information was not available.

Enrichment analyses

To assemble the list of candidate genes for enrichment analyses we used the SNP annotations in Table S8 taking into account either the gene in which the marker is located or, if the marker falls within an intragenic region, the first gene immediately upstream and downstream from it. The resulting list of 54 unique genes was submitted to *Enrichr* (Kuleshov, M.V. *et al.* 2016) to search for

statistically significant overrepresentation ($p < 0.05$, minimum overlap of two candidate genes) against knowledge databases such as KEGG pathways (Kanehisa, M. and Goto, S. 2000), Gene Ontology (Ashburner, M. *et al*. 2000, Gene Ontology, C. 2026), ARCHS4 Tissues (Lachmann, A. *et al*. 2018) and DisGeNET (Pinero, J. *et al*. 2020).

References

Ashburner, M., Ball, C.A., Blake, J.A., Botstein, D., Butler, H., Cherry, J.M., . . . Sherlock, G. (2000). Gene ontology: Tool for the unification of biology. *Nature Genetics* **25**(1): 25-29. *10.1038/75556*.

Aulchenko, Y.S., Ripke, S., Isaacs, A. and van Duijn, C.M. (2007). Genabel: An r library for genome-wide association analysis. *Bioinformatics* **23**(10): 1294-1296. *10.1093/bioinformatics/btm108*.

Blake, J.A., Baldarelli, R., Kadin, J.A., Richardson, J.E., Smith, C.L., Bult, C.J. and Mouse Genome Database, G. (2021). Mouse genome database (mgd): Knowledgebase for mouse-human comparative biology. *Nucleic Acids Res* **49**(D1): D981-D987. *10.1093/nar/gkaa1083*.

Chang, C.C., Chow, C.C., Tellier, L.C., Vattikuti, S., Purcell, S.M. and Lee, J.J. (2015). Second-generation plink: Rising to the challenge of larger and richer datasets. *GigaScience* **4**(1): 13742-13015. *10.1186/s13742-015-0047-8*.

Fraley, C., Raftery, A., Murphy, T. and Scrucca, L. (2012). Mclust version 4 for r: Normal mixture modeling for model-based clustering, classification, and density estimation. *Technical Report No. 597*.

Gene Ontology, C. (2026). The gene ontology knowledgebase in 2026. *Nucleic Acids Res* **54**(D1): D1779-D1792. *10.1093/nar/gkaf1292*.

Han, B. and Eskin, E. (2011). Random-effects model aimed at discovering associations in meta-analysis of genome-wide association studies. *Am J Hum Genet* **88**(5): 586-598. *10.1016/j.ajhg.2011.04.014*.

Hoeppner, M.P., Lundquist, A., Pirun, M., Meadows, J.R., Zamani, N., Johnson, J., . . . Grabherr, M.G. (2014). An improved canine genome and a comprehensive catalogue of coding genes and non-coding transcripts. *PLoS One* **9**(3): e91172. *10.1371/journal.pone.0091172*.

Kanehisa, M. and Goto, S. (2000). Kegg: Kyoto encyclopedia of genes and genomes. *Nucleic Acids Res* **28**(1): 27-30. *10.1093/nar/28.1.27*.

Kierczak, M., Jablonska, J., Forsberg, S.K., Bianchi, M., Tengvall, K., Pettersson, M., . . . Lindblad-Toh, K. (2015). Cgmisc: Enhanced genome-wide association analyses and visualization. *Bioinformatics* **31**(23): 3830-3831. *10.1093/bioinformatics/btv426*.

Kuleshov, M.V., Jones, M.R., Rouillard, A.D., Fernandez, N.F., Duan, Q., Wang, Z., . . . Ma'ayan, A. (2016). Enrichr: A comprehensive gene set enrichment analysis web server 2016 update. *Nucleic Acids Res* **44**(W1): W90-97. *10.1093/nar/gkw377*.

Lachmann, A., Torre, D., Keenan, A.B., Jagodnik, K.M., Lee, H.J., Wang, L., . . . Ma'ayan, A. (2018). Massive mining of publicly available rna-seq data from human and mouse. *Nat Commun* **9**(1): 1366. *10.1038/s41467-018-03751-6*.

LiLin, Y. (2022). Cmplot: Circle manhattan plot.

Lindblad-Toh, K., Wade, C.M., Mikkelsen, T.S., Karlsson, E.K., Jaffe, D.B., Kamal, M., . . . Lander, E.S. (2005). Genome sequence, comparative analysis and haplotype structure of the domestic dog. *Nature* **438**(7069): 803-819. *10.1038/nature04338*.

Manichaikul, A., Mychaleckyj, J.C., Rich, S.S., Daly, K., Sale, M. and Chen, W.M. (2010). Robust relationship inference in genome-wide association studies. *Bioinformatics* **26**(22): 2867-2873. *10.1093/bioinformatics/btq559*.

Pinero, J., Ramirez-Anguita, J.M., Sauch-Pitarch, J., Ronzano, F., Centeno, E., Sanz, F. and Furlong, L.I. (2020). The disgenet knowledge platform for disease genomics: 2019 update. *Nucleic Acids Res* **48**(D1): D845-D855. *10.1093/nar/gkz1021*.

Rappaport, N., Twik, M., Plaschkes, I., Nudel, R., Iny Stein, T., Levitt, J., . . . Lancet, D. (2017). Malacards: An amalgamated human disease compendium with diverse clinical and genetic annotation and structured search. *Nucleic Acids Research* **45**(D1): D877-D887. *10.1093/nar/gkw1012*.

Revelle, W. (2021). Psych: Procedures for psychological, psychometric, and personality research.

Scrucca, L., Fop, M., Murphy, T.B. and Raftery, A.E. (2016). Mclust 5: Clustering, classification and density estimation using gaussian finite mixture models. *The R Journal* **8**(1): 289-317. *10.32614/RJ-2016-021*.

Sollis, E., Mosaku, A., Abid, A., Buniello, A., Cerezo, M., Gil, L., . . . Harris, L.W. (2023). The nhgri-ebi gwas catalog: Knowledgebase and deposition resource. *Nucleic Acids Res* **51**(D1): D977-D985. *10.1093/nar/gkac1010*.

Stelzer, G., Rosen, N., Plaschkes, I., Zimmerman, S., Twik, M., Fishilevich, S., . . . Lancet, D. (2016). The genecards suite: From gene data mining to disease genome sequence analyses. *Current Protocols in Bioinformatics* **54**(1): 1.30.31-31.30.33. *10.1002/cpbi.5*.

Svartberg, K. (2002). Shyness–boldness predicts performance in working dogs. *Applied Animal Behaviour Science* **79**(2): 157-174. *10.1016/S0168-1591(02)00120-X*.

Svartberg, K. (2022). A possible basis for personality in dogs: Individual differences in affective predispositions. *Applied Animal Behaviour Science* **255**: 105740. *doi.org/10.1016/j.applanim.2022.105740*.

Svartberg, K. and Forkman, B. (2002). Personality traits in the domestic dog (*canis familiaris*). *Applied Animal Behaviour Science* **79**(2): 133-155. *10.1016/S0168-1591(02)00121-1*.

SWDA (2021). Utförandeanvisningar för mentalbeskrivning hund (mh). Manual by the swedish working dog association. . *https://brukshundklubben.se/media/rb5blgri/utf%C3%B6randeanvisningar-mh-2021-01-01.pdf*.

Team, R.C. (2019). R: A language and environment for statistical computing.

Tengvall, K., Kierczak, M., Bergvall, K., Olsson, M., Frankowiack, M., Farias, F.H., . . . Lindblad-Toh, K. (2013). Genome-wide analysis in german shepherd dogs reveals association of a locus on cfa 27 with atopic dermatitis. *PLoS Genet* **9**(5): e1003475. *10.1371/journal.pgen.1003475*.

Wickham, H. (2016). Ggplot2: Elegant graphics for data analysis.

Yang, J., Lee, S.H., Goddard, M.E. and Visscher, P.M. (2011). Gcta: A tool for genome-wide complex trait analysis. *The American Journal of Human Genetics* **88**(1): 76-82. *10.1016/j.ajhg.2010.11.011*.

Zhou, X. and Stephens, M. (2012). Genome-wide efficient mixed-model analysis for association studies. *Nat Genet* **44**(7): 821-824. *10.1038/ng.2310*.
