# Supplementary material for: The Genetic Architecture of Behavior in the German Shepherd Dog
Source: Anim Genet. 2026 Nov 10;57(6):e70221. doi: 10.1002/age.70221 (PMC13752456; doi:10.1002/age.70221)

Supplementary Score Distributions and Gradients, Q-Q and Manhattan Plots

This file presents the following information for the traits/behavioral variables presented in the main and supplementary Results sections:

- the distribution of scores (original scores for behavioral variables and standardized weighted sum scores for traits and BOLDNESS) in the entire cohort and in the *working* and the *show* sub-populations.

- score gradients along the MDS1 dimension in the three sub-populations. Each dot corresponds to the score of one individual. Perpendicular bars represent division points within sub-populations based on low versus high MDS1 values. Blue filled circles represent score group means and triangles mark the positions of the first and third quartile within each distribution. Blue (RL1) and green (RL2) lines represent regression lines fit through the resulting group mean points obtained by dividing the cohort in five groups (see Supplementary Information Methods).

- Q-Q and Manhattan plots for the GWAS results obtained in the three runs: for the entire cohort and the *working* and the *show* sub-populations, respectively. Red lines mark the significance threshold determined by 1000 permutations. Blue lines mark the threshold for considering a SNP suggestive ($-\log_{10} p = 4$).

- Manhattan plots for the results obtained by meta-analyses with fixed effects and random effects using the GWAS results obtained for the *working* and the *show* sub-populations. Blue and red lines drawn at $-\log_{10} p = 4$ and $-\log_{10} p = 5$, respectively, are only for orientation and comparison.

Second-order trait: BOLDNESS

SCORE DISTRIBUTIONS

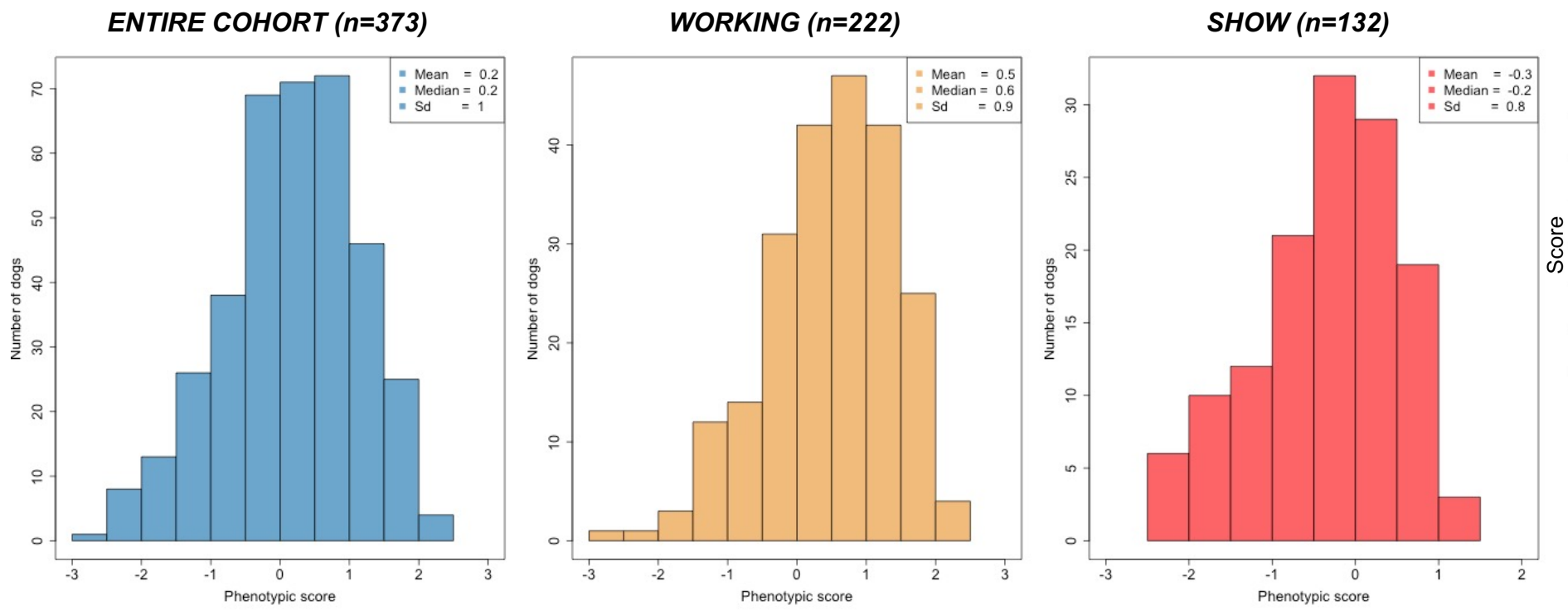

SCORE GRADIENT

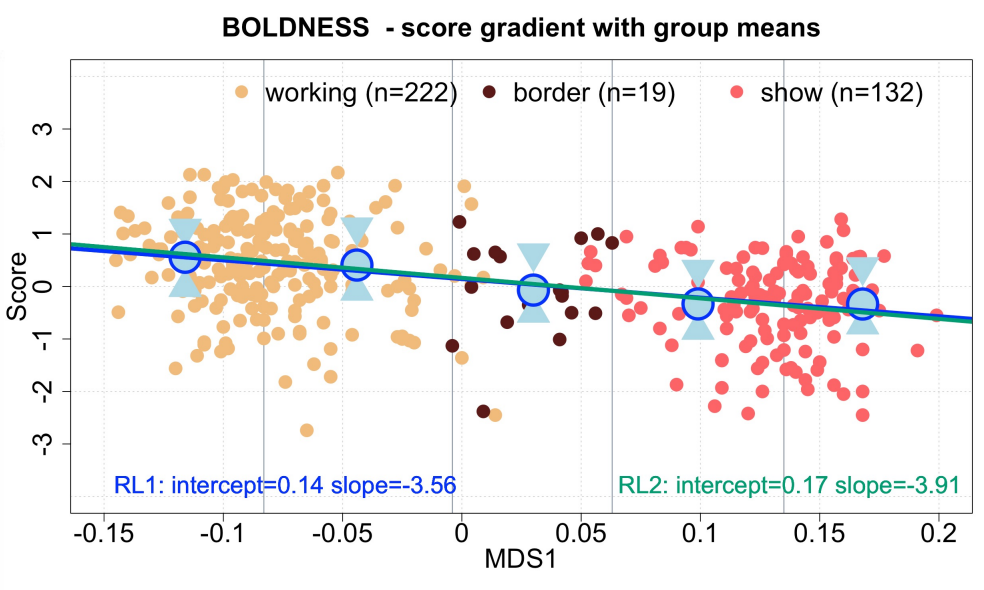

GWAS ENTIRE COHORT

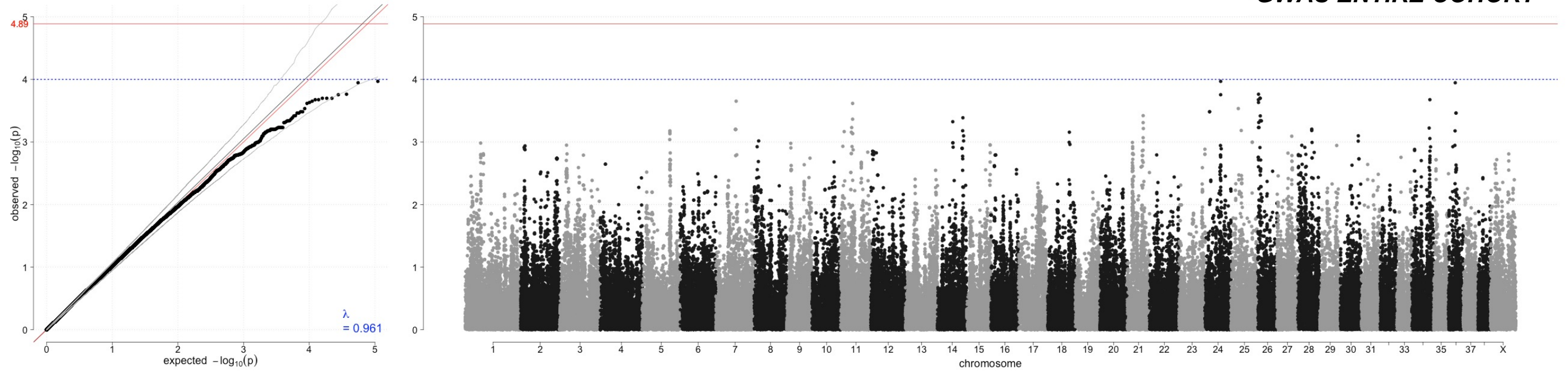

GWAS WORKING

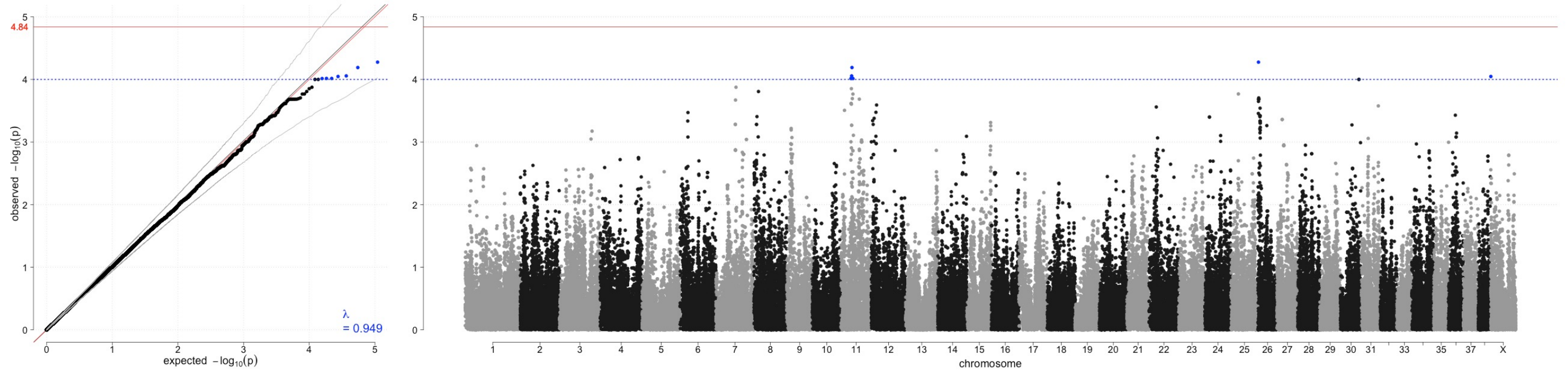

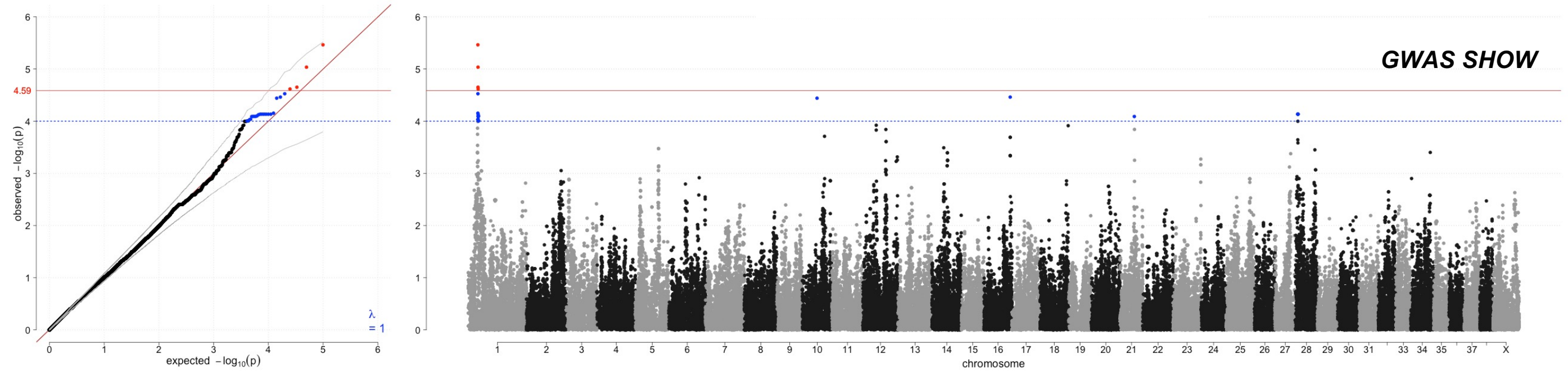

FOR GWAS RESULTS OBTAINED FOR THE WORKING AND SHOW SUB-POPULATIONS:

META-ANALYSIS FIXED EFFECTS

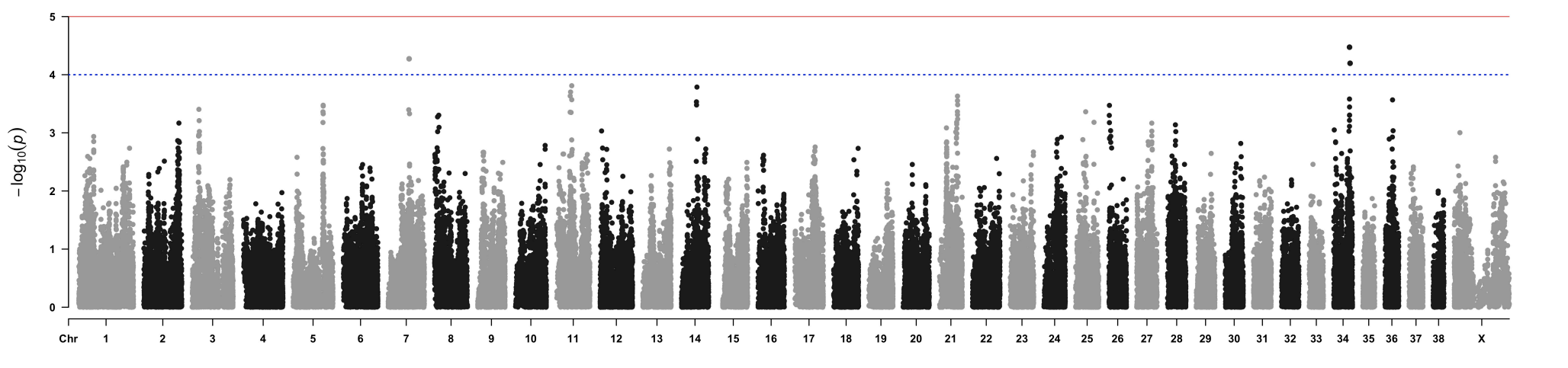

META-ANALYSIS RANDOM EFFECTS

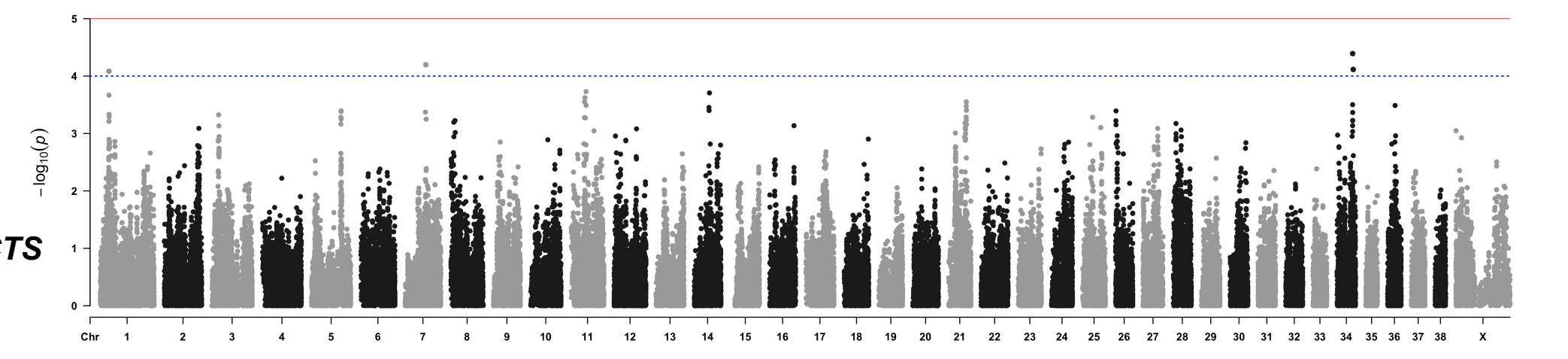

Trait: DISTANCE PLAYFULNESS

SCORE DISTRIBUTIONS

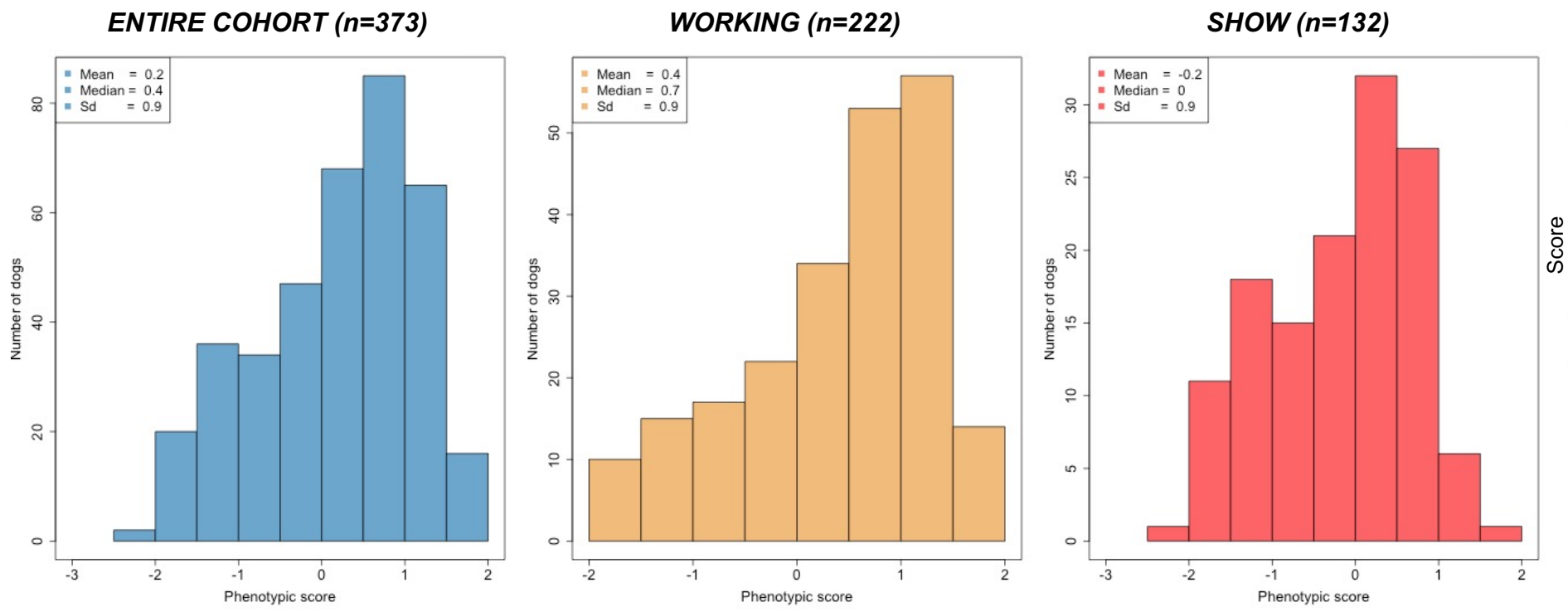

SCORE GRADIENT

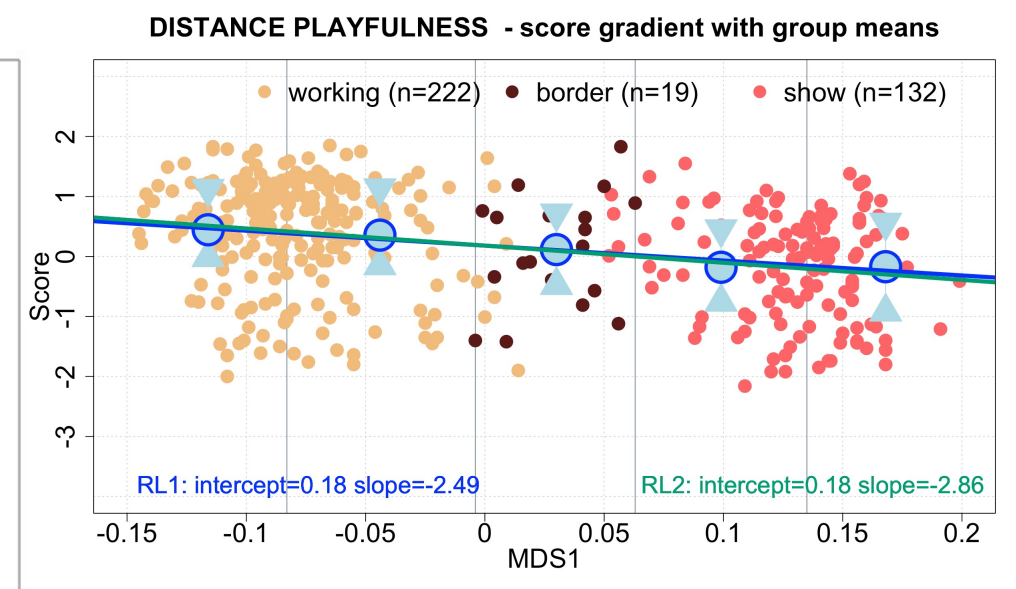

GWAS ENTIRE COHORT

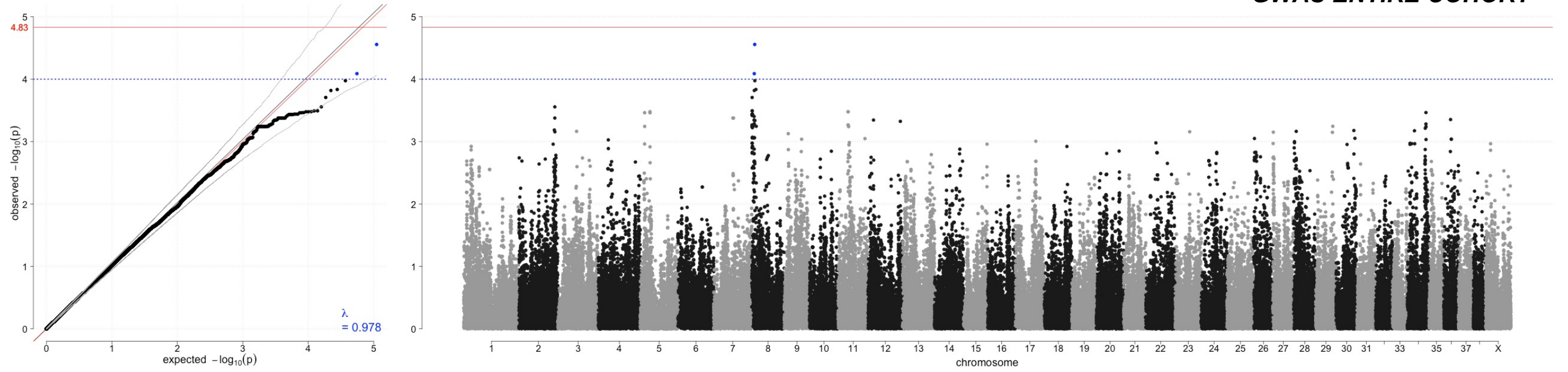

GWAS WORKING

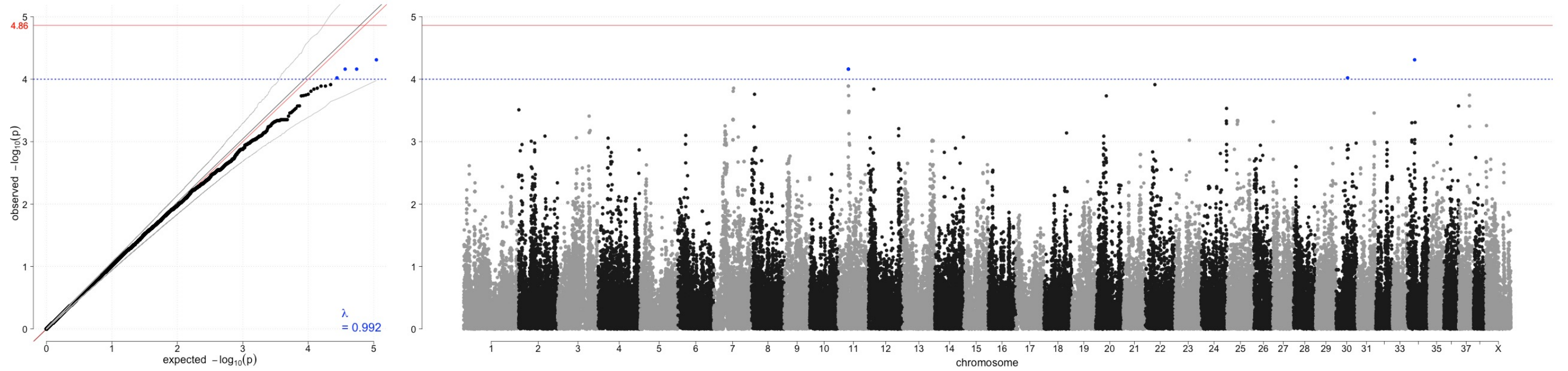

GWAS SHOW

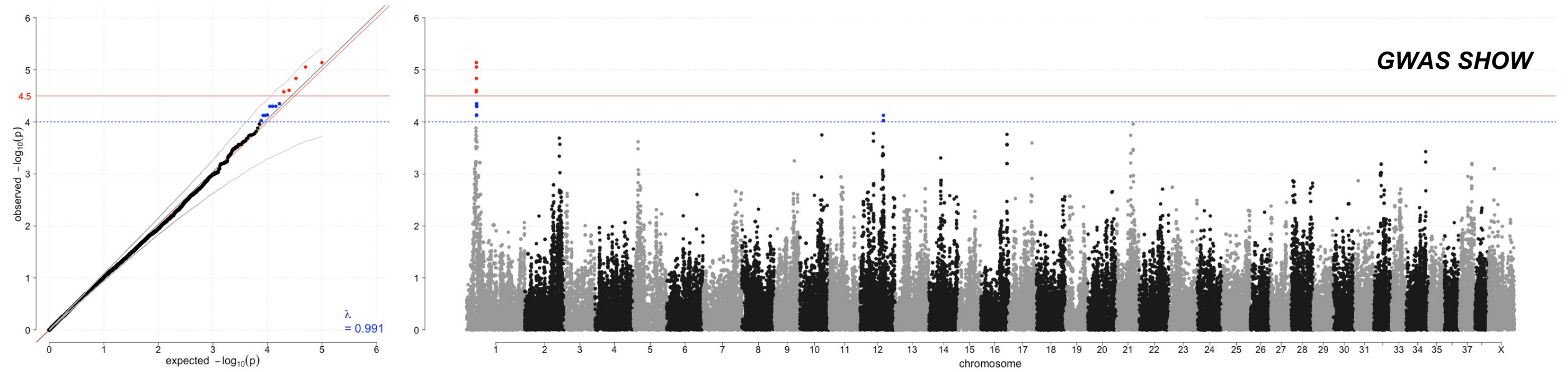

FOR GWAS RESULTS OBTAINED FOR THE WORKING AND SHOW SUB-POPULATIONS:

META-ANALYSIS FIXED EFFECTS

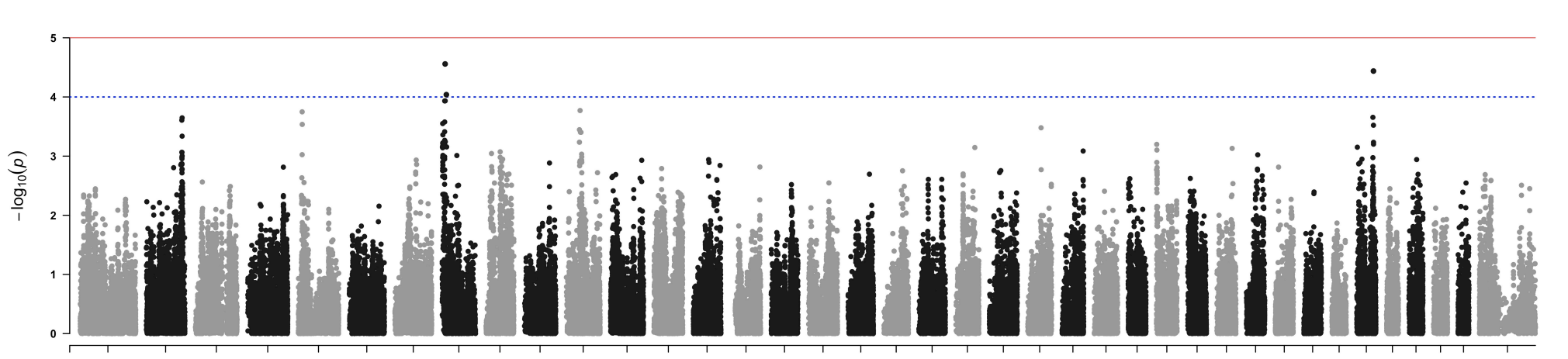

META-ANALYSIS RANDOM EFFECTS

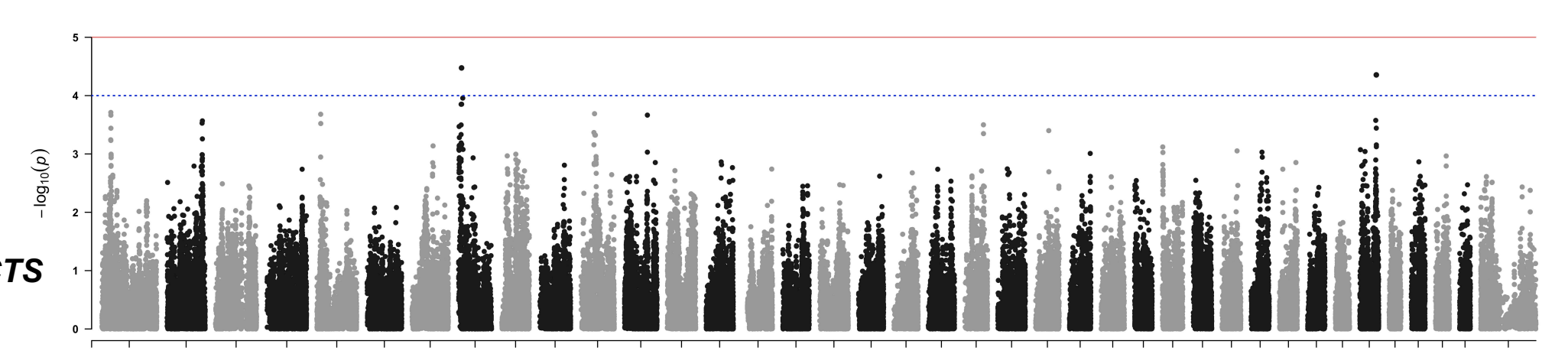

Behavioral variable: 5d. Tug-of-war

SCORE DISTRIBUTIONS

ENTIRE COHORT (n=373) | WORKING (n=222) | SHOW (n=132)

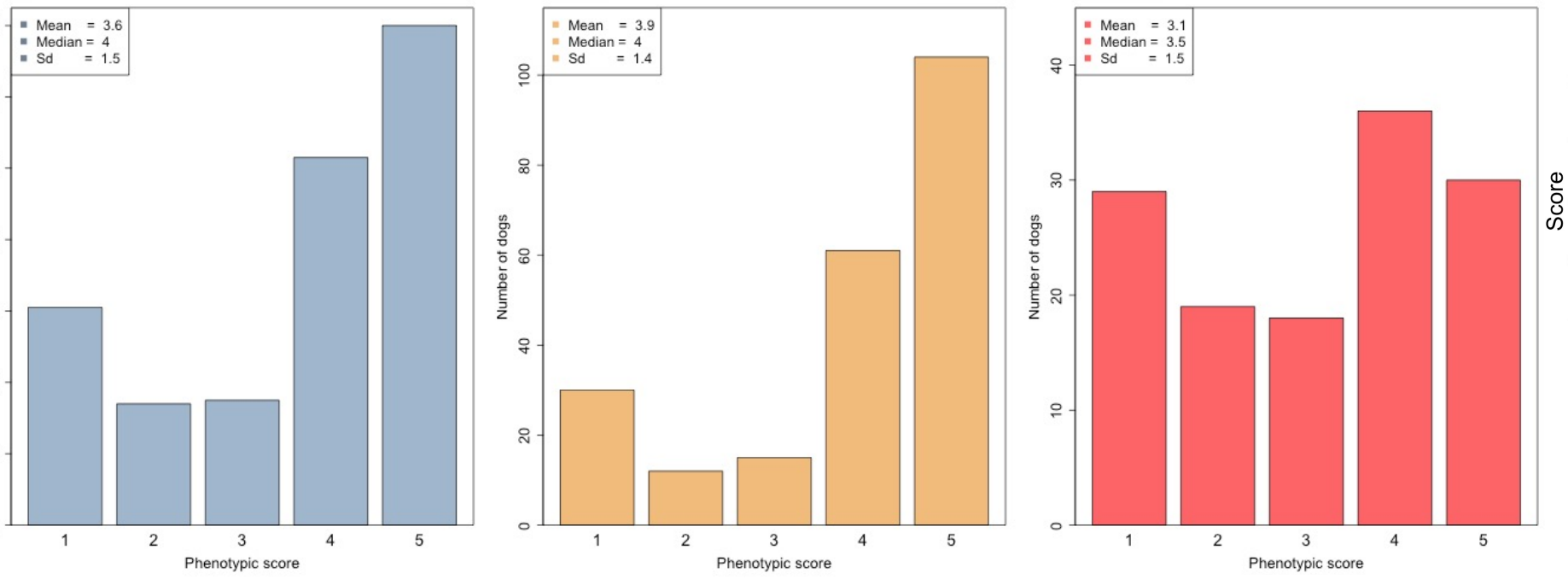

SCORE GRADIENT

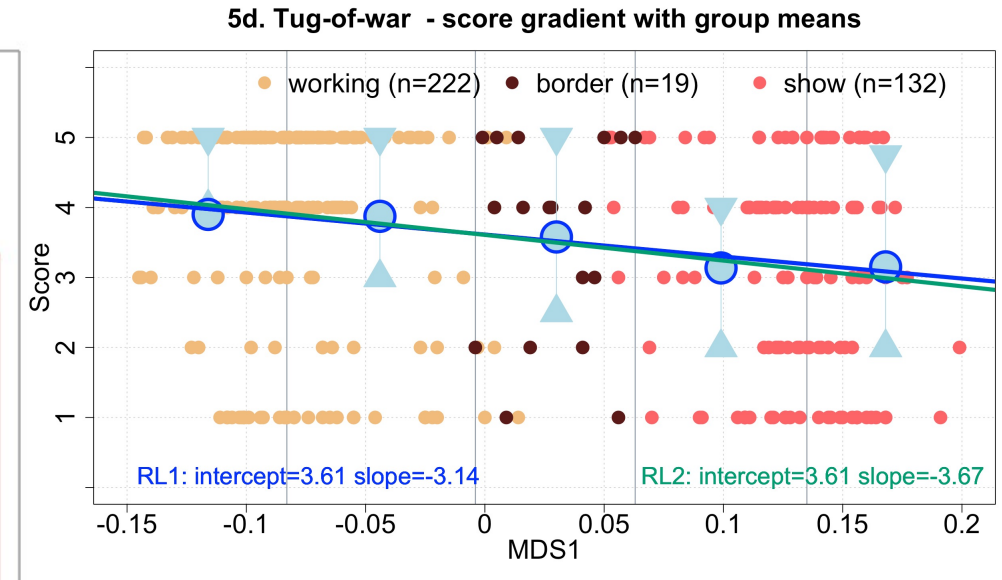

GWAS ENTIRE COHORT

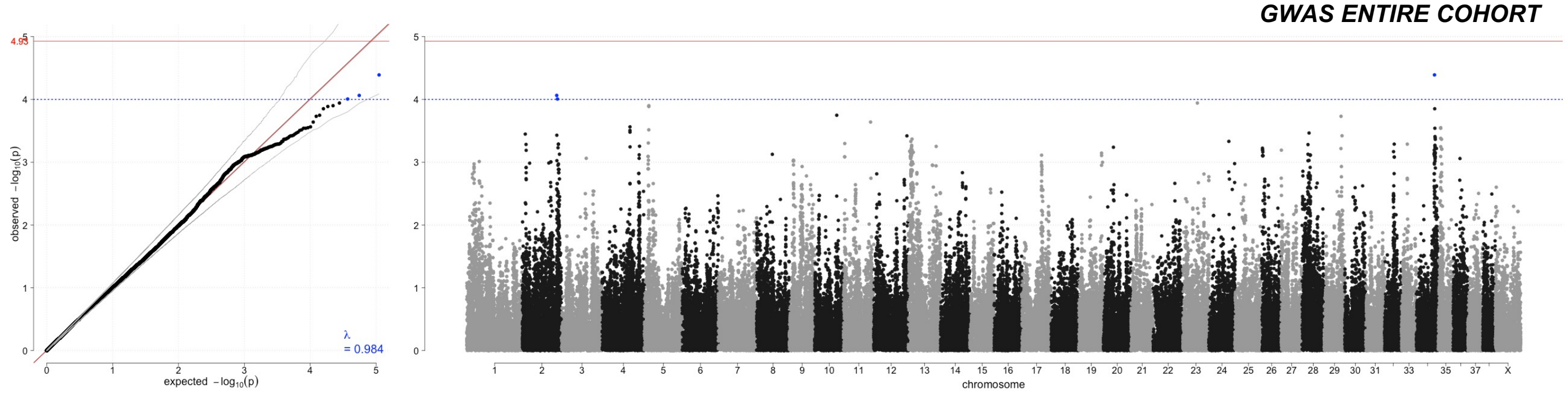

GWAS WORKING

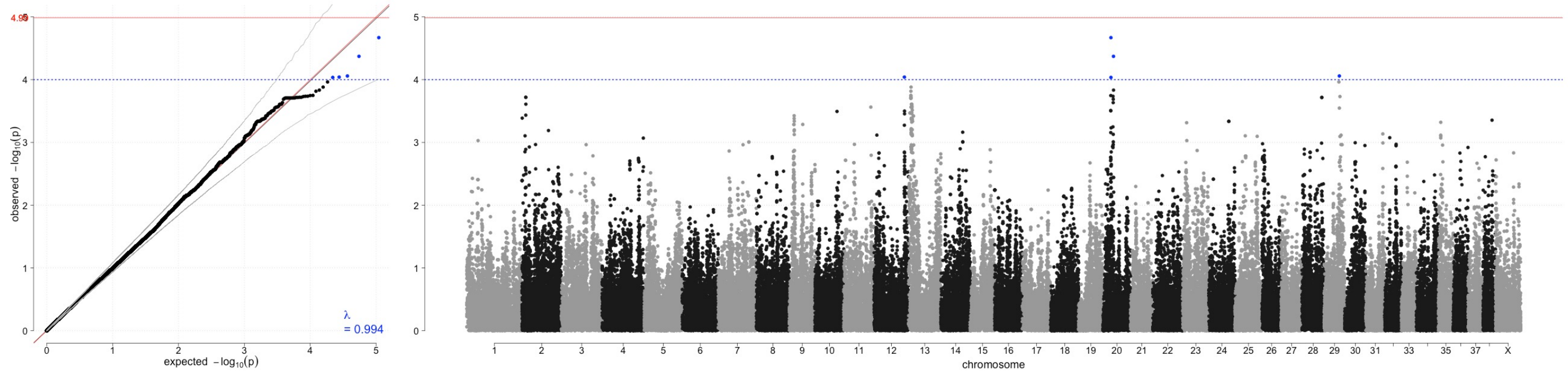

GWAS SHOW

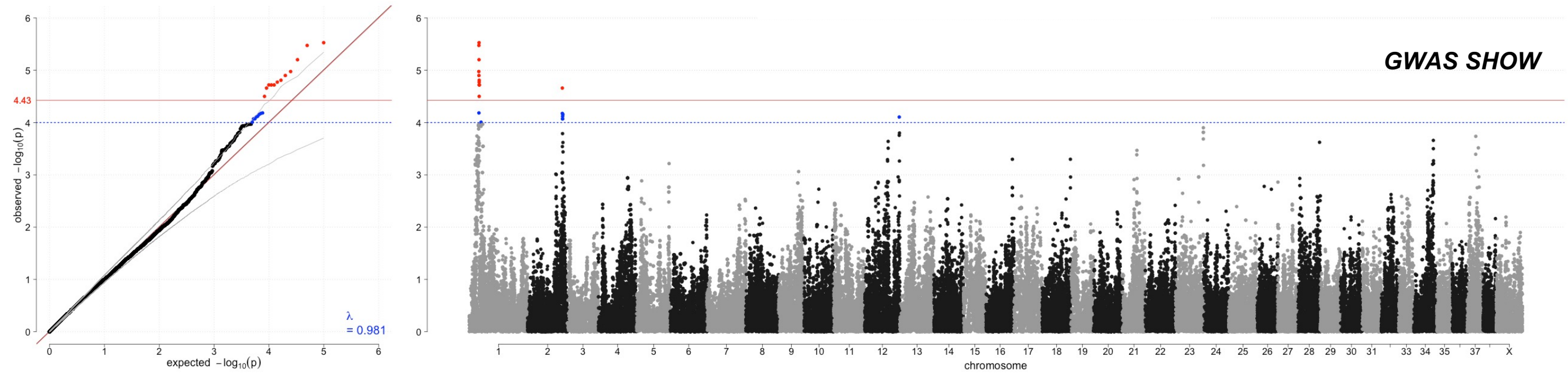

FOR GWAS RESULTS OBTAINED FOR THE WORKING AND SHOW SUB-POPULATIONS:

META-ANALYSIS FIXED EFFECTS

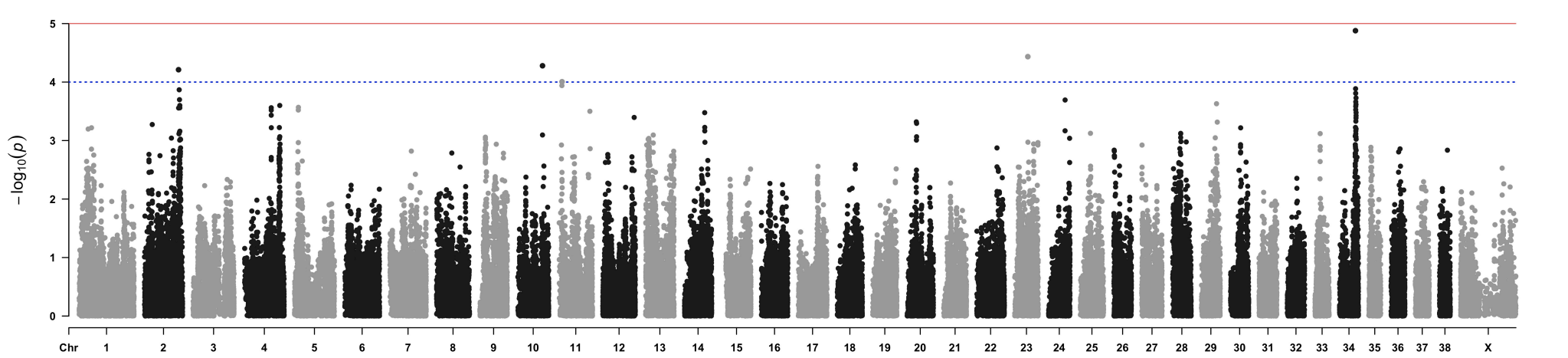

META-ANALYSIS RANDOM EFFECTS

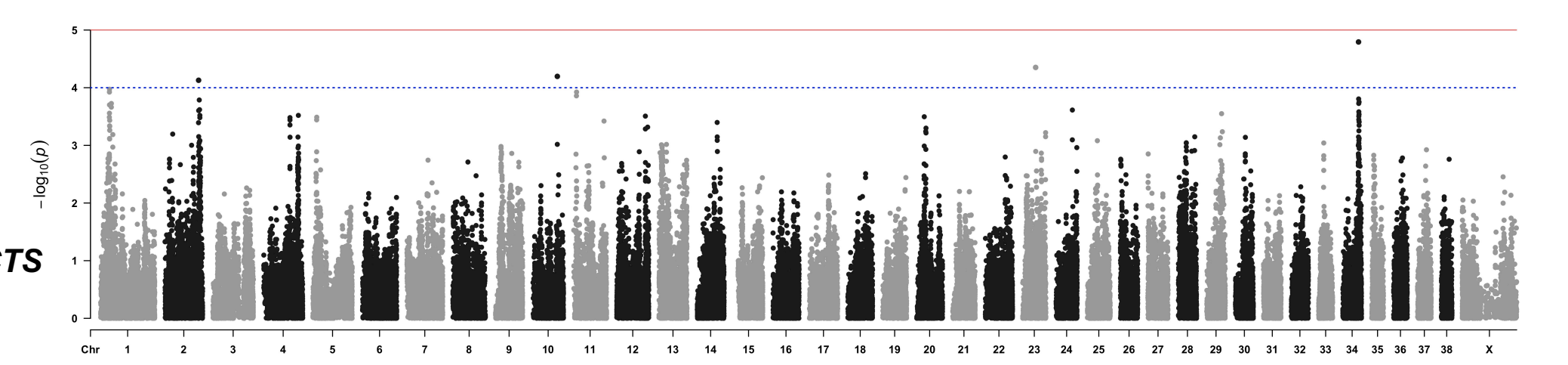

Trait: PLAYFULNESS

SCORE DISTRIBUTIONS

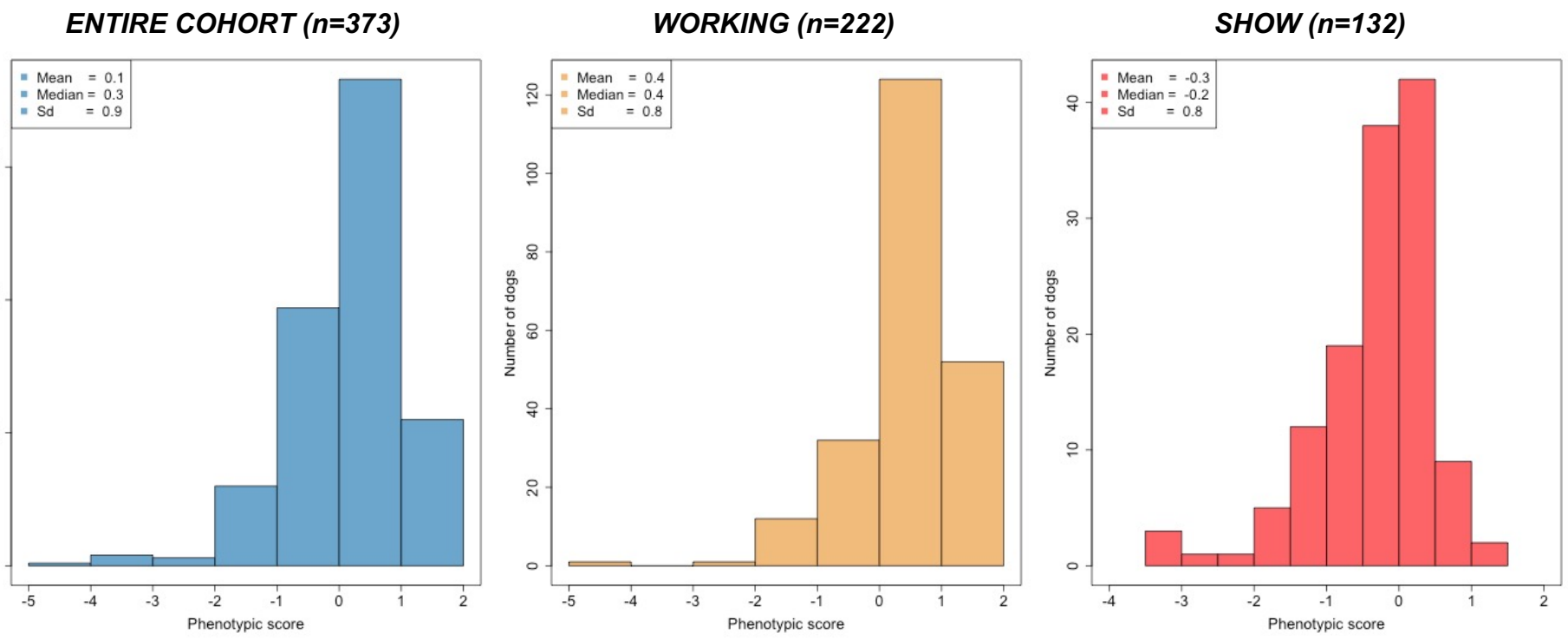

SCORE GRADIENT

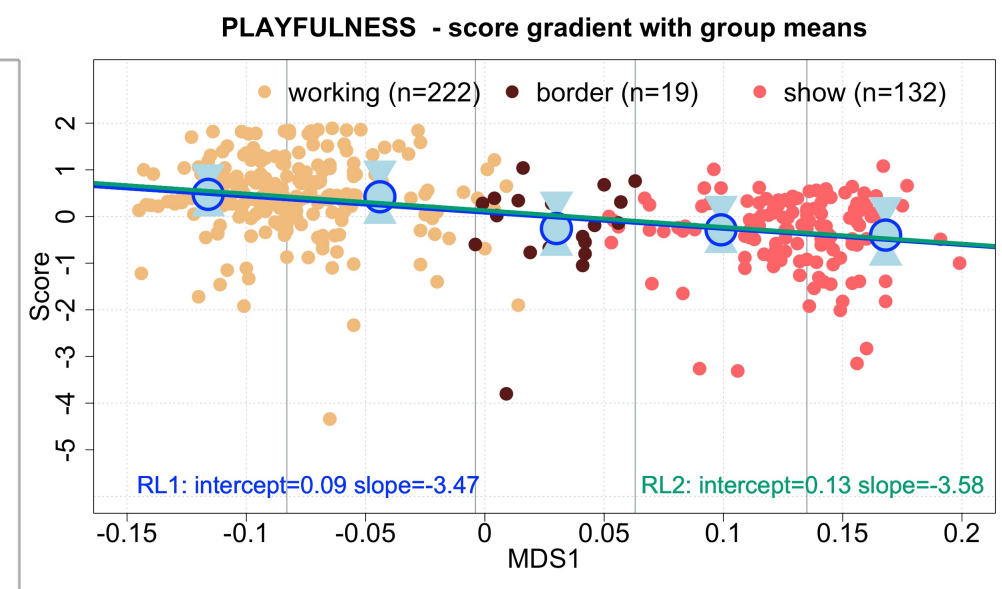

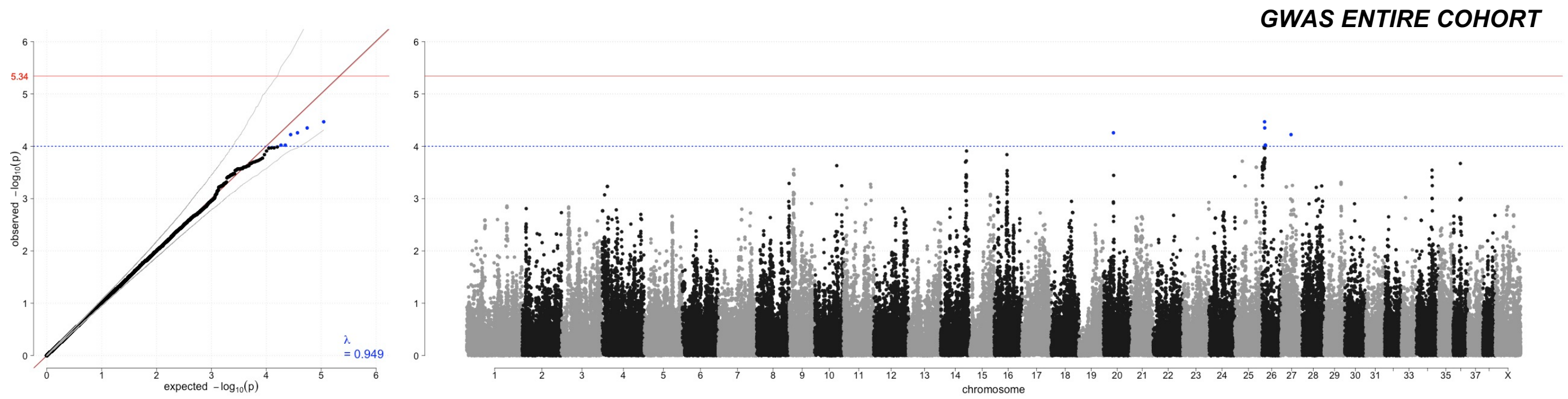

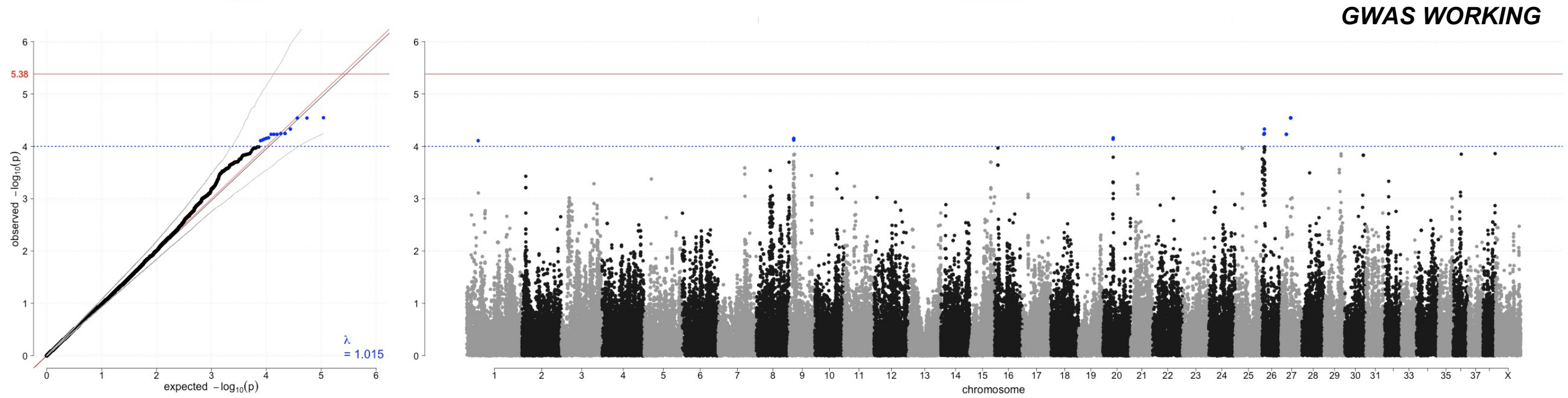

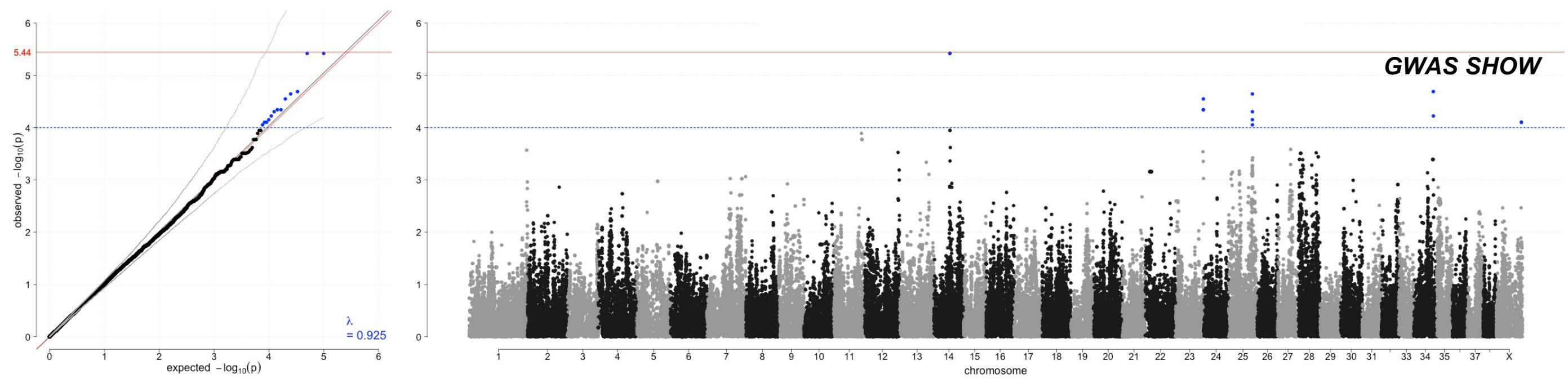

FOR GWAS RESULTS OBTAINED FOR THE WORKING AND SHOW SUB-POPULATIONS:

META-ANALYSIS FIXED EFFECTS

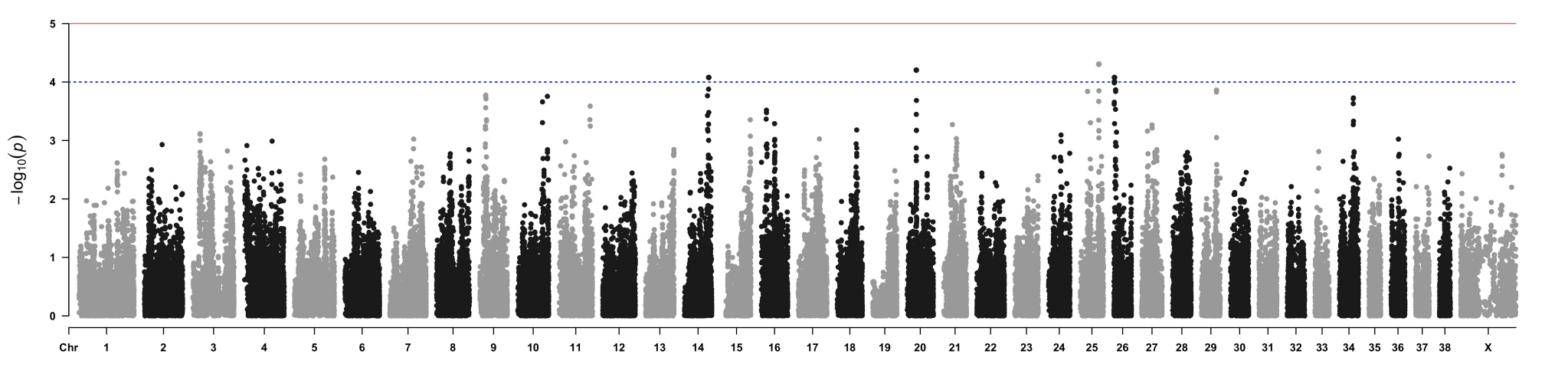

META-ANALYSIS RANDOM EFFECTS

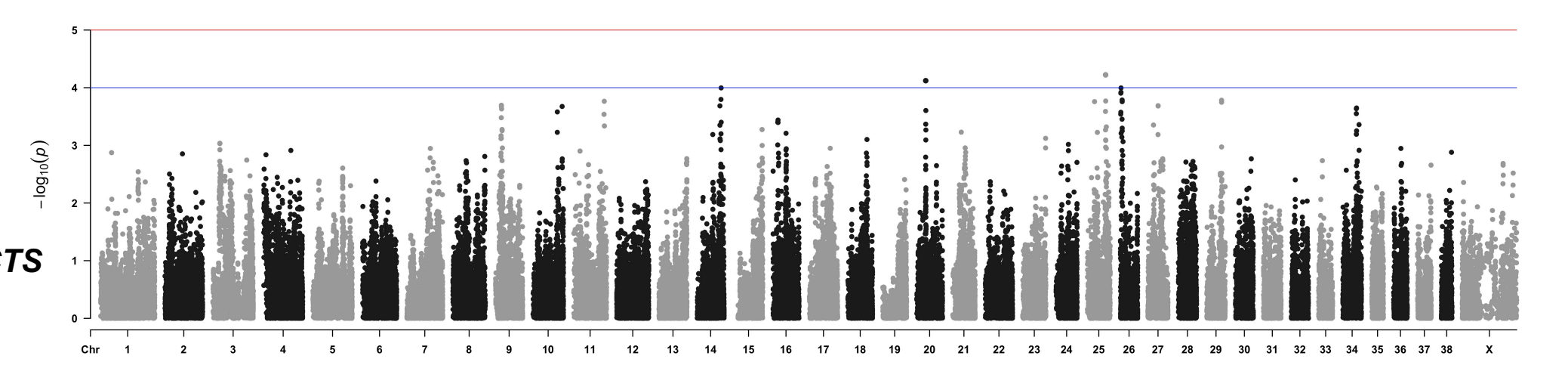

Behavioral variable: 2a. Interest in play

SCORE DISTRIBUTIONS

ENTIRE COHORT (n=373)

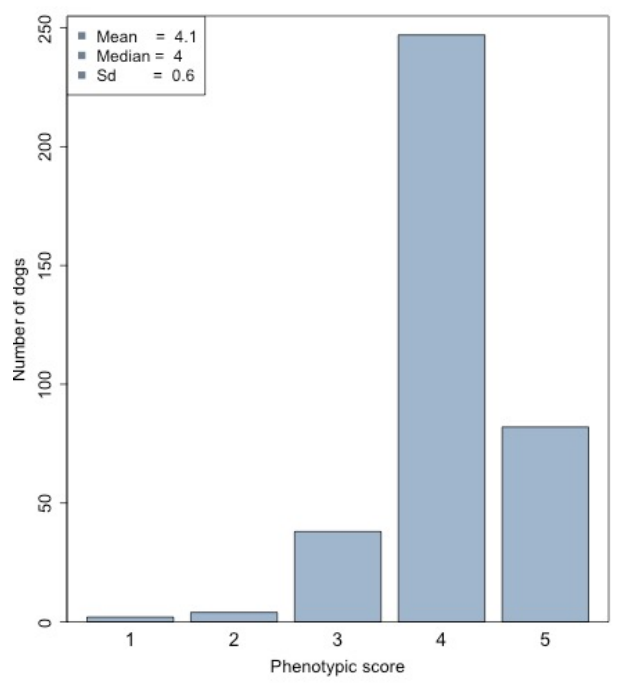

WORKING (n=222)

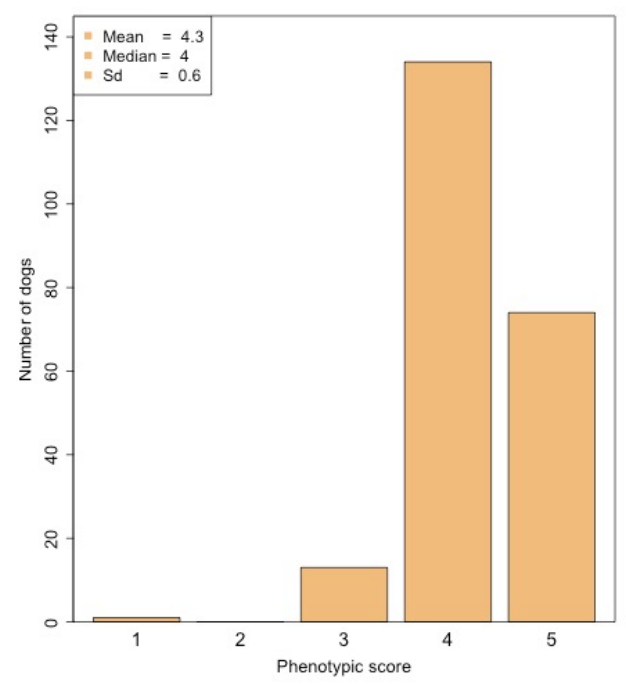

SHOW (n=132)

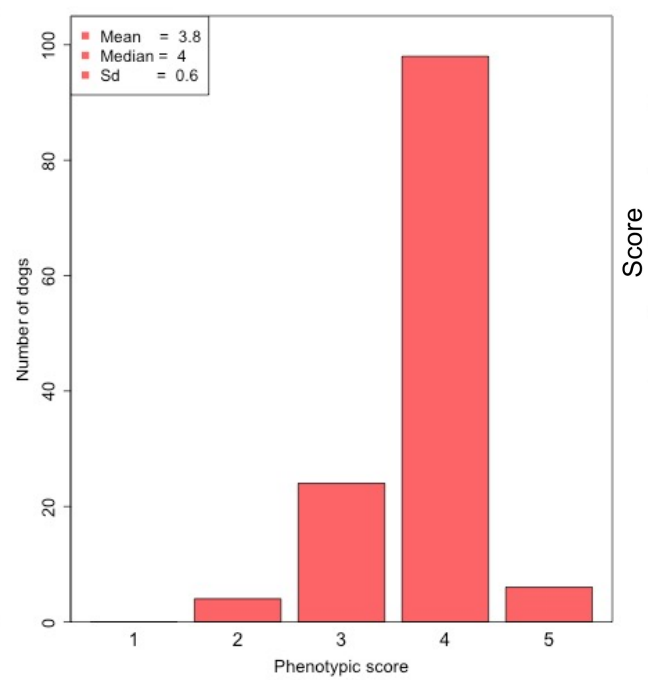

SCORE GRADIENT

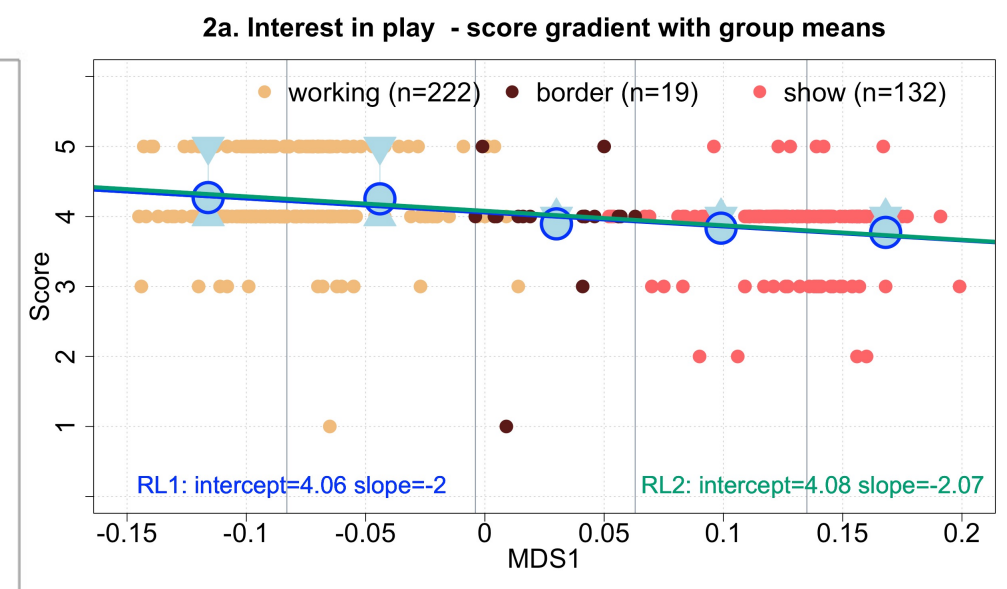

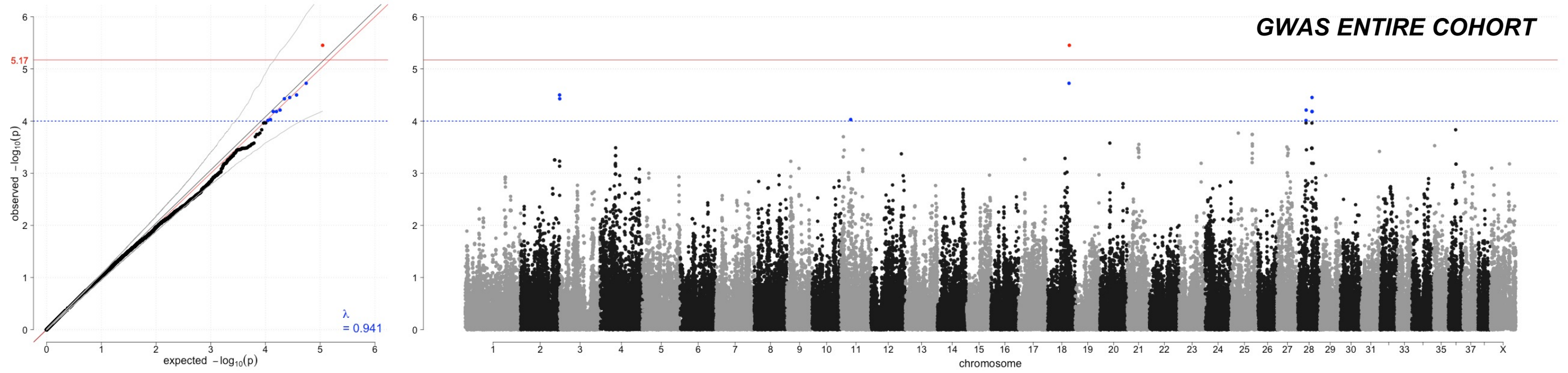

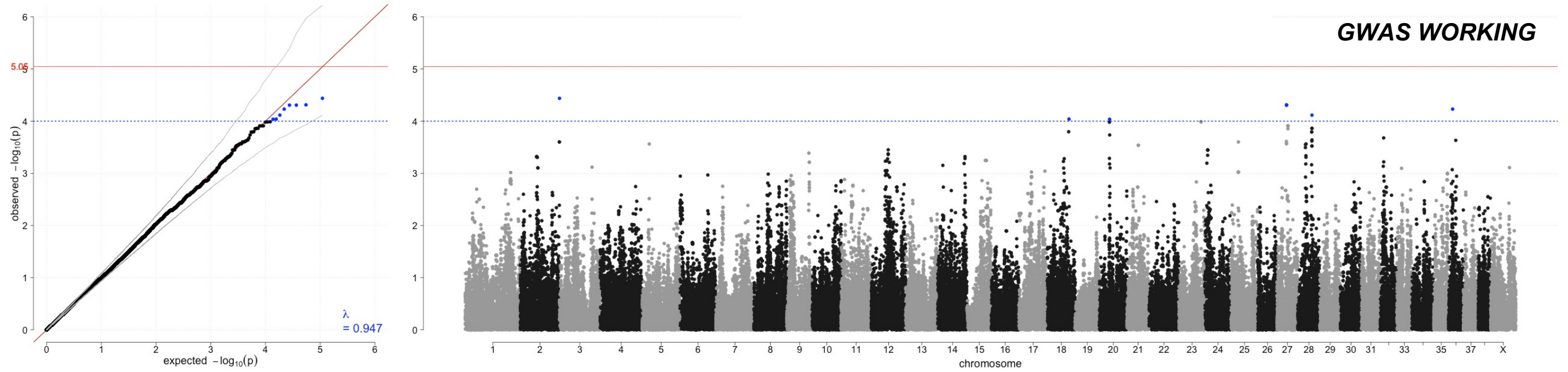

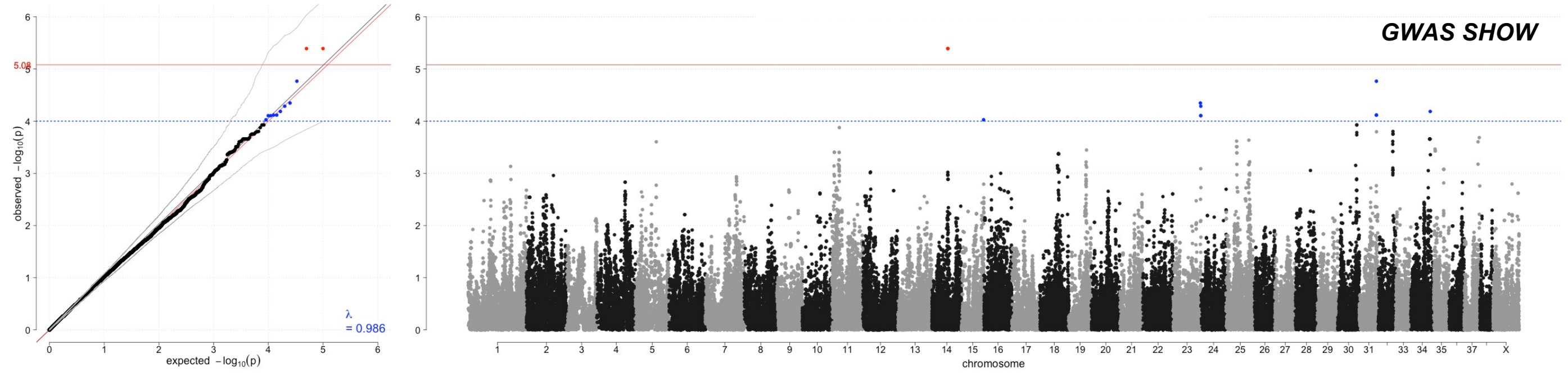

FOR GWAS RESULTS OBTAINED FOR THE WORKING AND SHOW SUB-POPULATIONS:

META-ANALYSIS FIXED EFFECTS

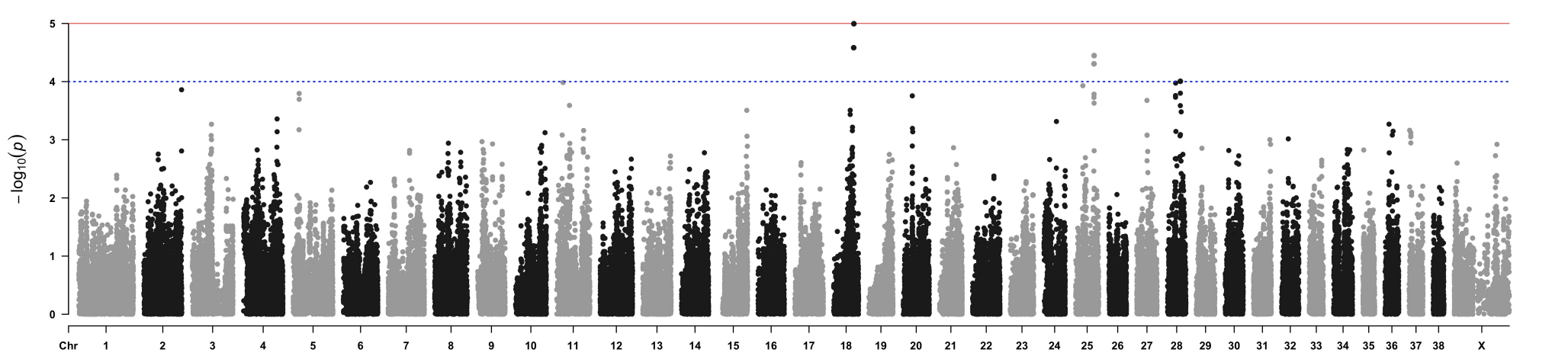

META-ANALYSIS RANDOM EFFECTS

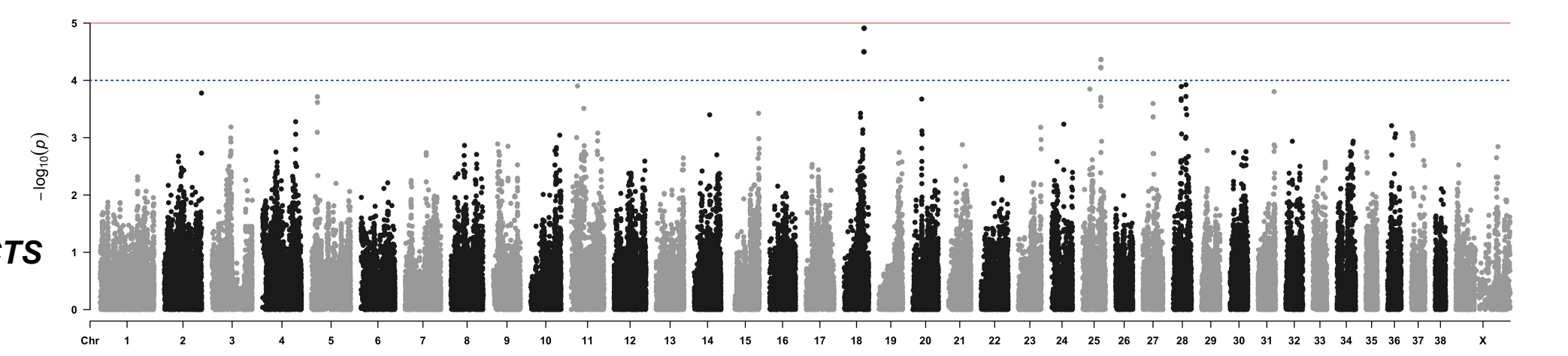

Behavioral variable: 2b. Grabbing

SCORE DISTRIBUTIONS

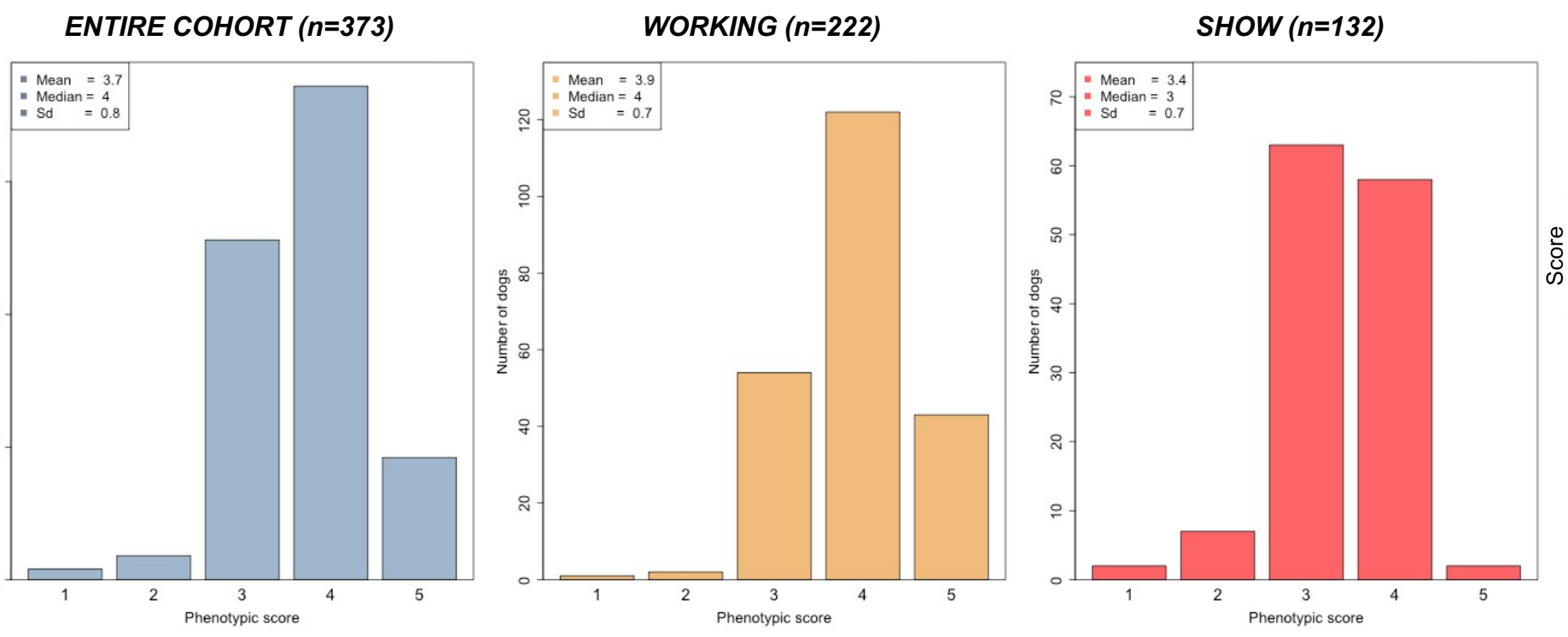

SCORE GRADIENT

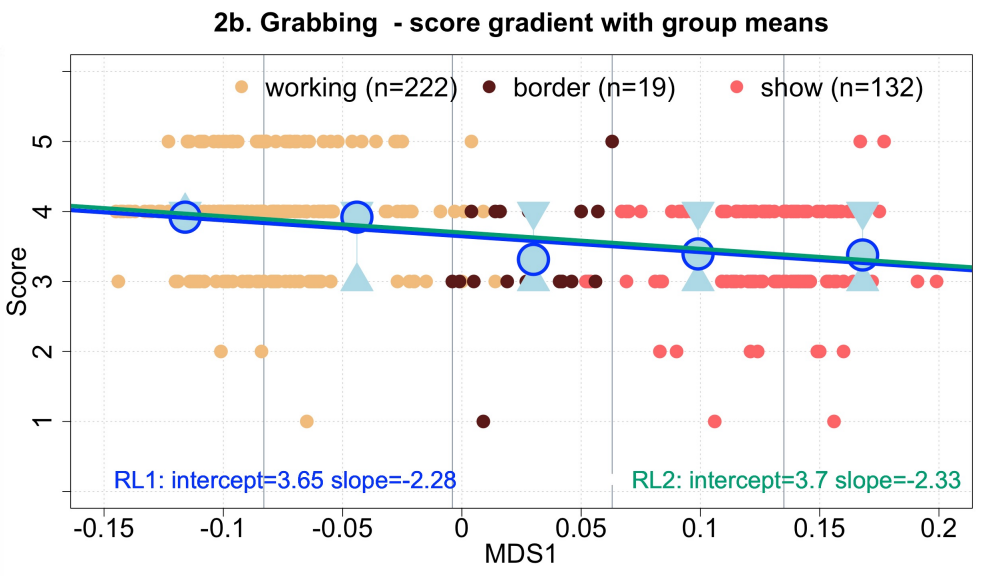

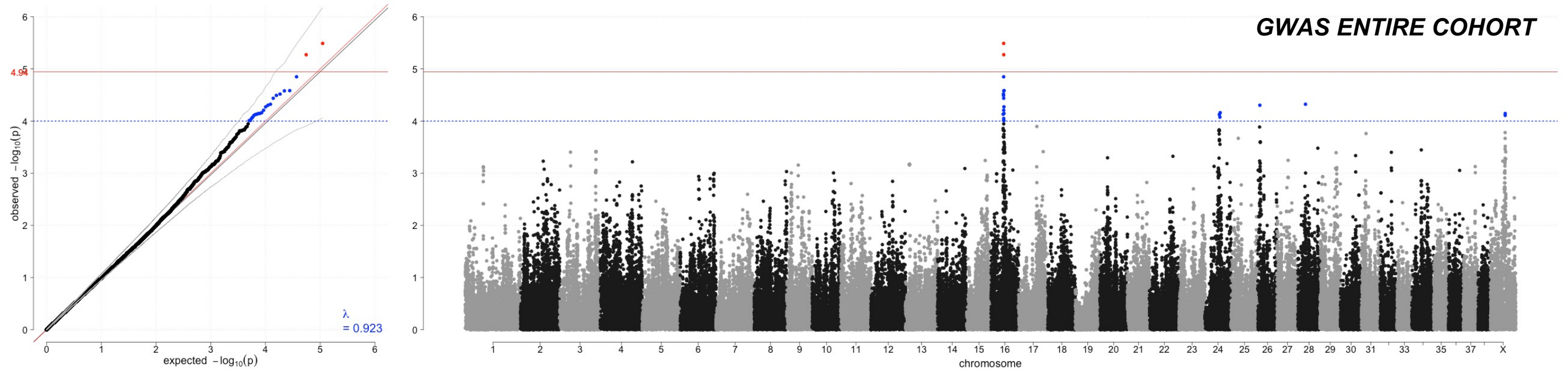

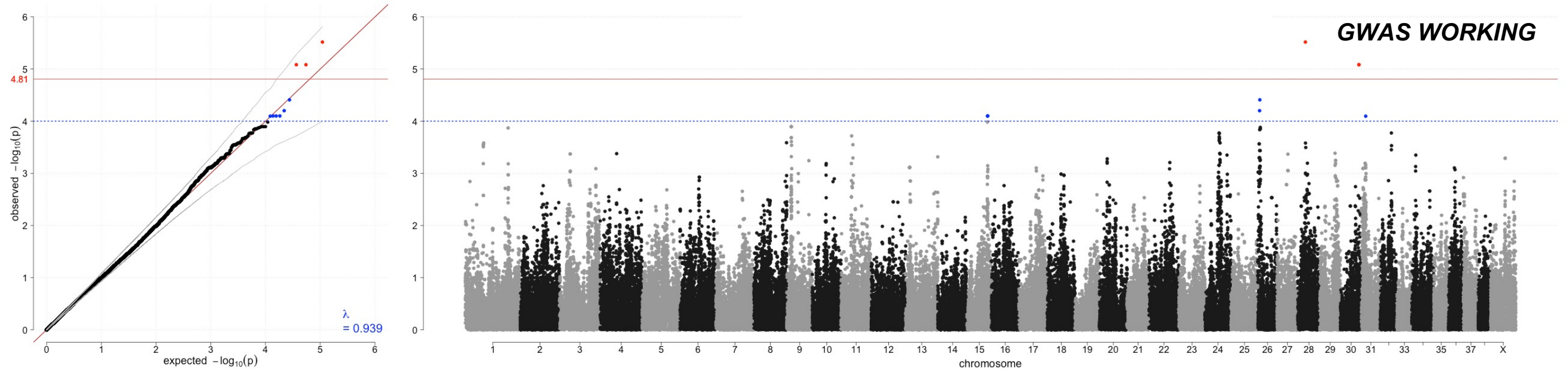

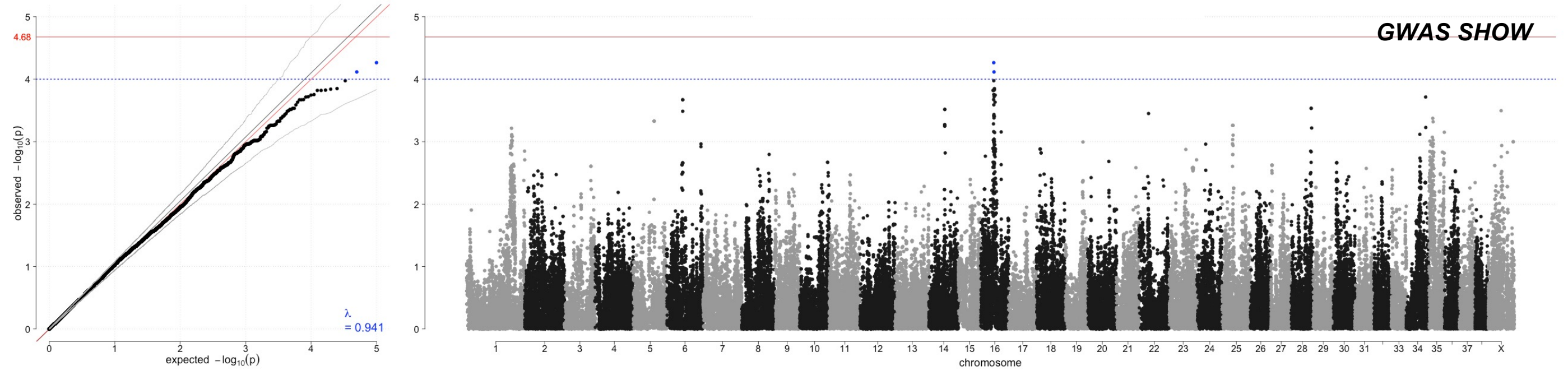

FOR GWAS RESULTS OBTAINED FOR THE WORKING AND SHOW SUB-POPULATIONS:

META-ANALYSIS FIXED EFFECTS

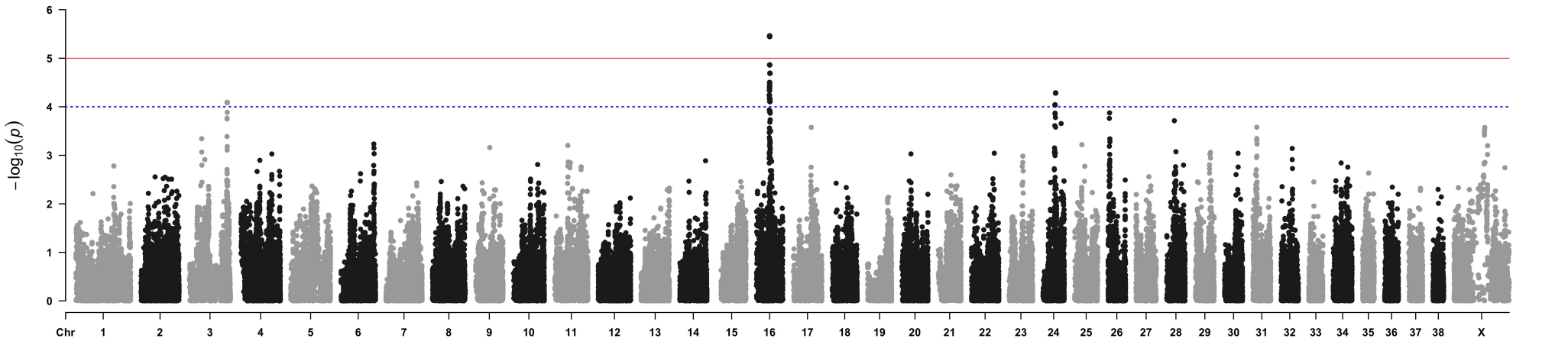

META-ANALYSIS RANDOM EFFECTS

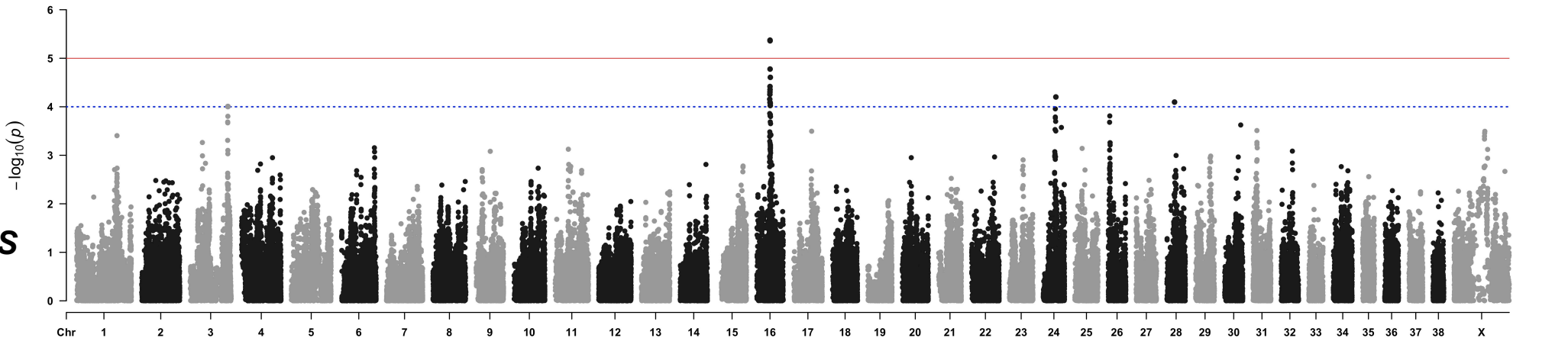

Behavioral variable: 2c. Tug-of-war

SCORE DISTRIBUTIONS

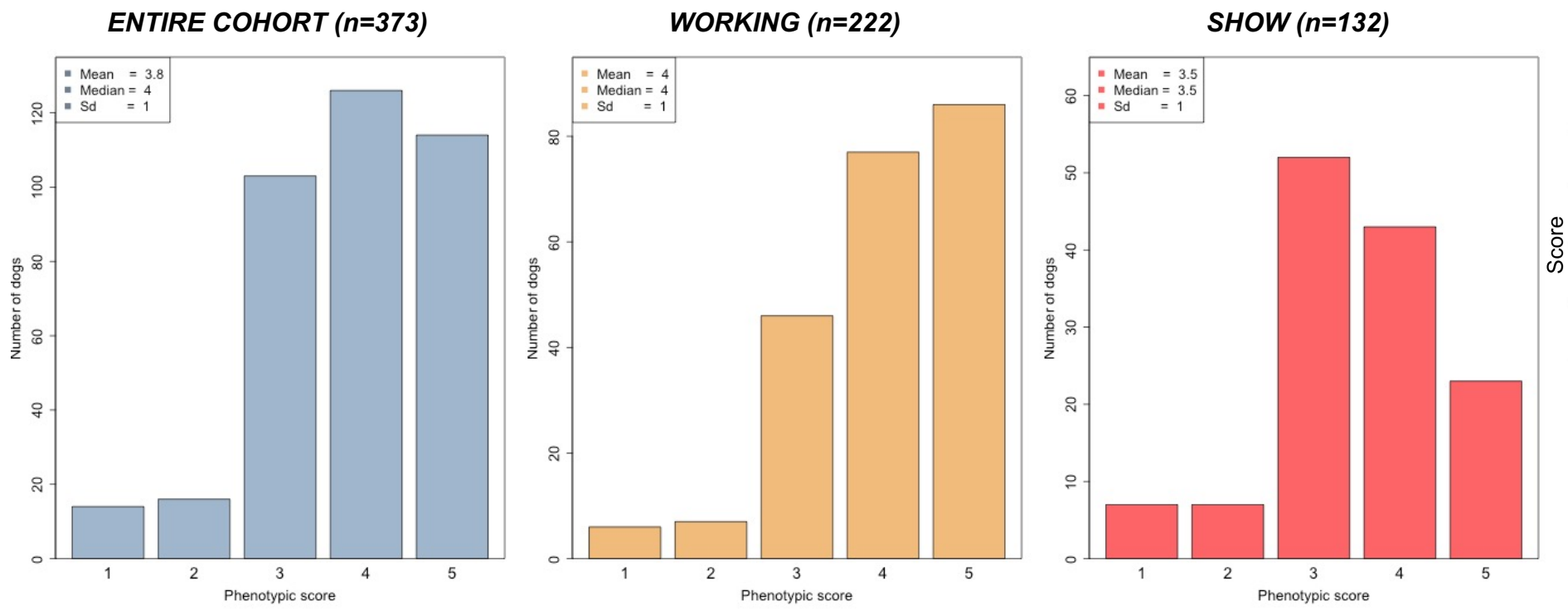

SCORE GRADIENT

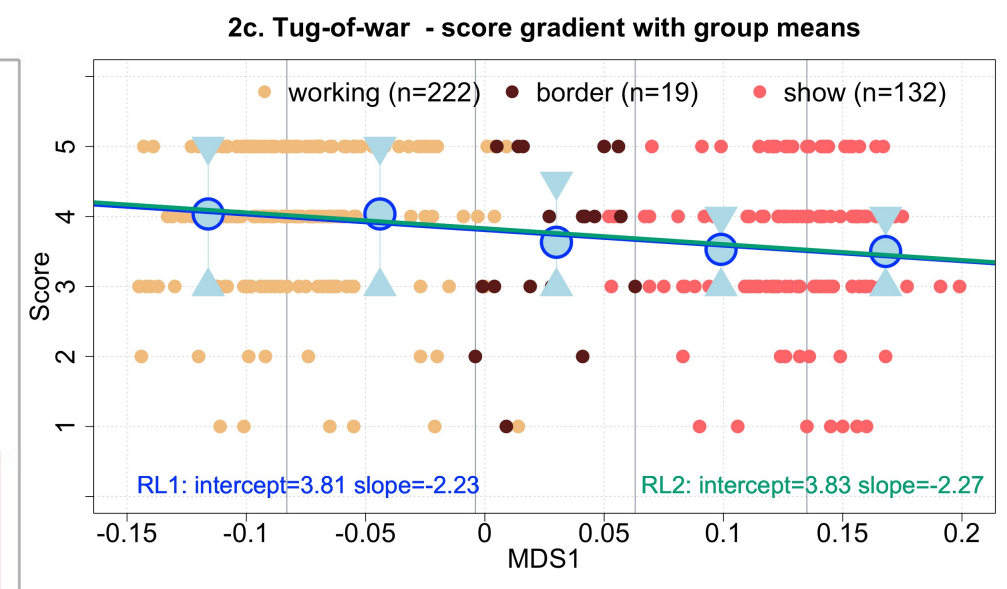

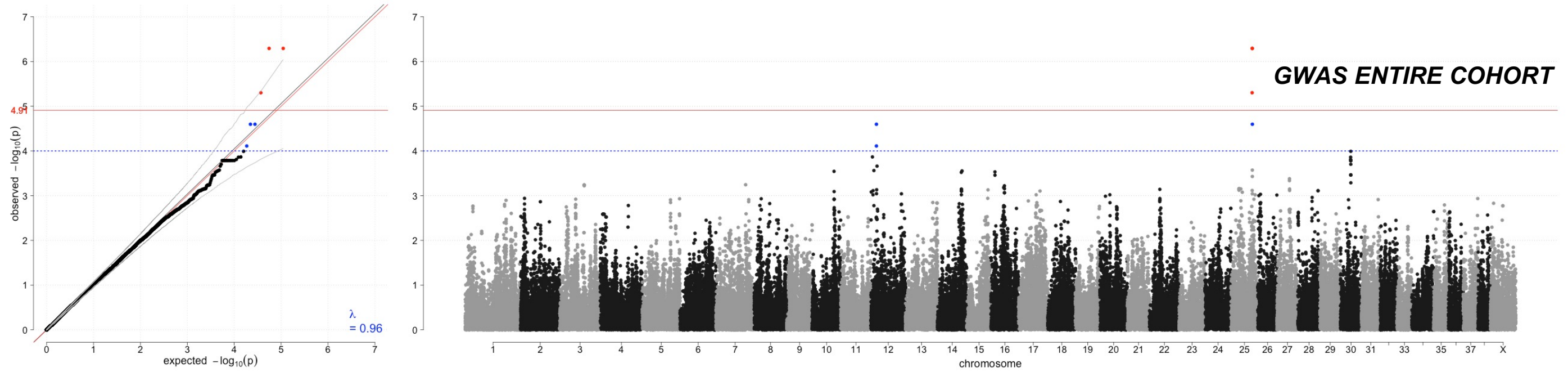

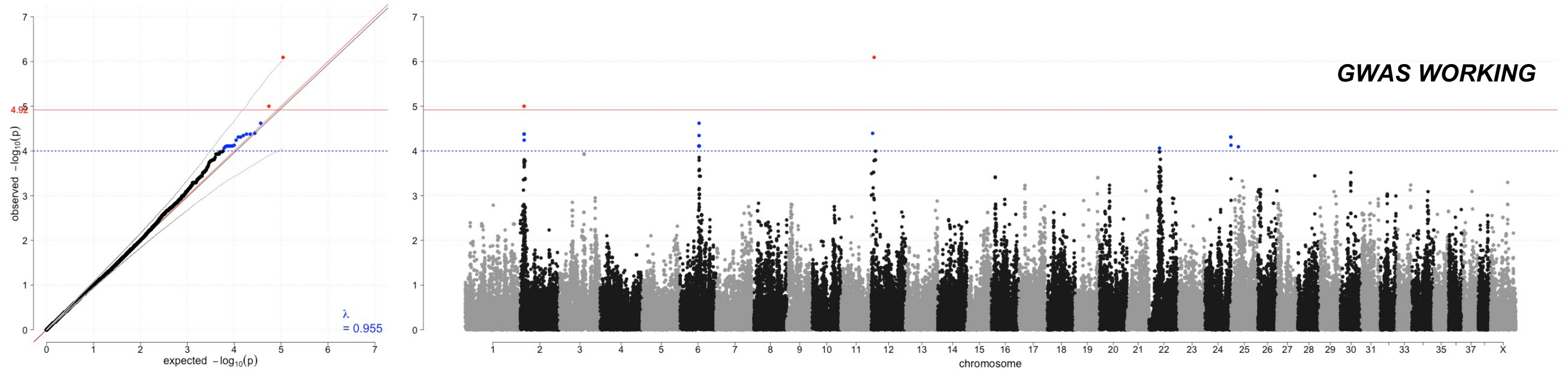

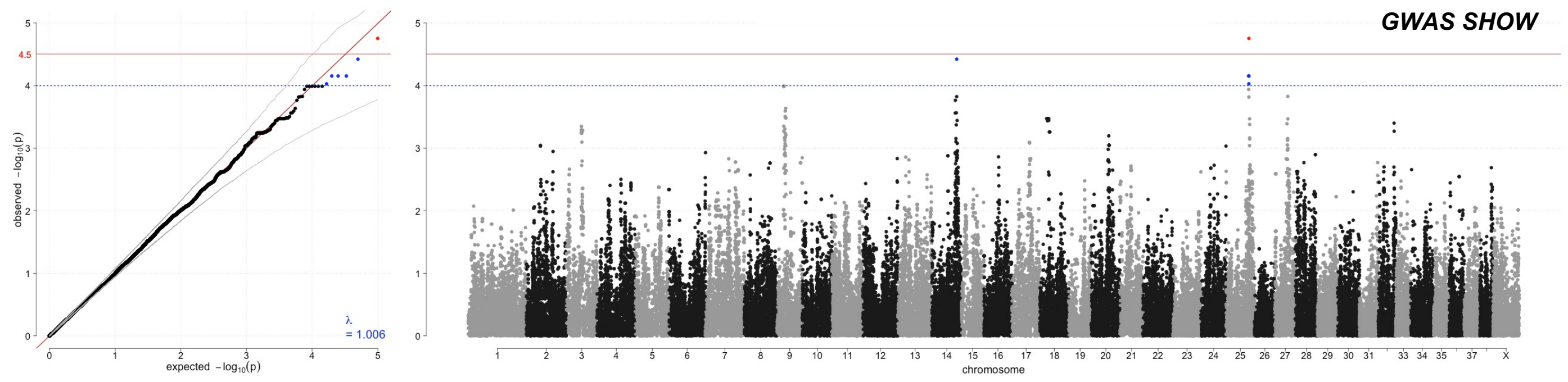

FOR GWAS RESULTS OBTAINED FOR THE WORKING AND SHOW SUB-POPULATIONS:

META-ANALYSIS FIXED EFFECTS

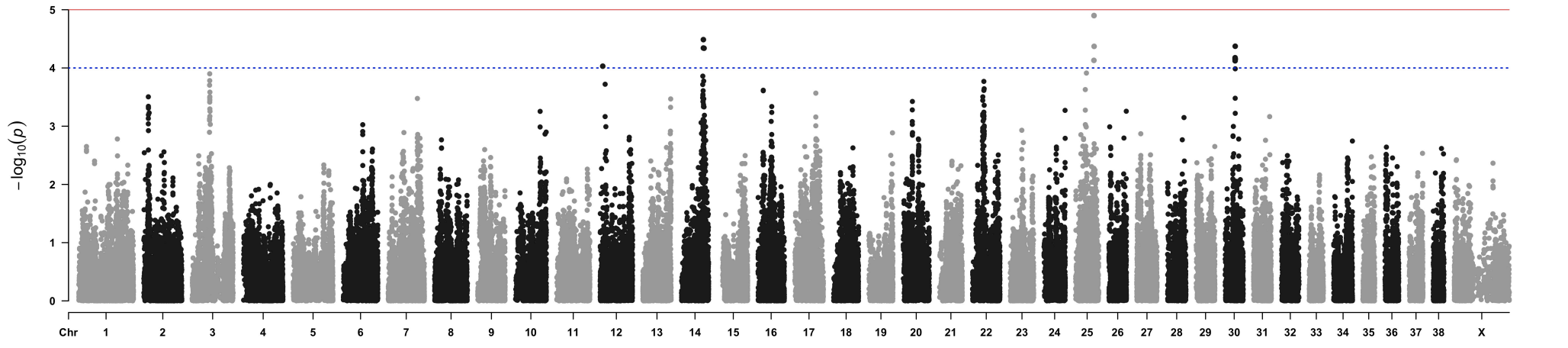

META-ANALYSIS RANDOM EFFECTS

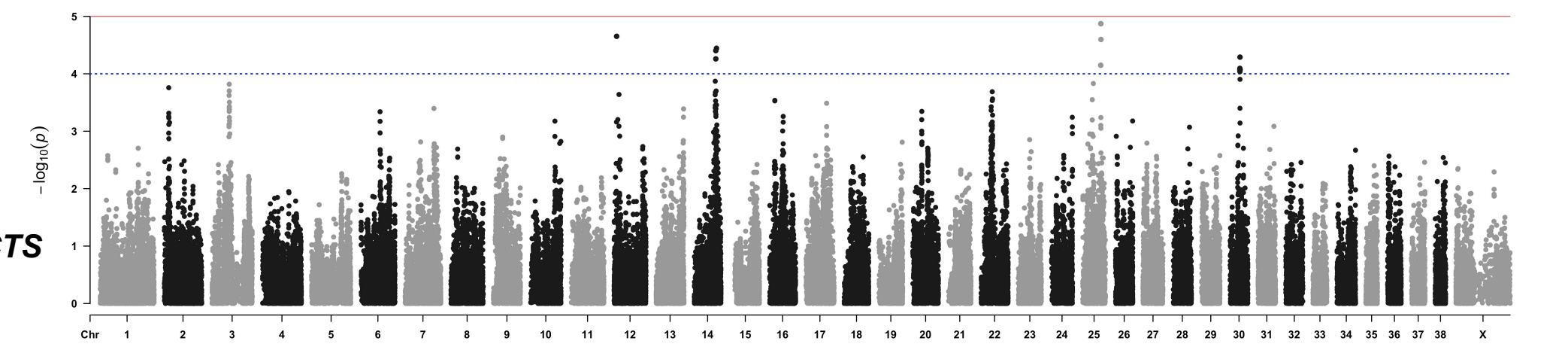

Behavioral variable: 9a. Interest in play

SCORE DISTRIBUTIONS

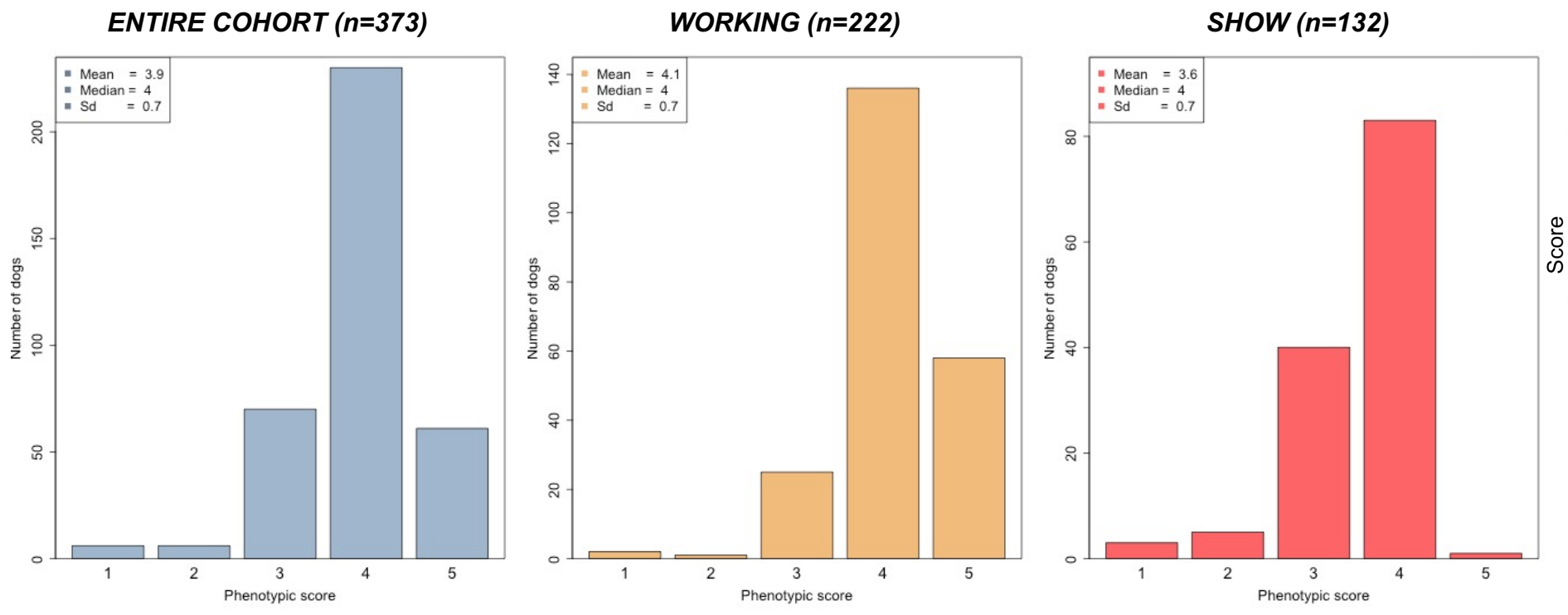

SCORE GRADIENT

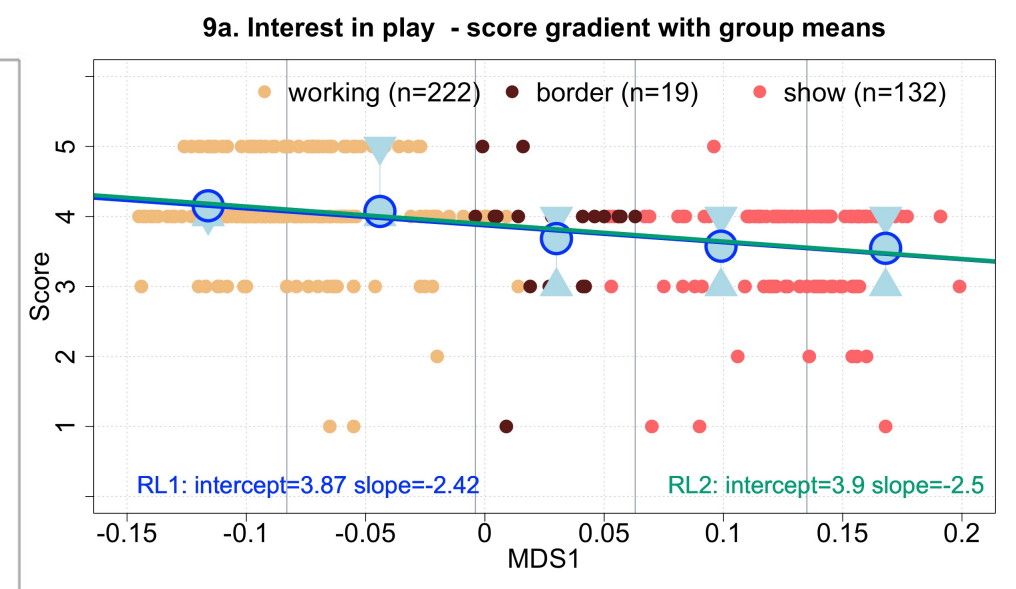

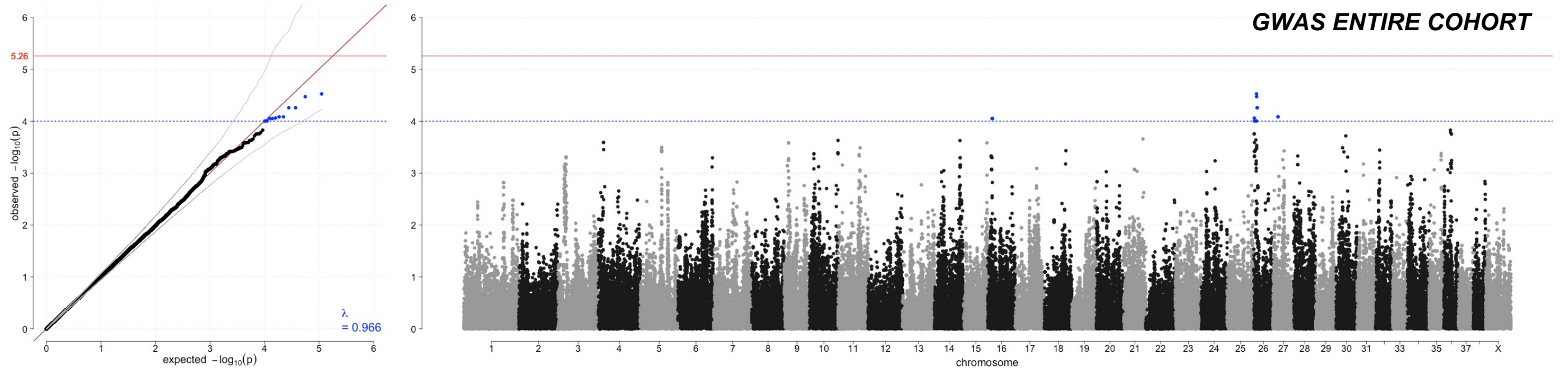

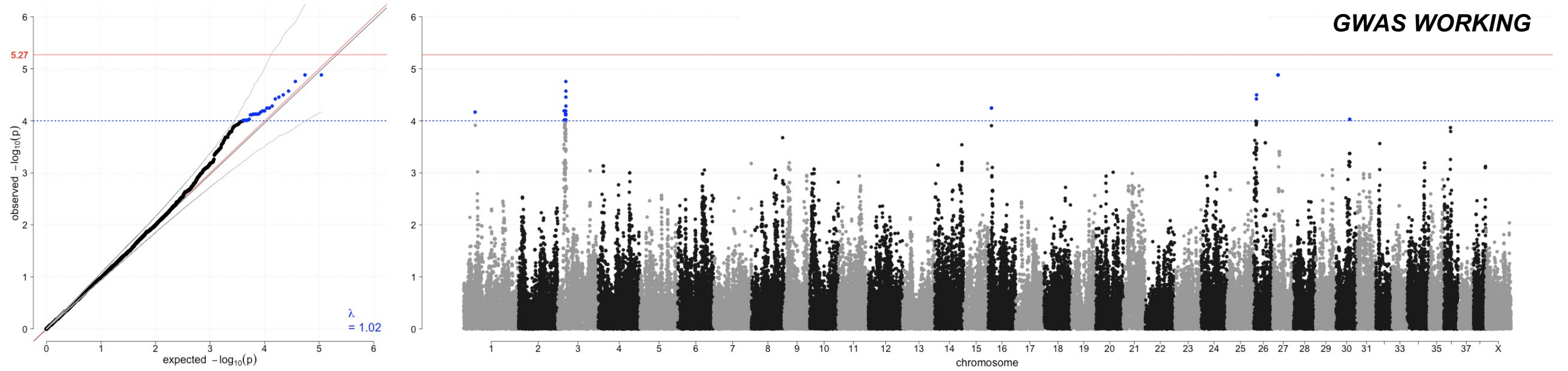

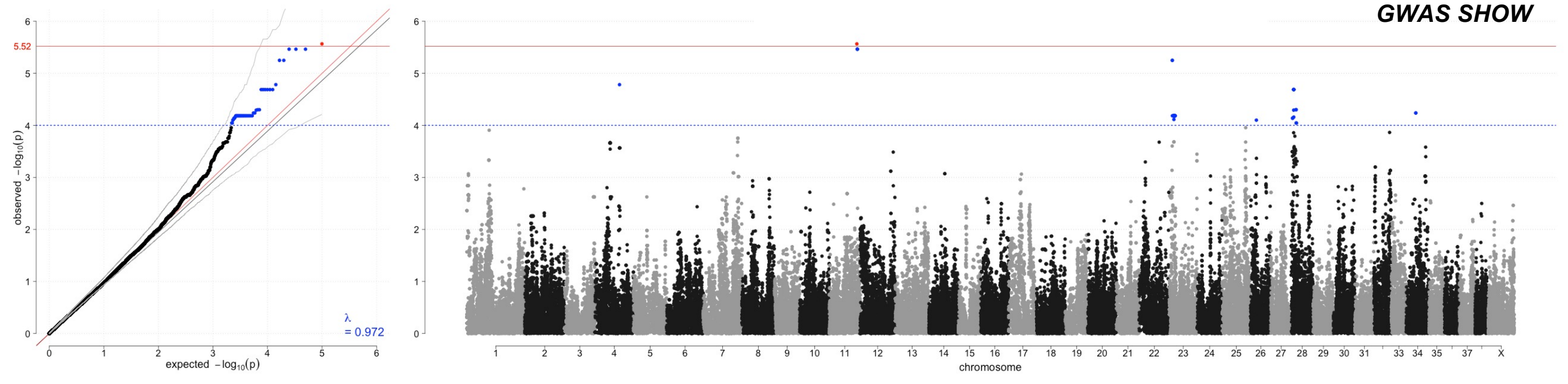

FOR GWAS RESULTS OBTAINED FOR THE WORKING AND SHOW SUB-POPULATIONS:

META-ANALYSIS FIXED EFFECTS

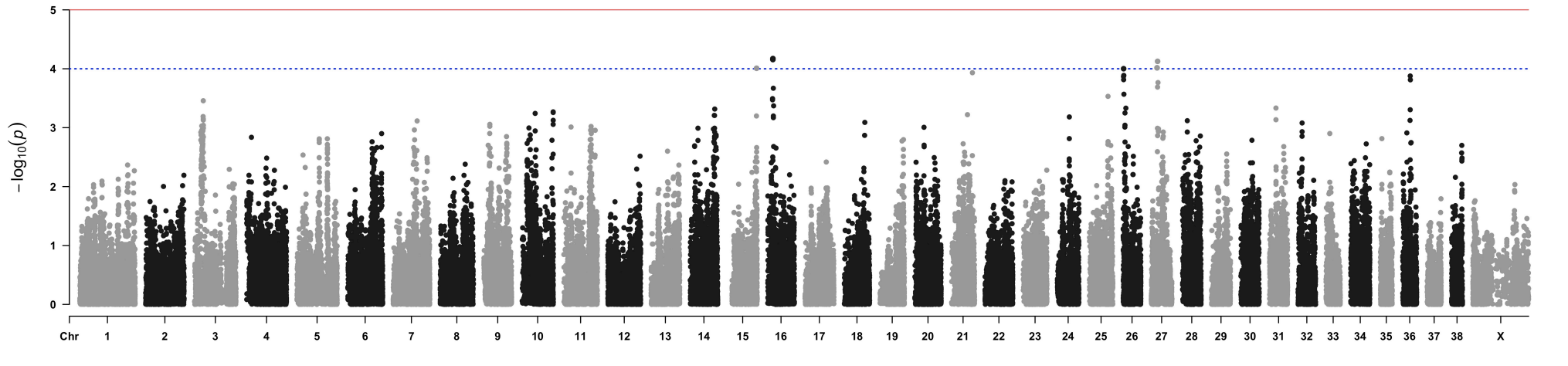

META-ANALYSIS RANDOM EFFECTS

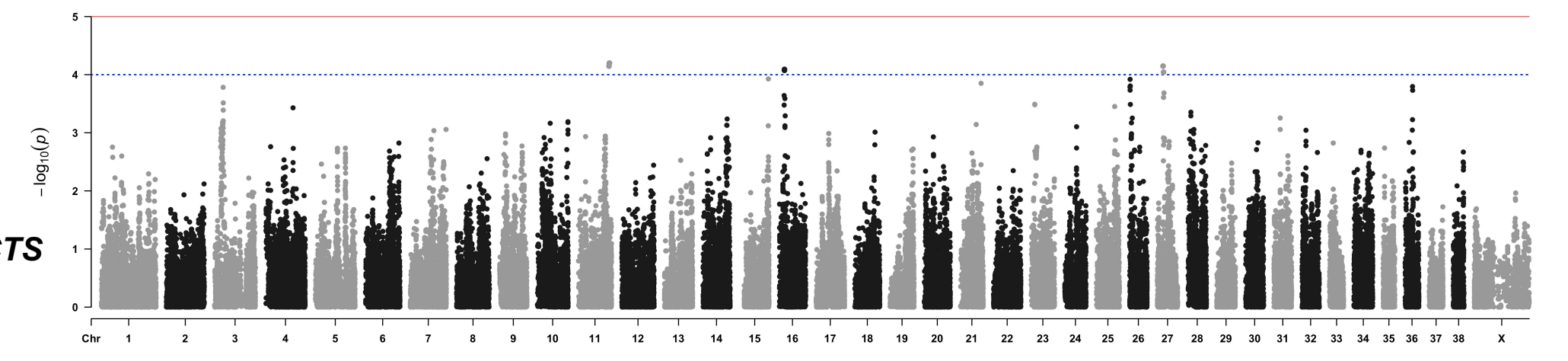

Behavioral variable: 9b. Grabbing

SCORE DISTRIBUTIONS

ENTIRE COHORT (n=373) — WORKING (n=222) — SHOW (n=132)

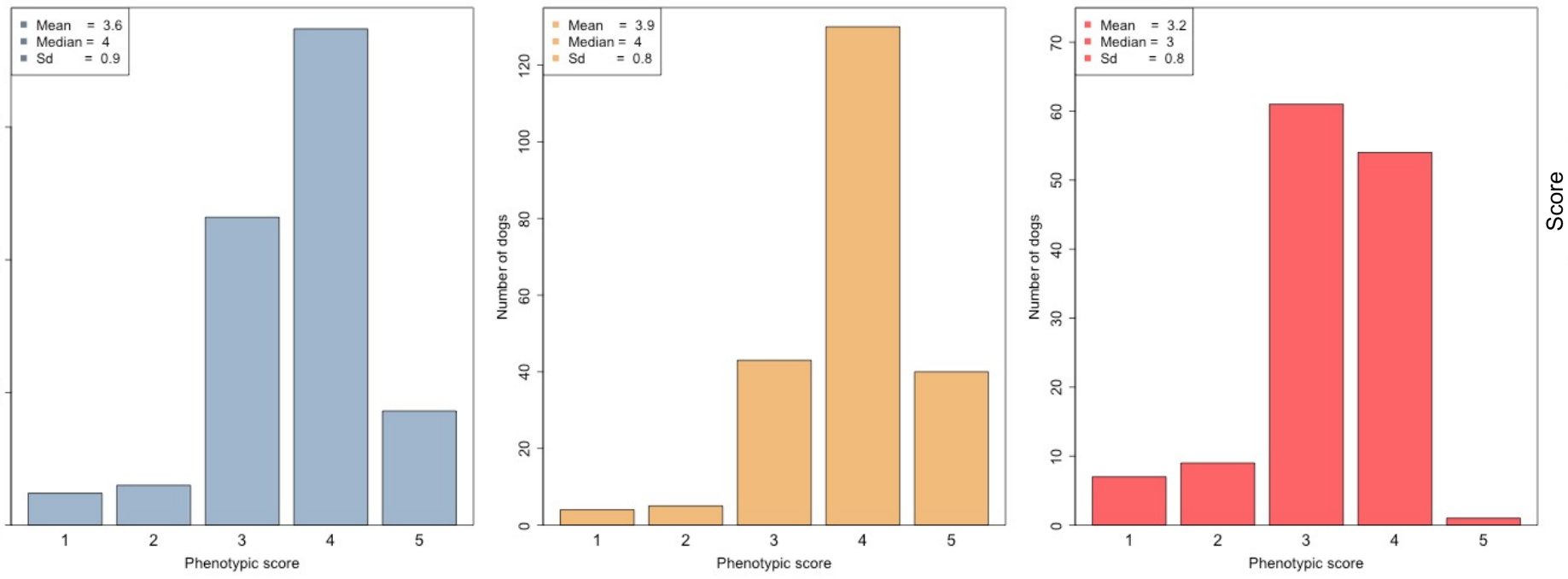

SCORE GRADIENT

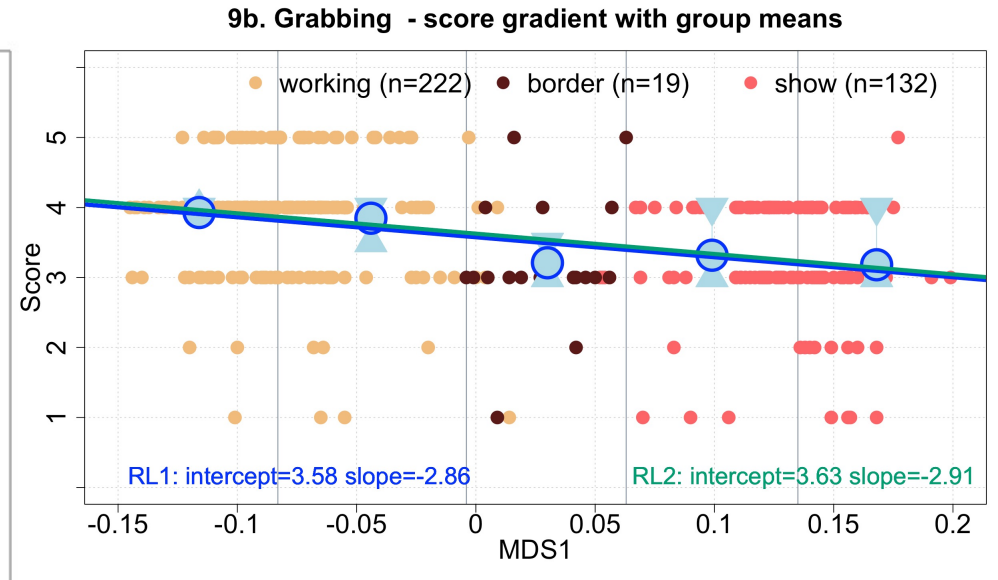

GWAS ENTIRE COHORT

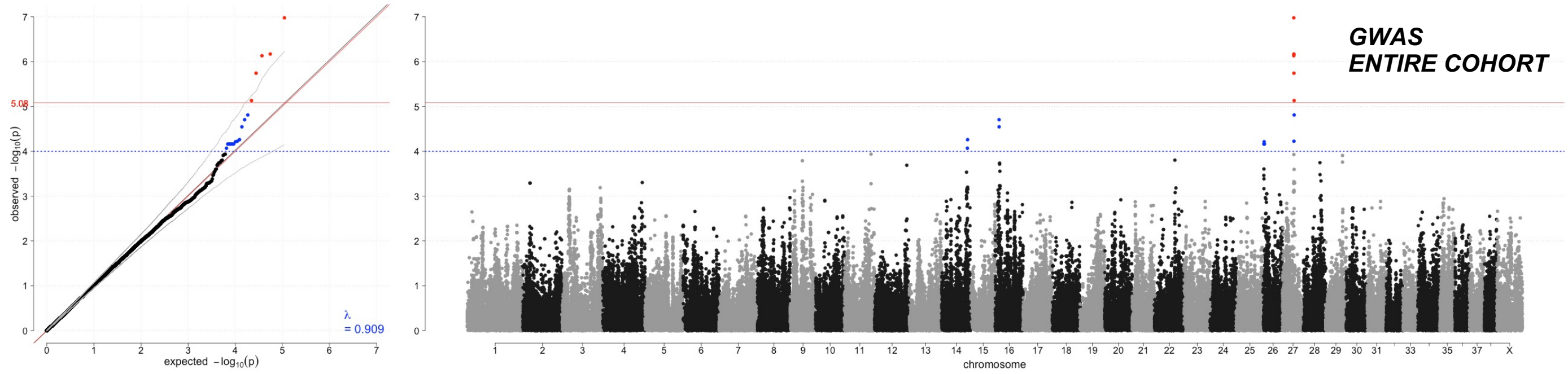

GWAS WORKING

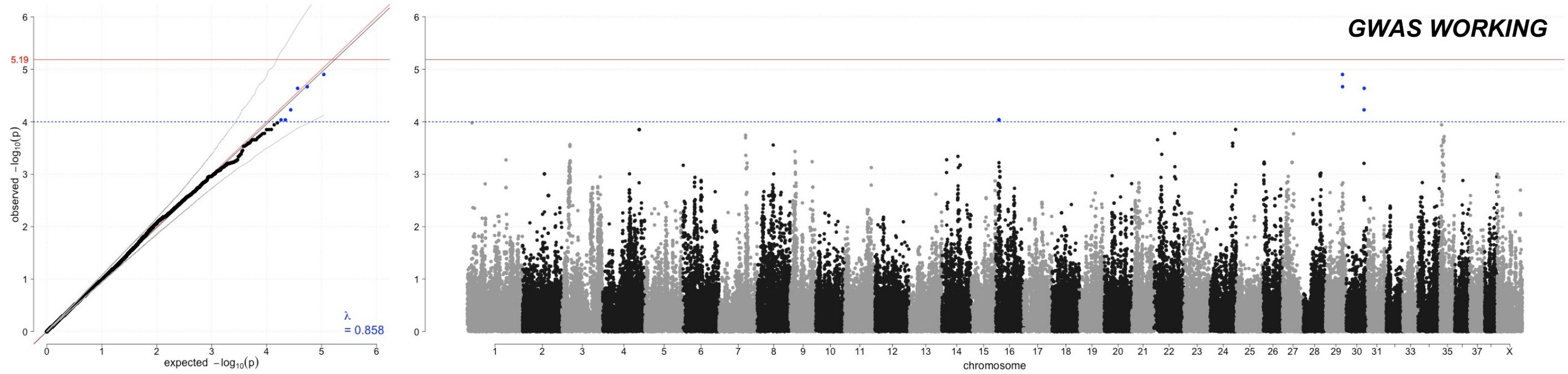

GWAS SHOW

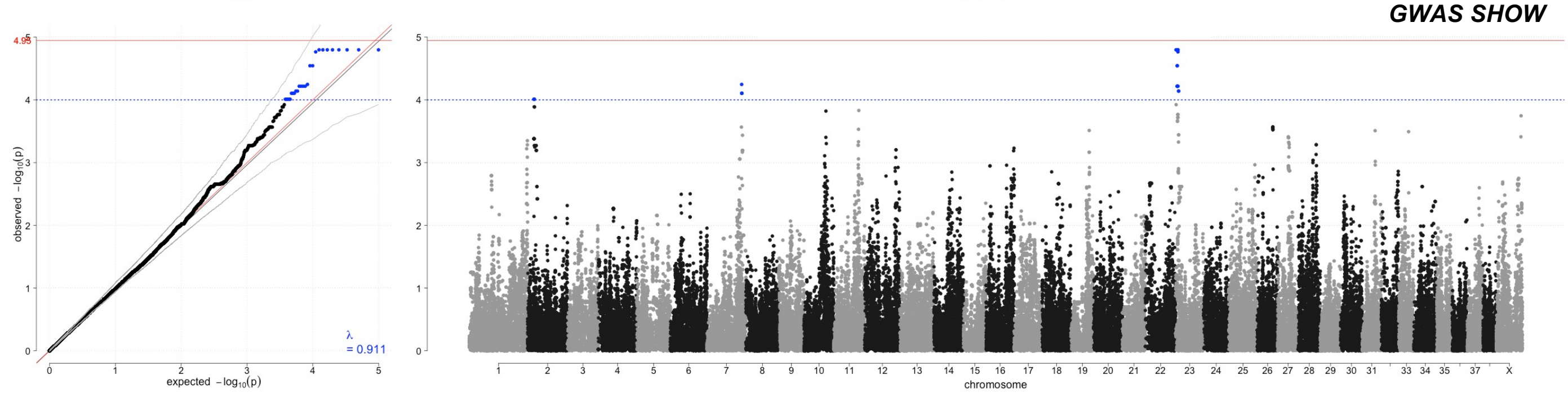

FOR GWAS RESULTS OBTAINED FOR THE WORKING AND SHOW SUB-POPULATIONS:

META-ANALYSIS FIXED EFFECTS

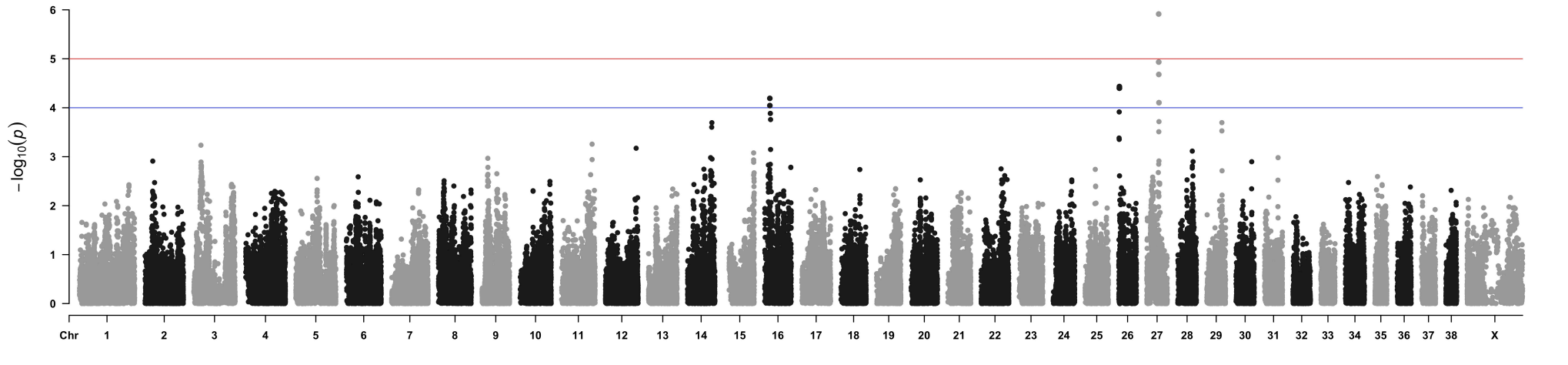

META-ANALYSIS RANDOM EFFECTS

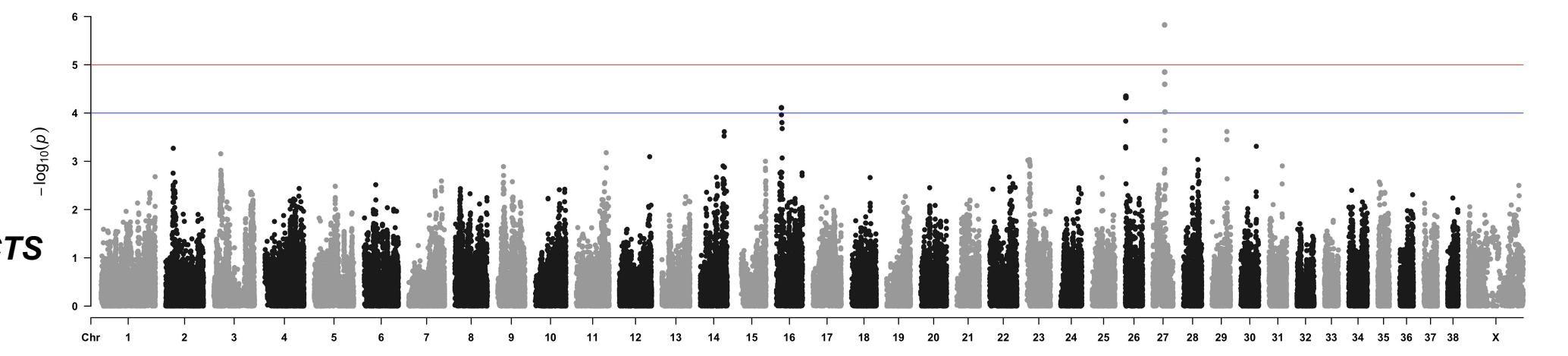

Behavioral variable: 3b. Grabbing 1

SCORE DISTRIBUTIONS

ENTIRE COHORT (n=373) | WORKING (n=222) | SHOW (n=132)

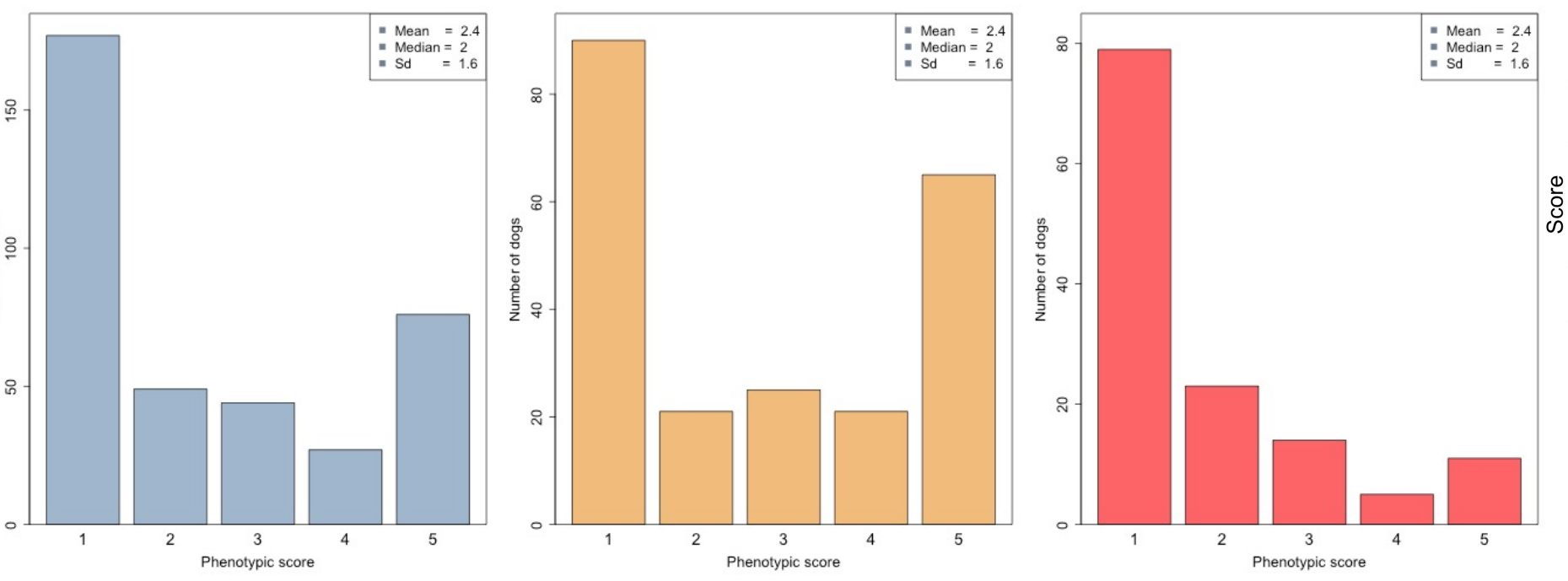

SCORE GRADIENT

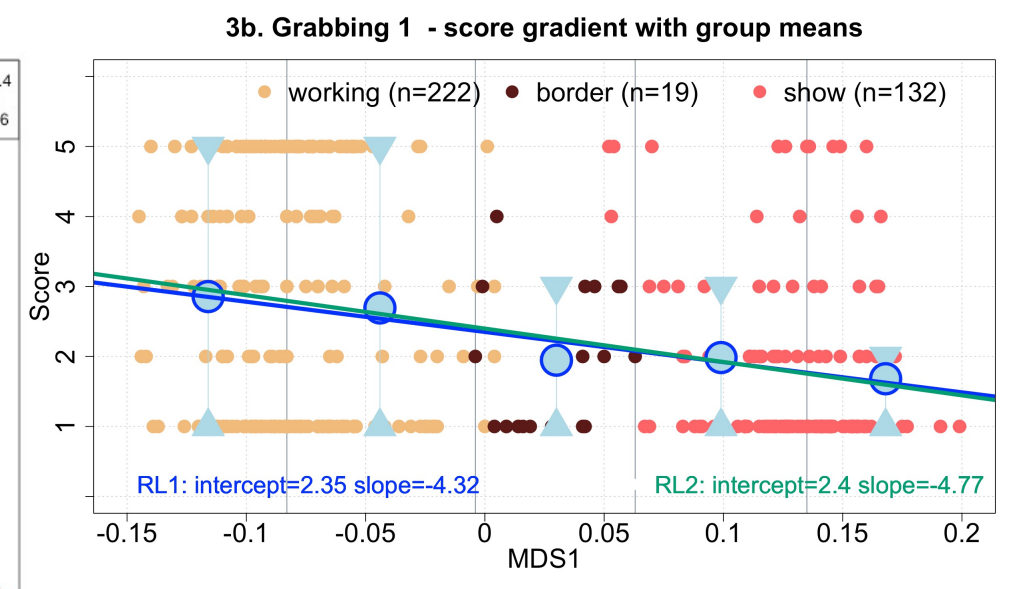

GWAS ENTIRE COHORT

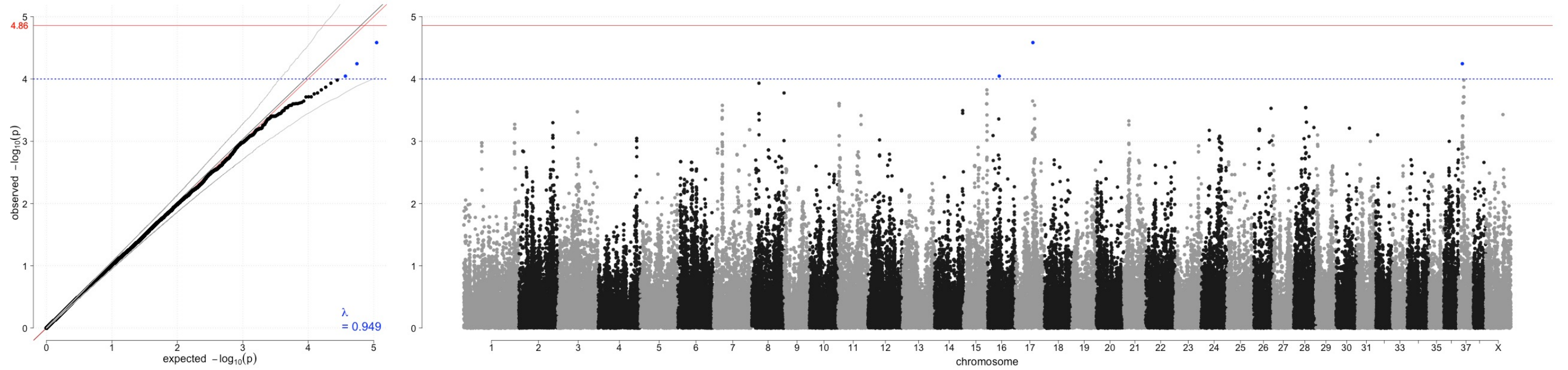

GWAS WORKING

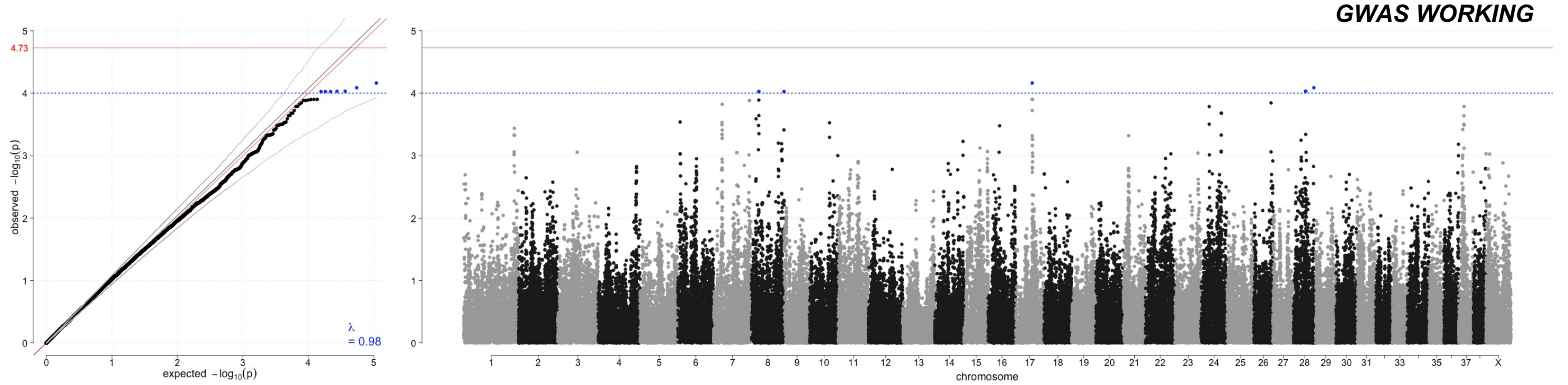

GWAS SHOW

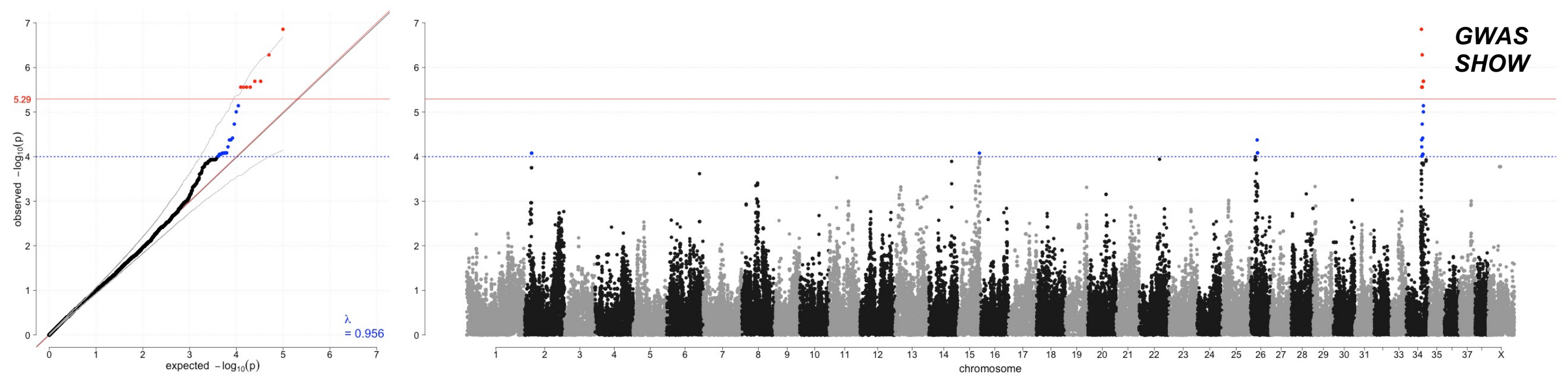

FOR GWAS RESULTS OBTAINED FOR THE WORKING AND SHOW SUB-POPULATIONS:

META-ANALYSIS FIXED EFFECTS

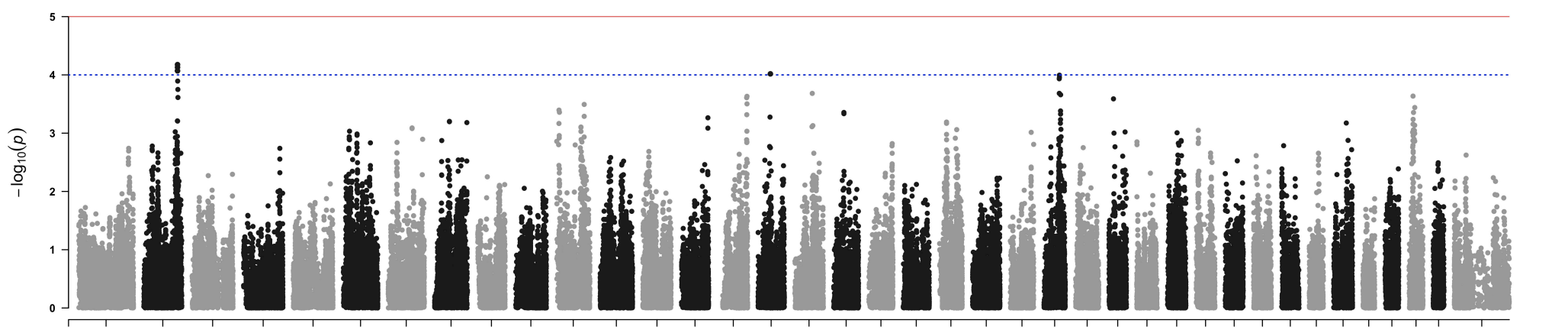

META-ANALYSIS RANDOM EFFECTS

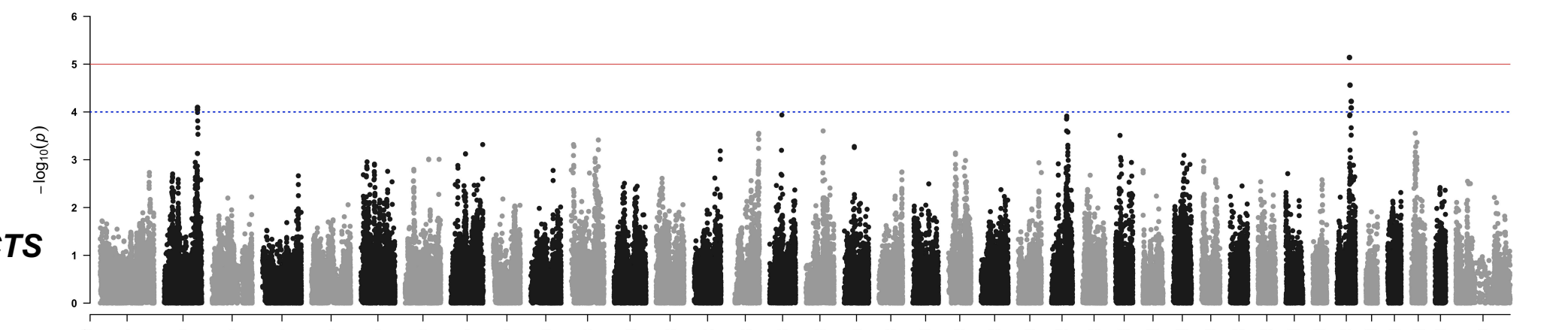

Trait: SOCIABILITY

SCORE DISTRIBUTIONS

ENTIRE COHORT (n=373) — WORKING (n=222) — SHOW (n=132)

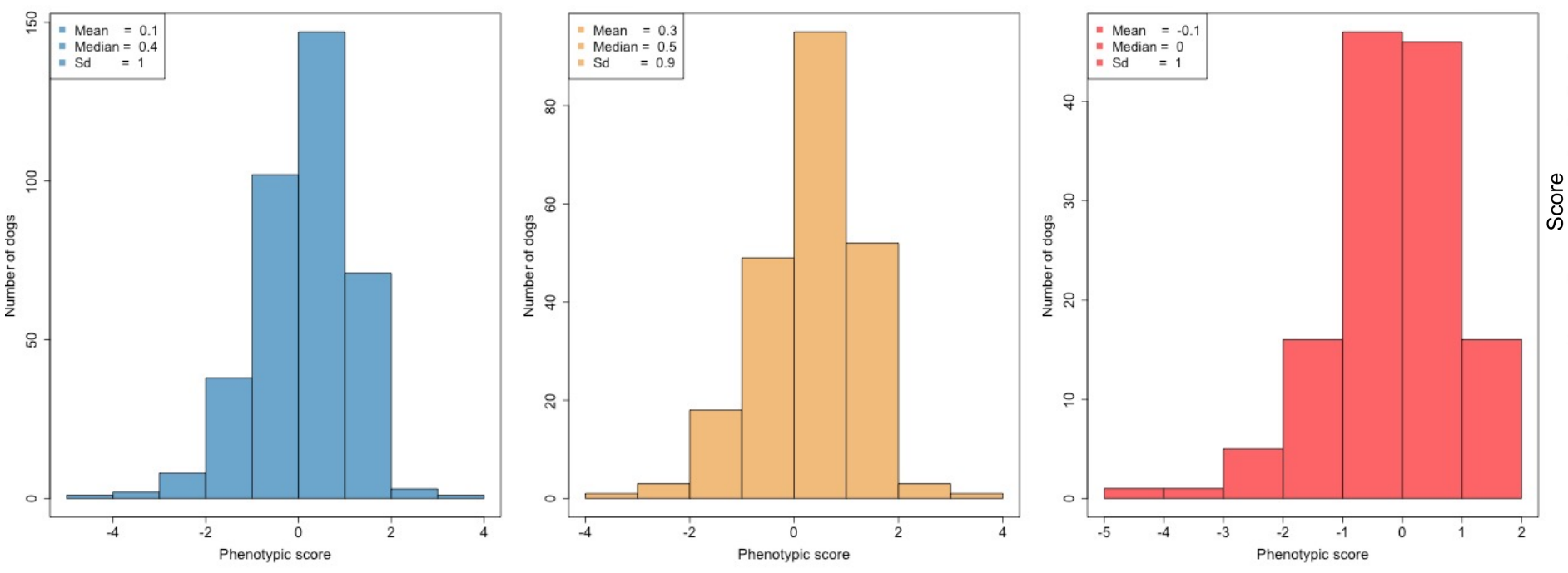

SCORE GRADIENT

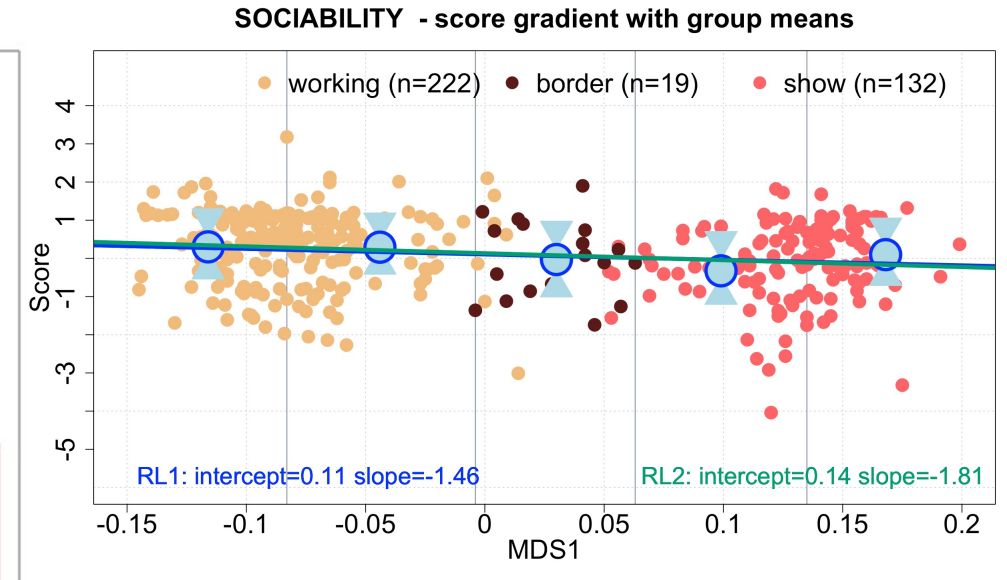

GWAS ENTIRE COHORT

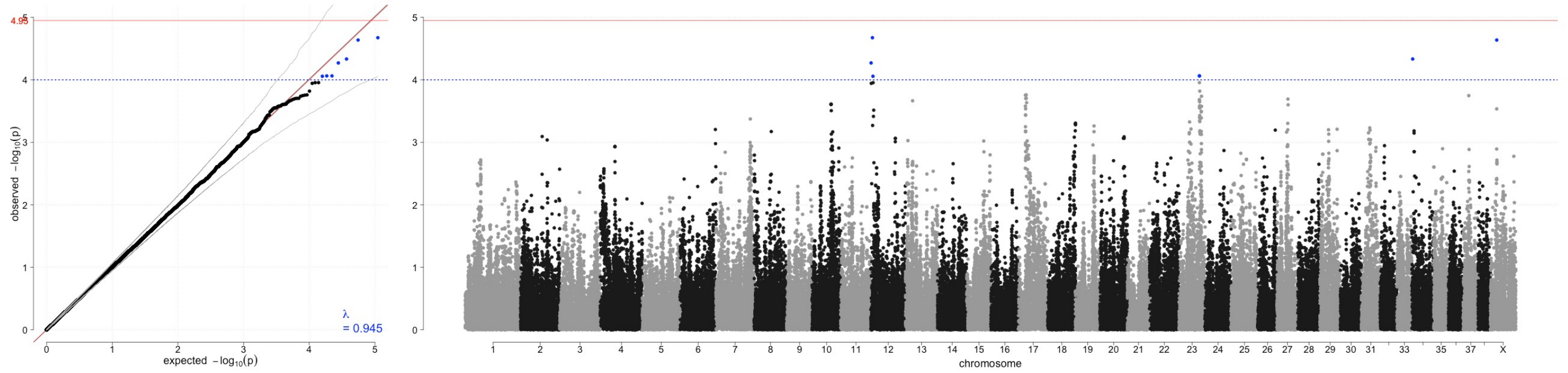

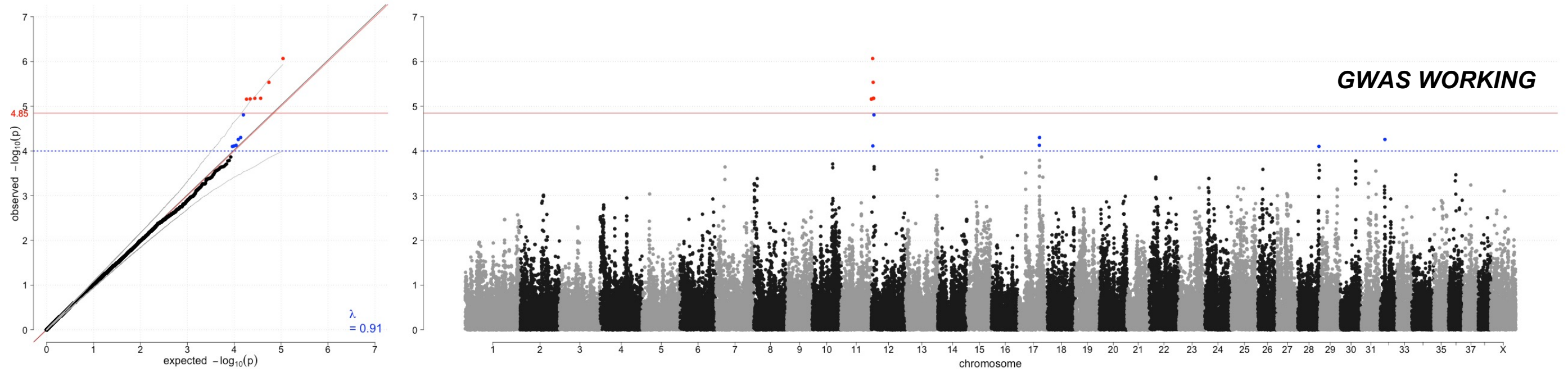

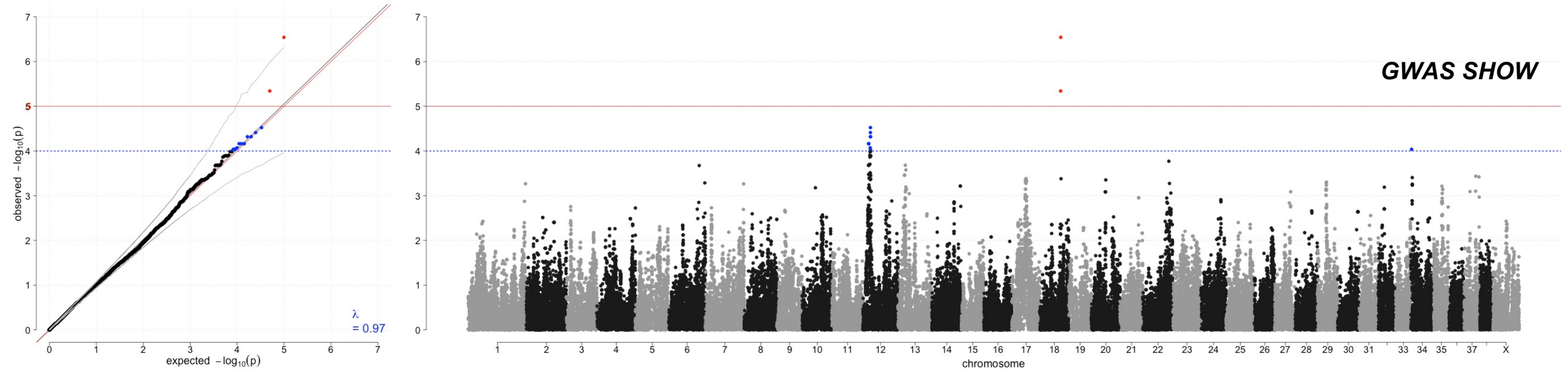

FOR GWAS RESULTS OBTAINED FOR THE WORKING AND SHOW SUB-POPULATIONS:

META-ANALYSIS FIXED EFFECTS

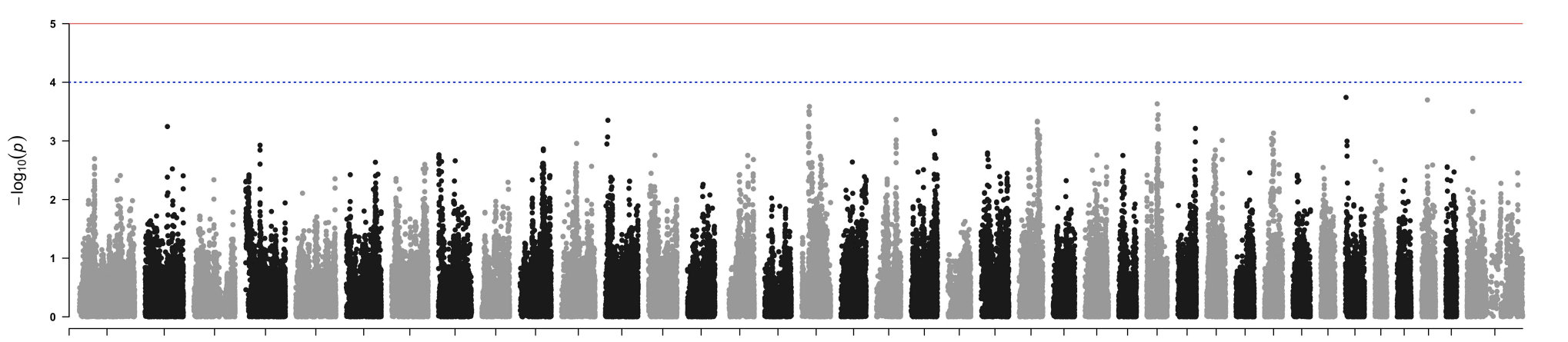

META-ANALYSIS RANDOM EFFECTS

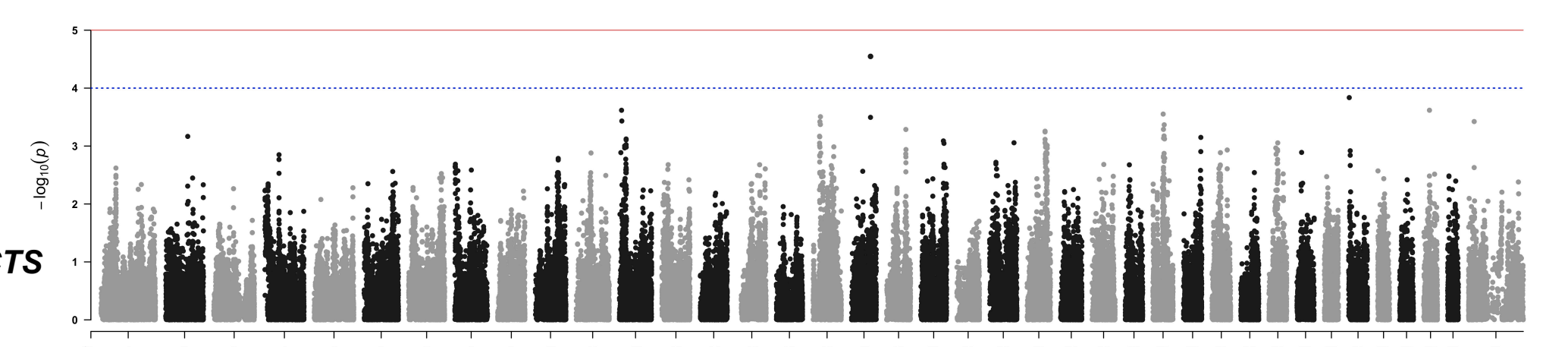

Behavioral variable: 1a. Greeting reaction

SCORE DISTRIBUTIONS

ENTIRE COHORT (n=373) — WORKING (n=222) — SHOW (n=132)

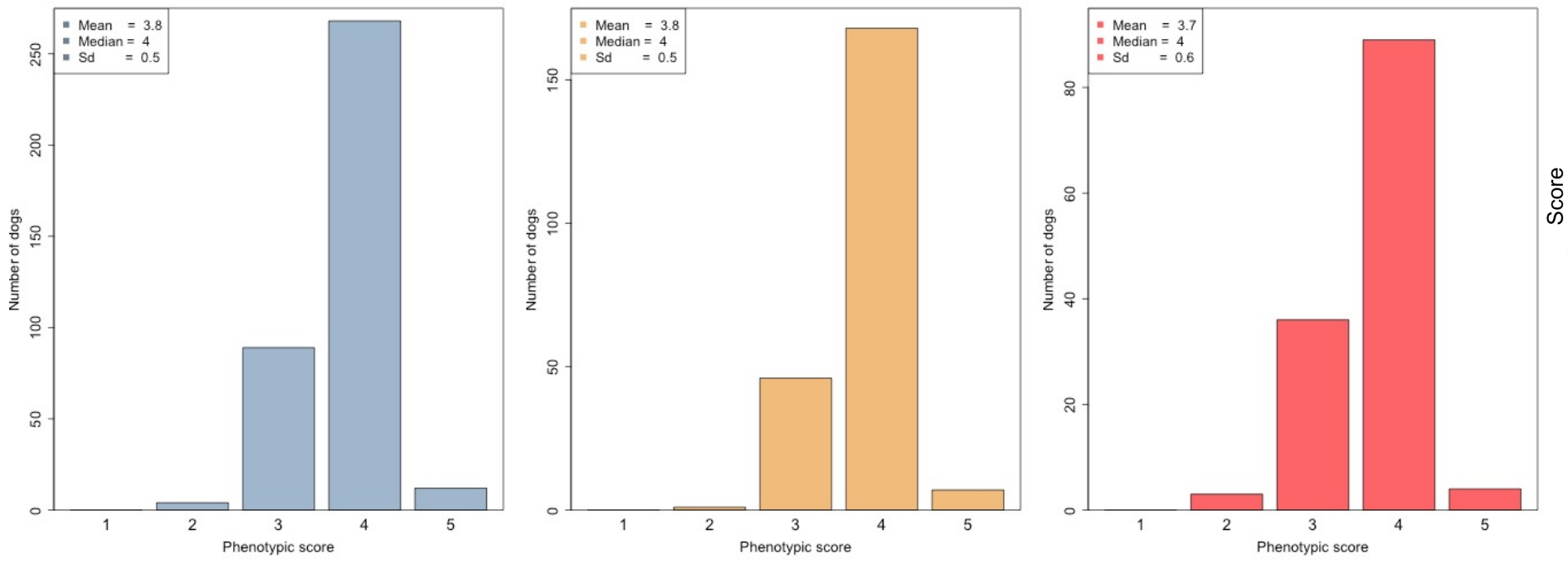

SCORE GRADIENT

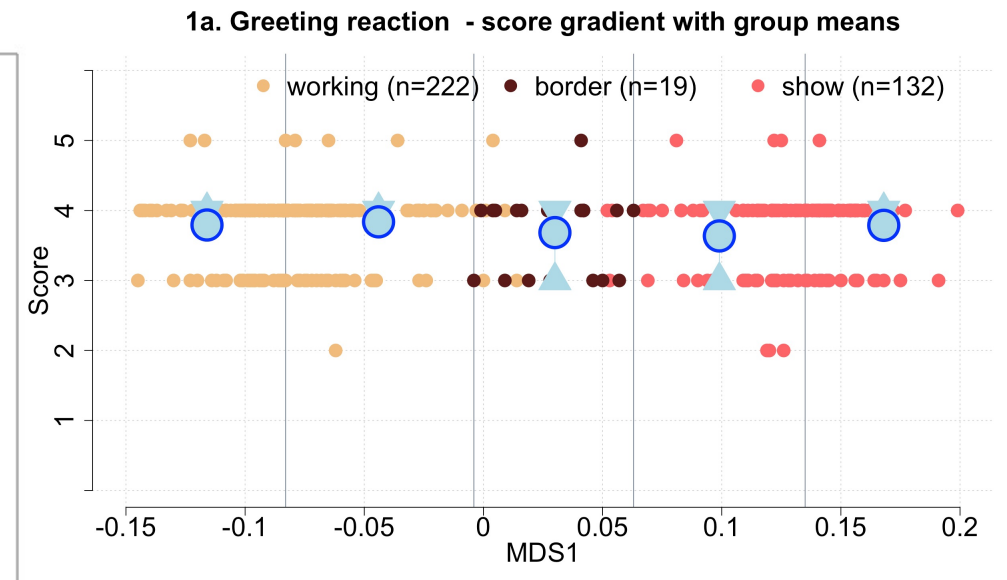

GWAS ENTIRE COHORT

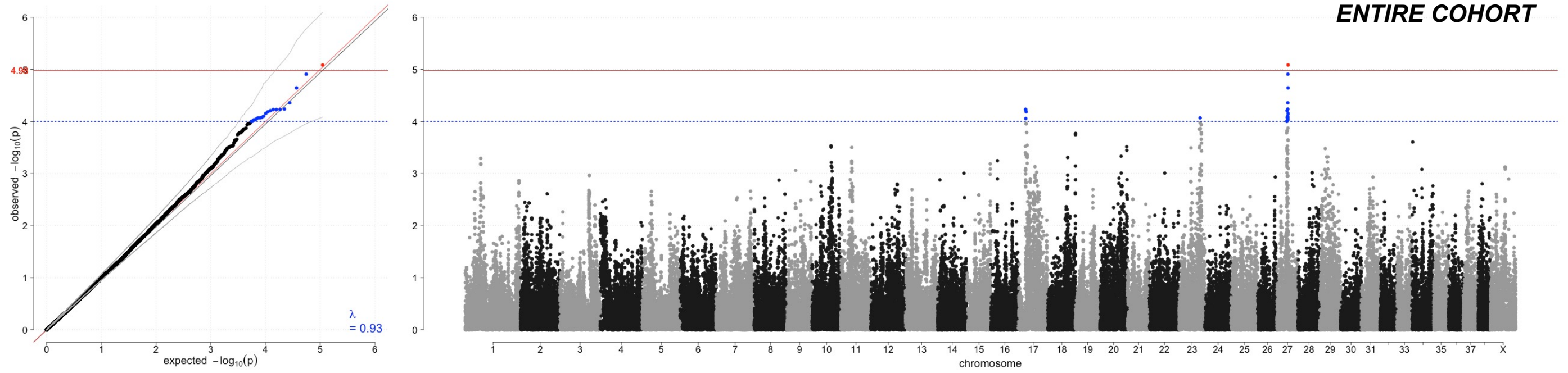

GWAS WORKING

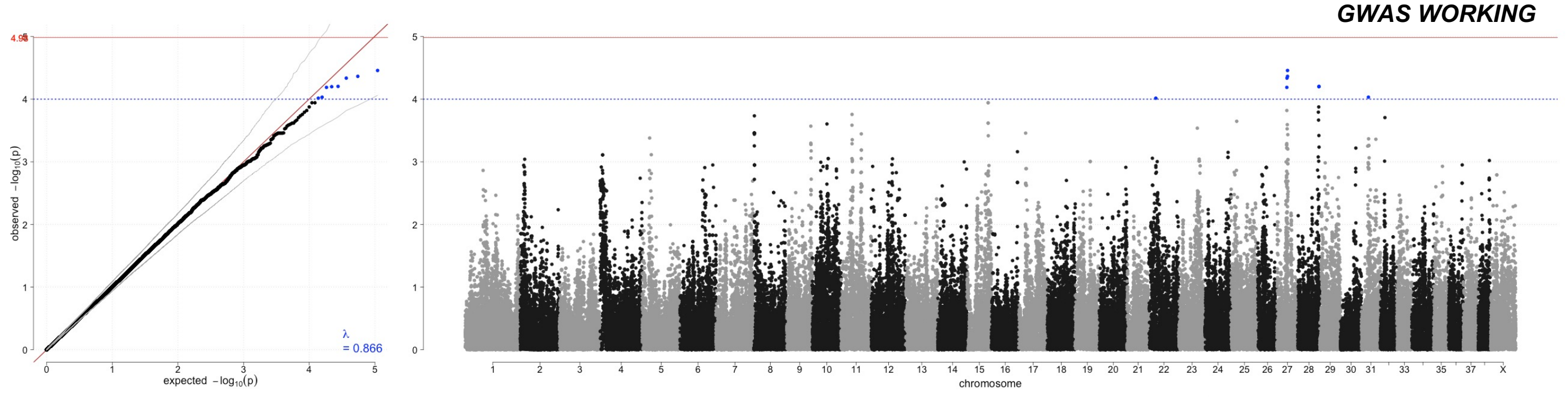

GWAS SHOW

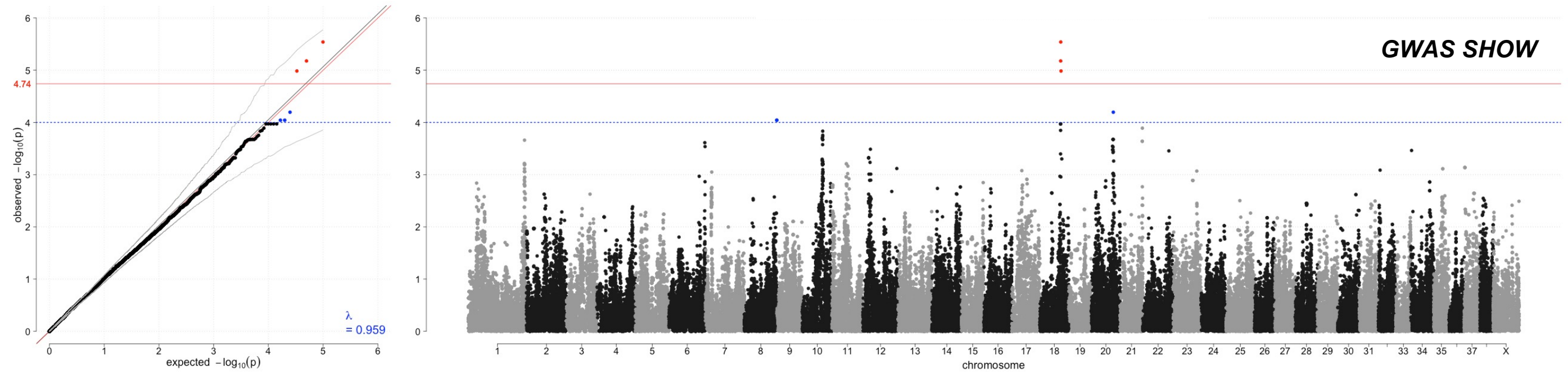

FOR GWAS RESULTS OBTAINED FOR THE WORKING AND SHOW SUB-POPULATIONS:

META-ANALYSIS FIXED EFFECTS

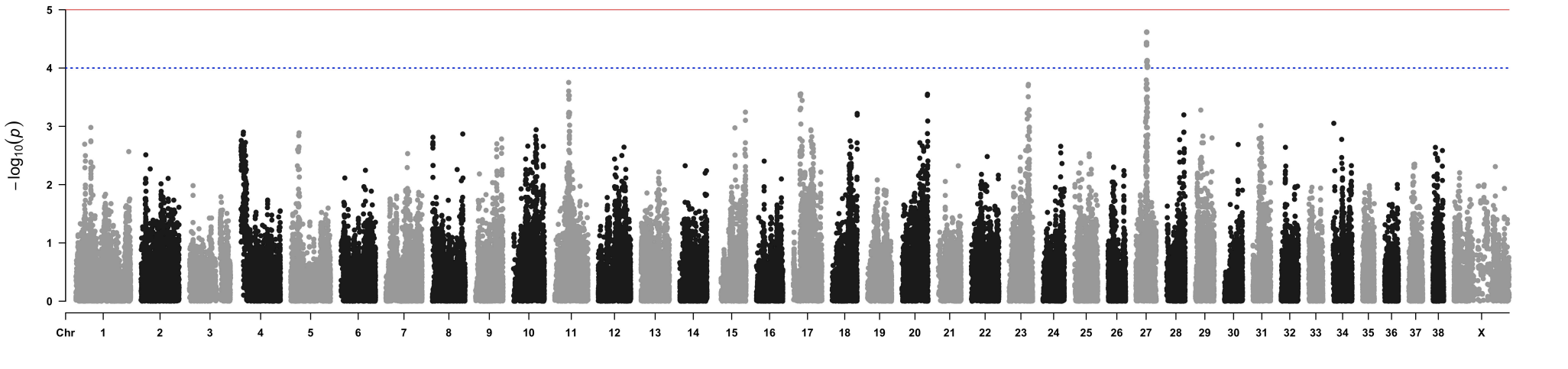

META-ANALYSIS RANDOM EFFECTS

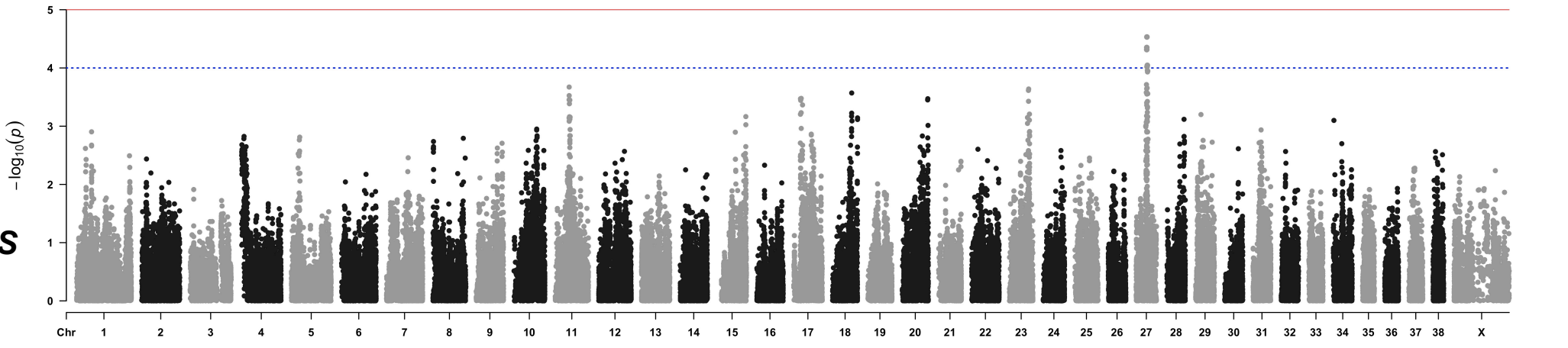

Behavioral variable: 1b. Cooperation

SCORE DISTRIBUTIONS

ENTIRE COHORT (n=373)

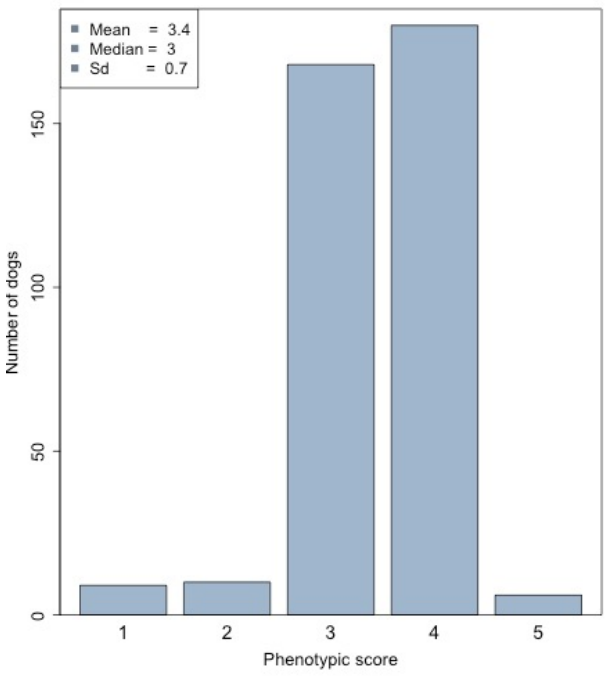

WORKING (n=222)

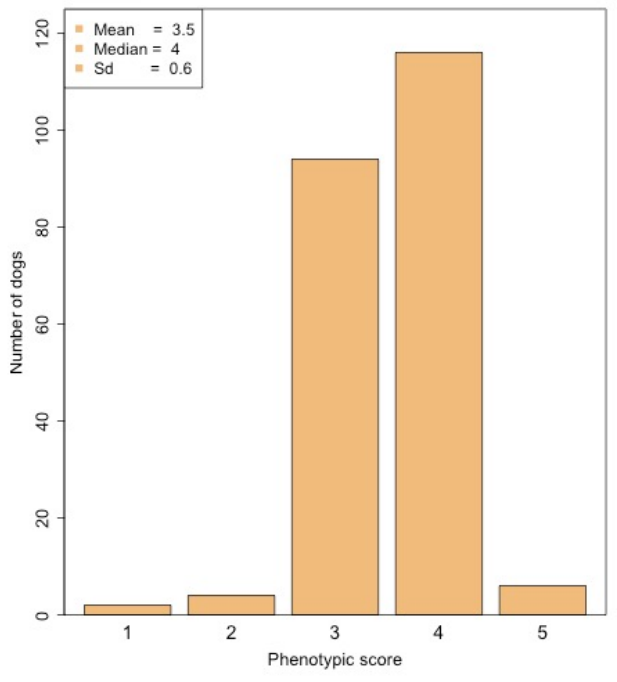

SHOW (n=132)

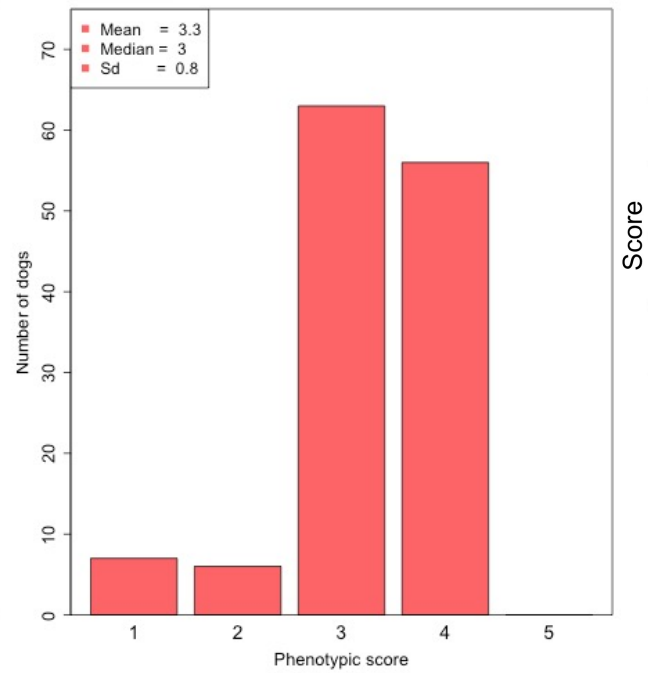

SCORE GRADIENT

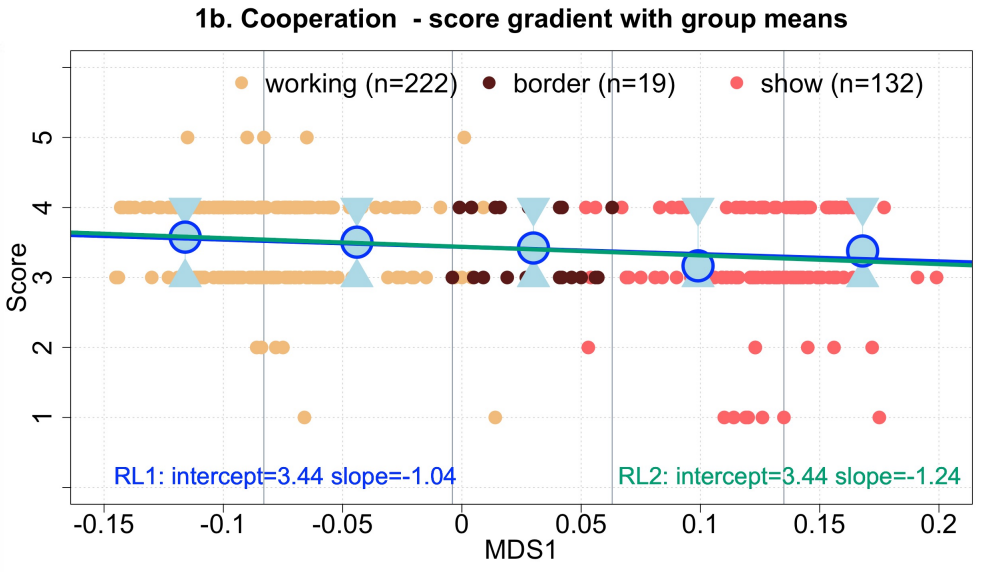

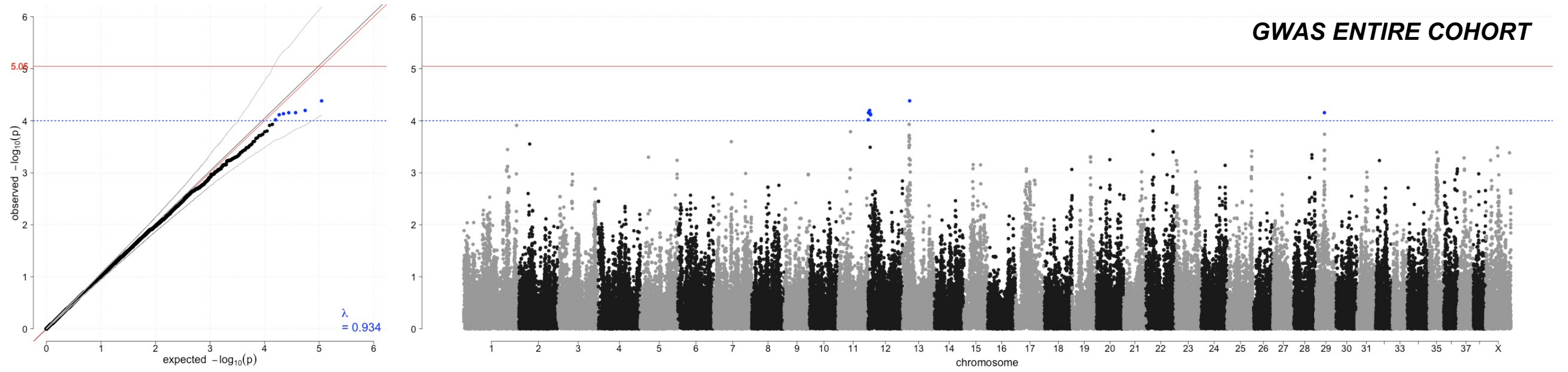

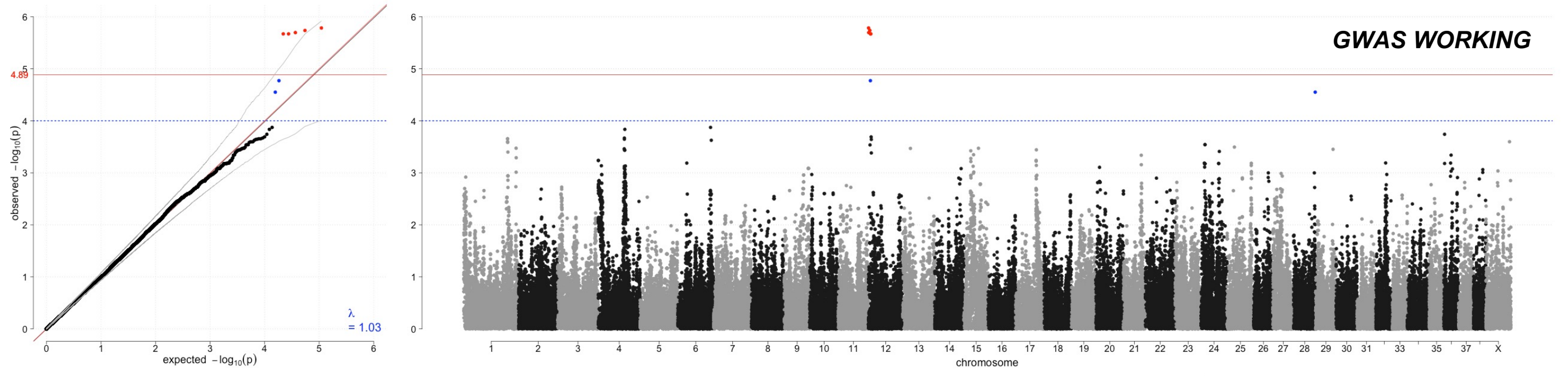

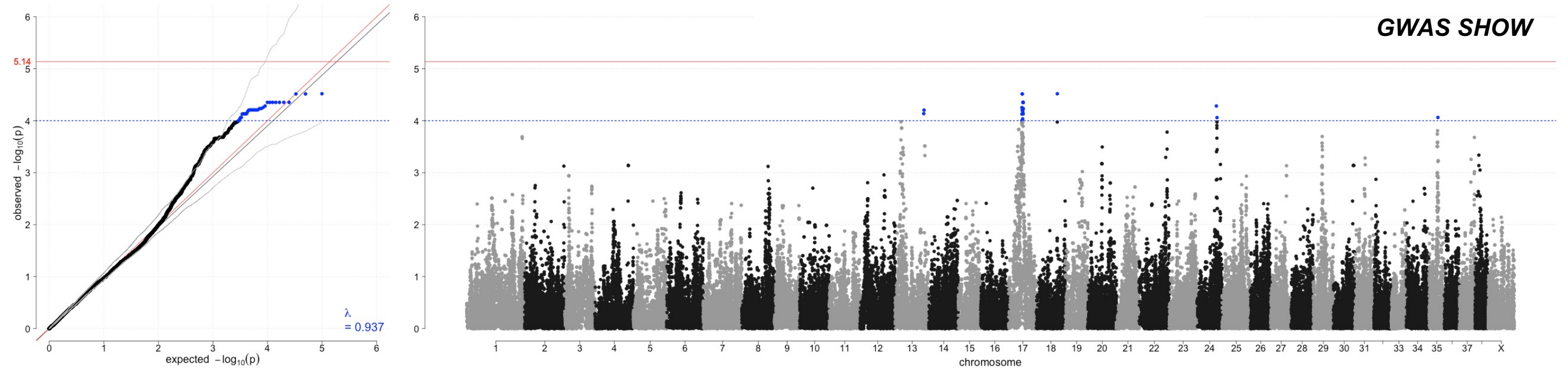

FOR GWAS RESULTS OBTAINED FOR THE WORKING AND SHOW SUB-POPULATIONS:

META-ANALYSIS FIXED EFFECTS

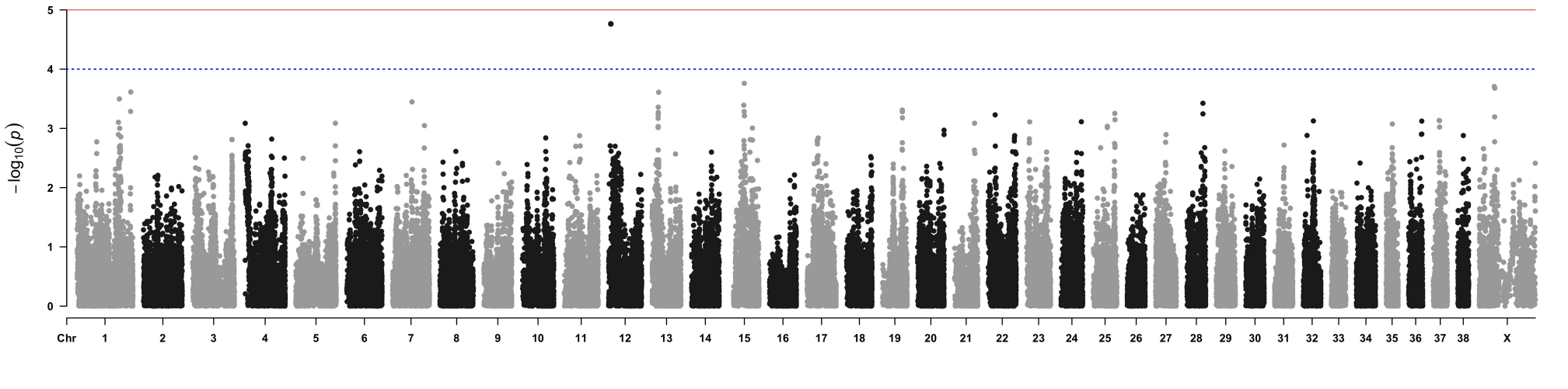

META-ANALYSIS RANDOM EFFECTS

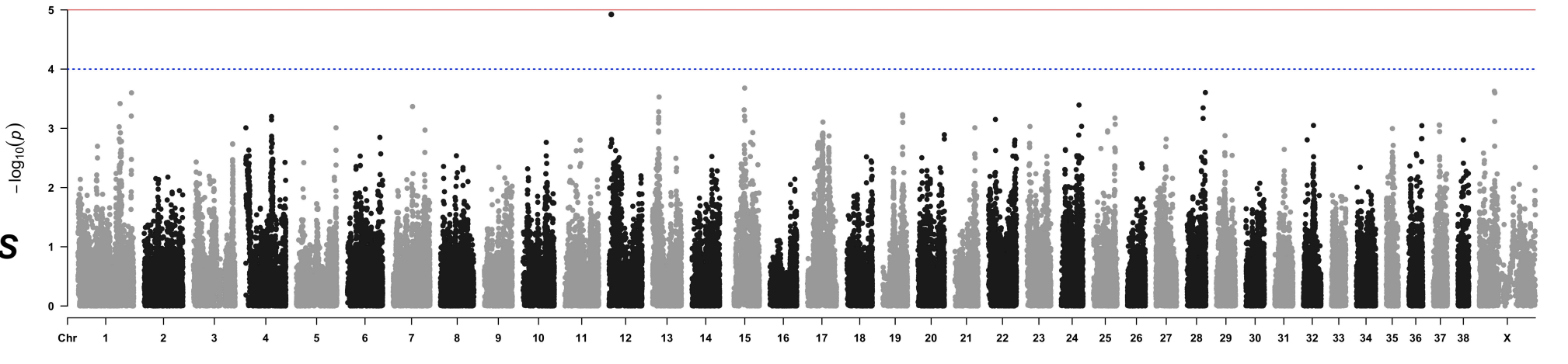

Behavioral variable: 1c. Handling

SCORE DISTRIBUTIONS

ENTIRE COHORT (n=373) | WORKING (n=222) | SHOW (n=132)

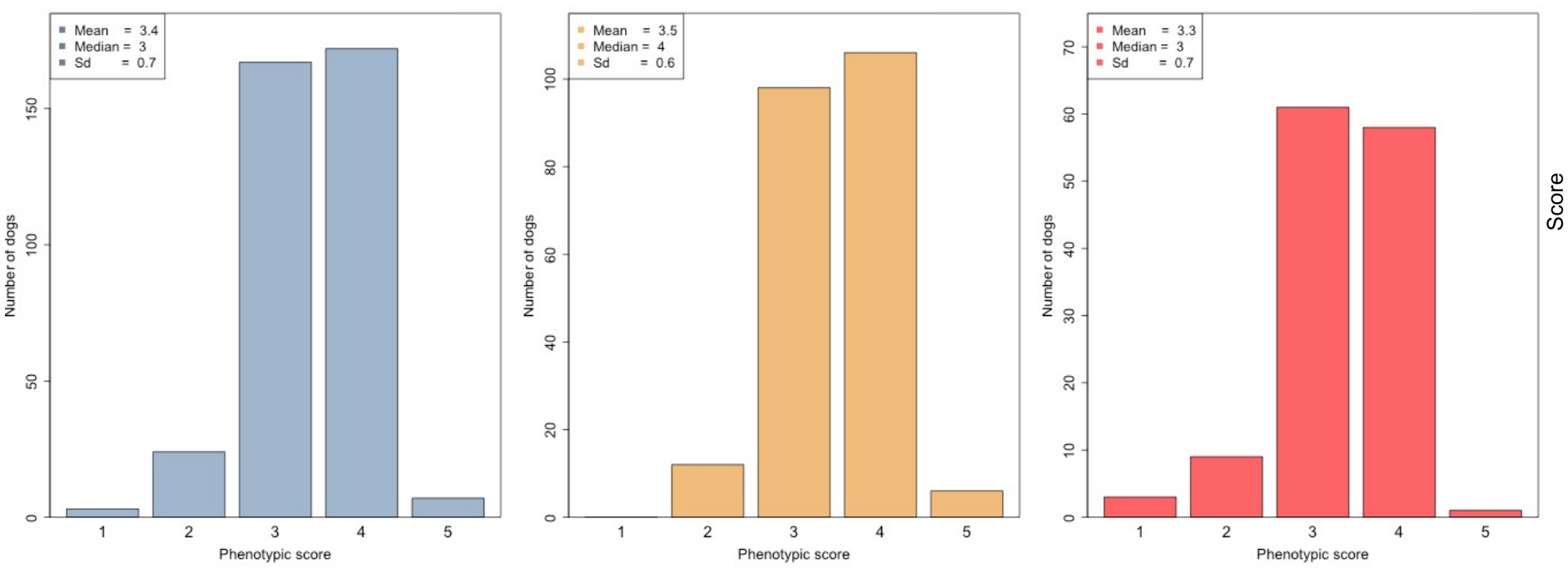

SCORE GRADIENT

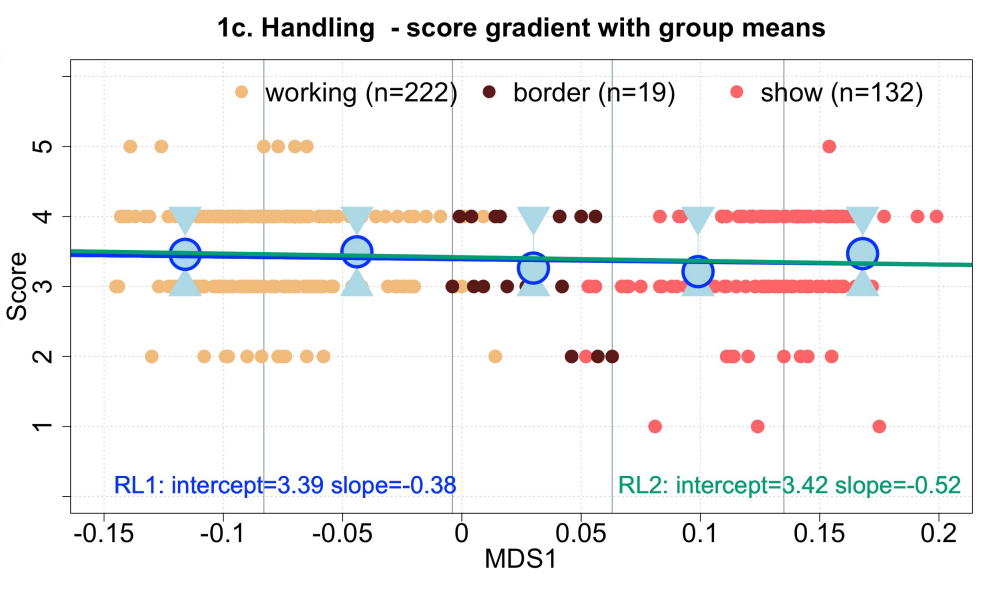

GWAS ENTIRE COHORT

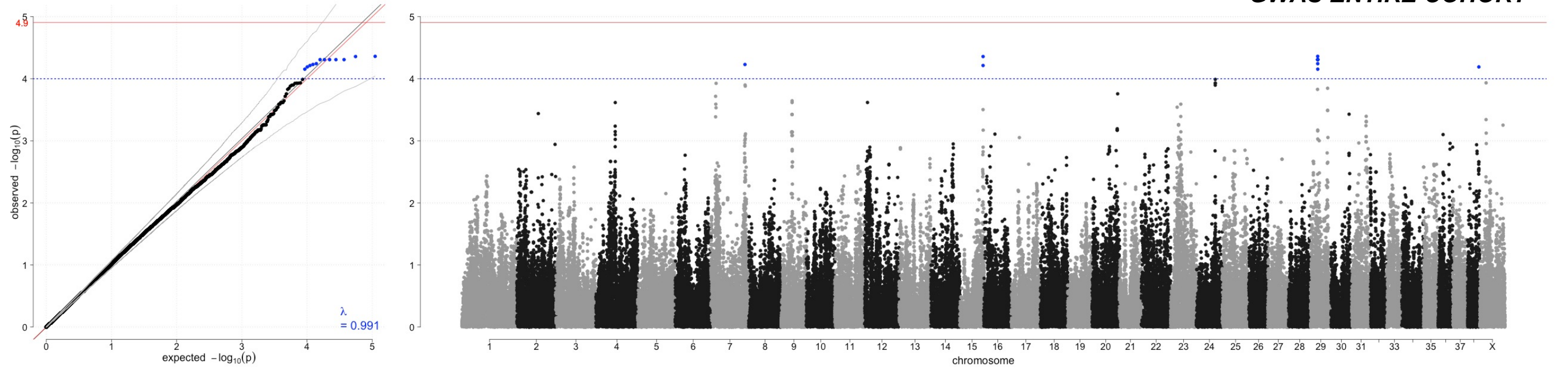

GWAS WORKING

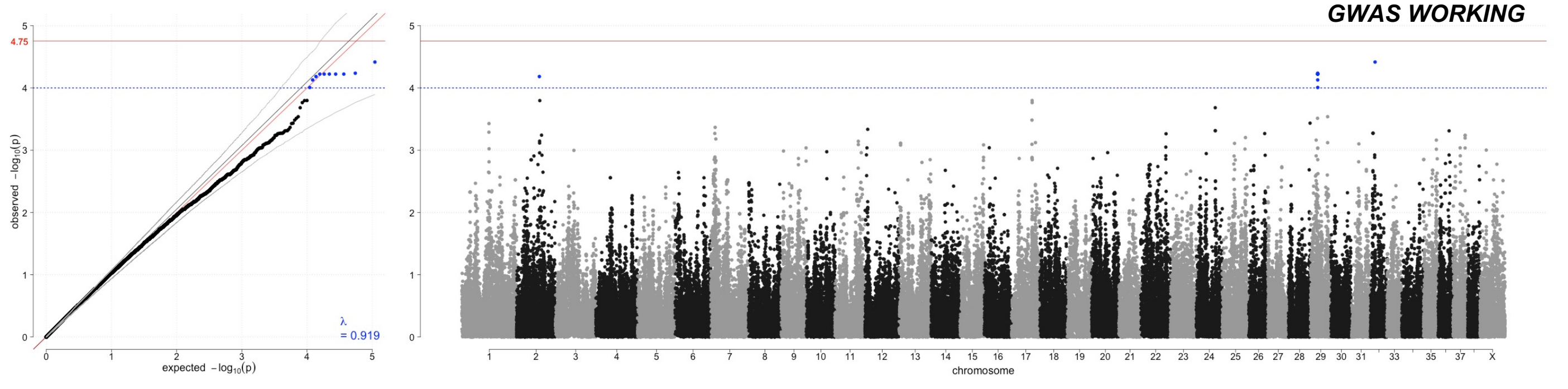

GWAS SHOW

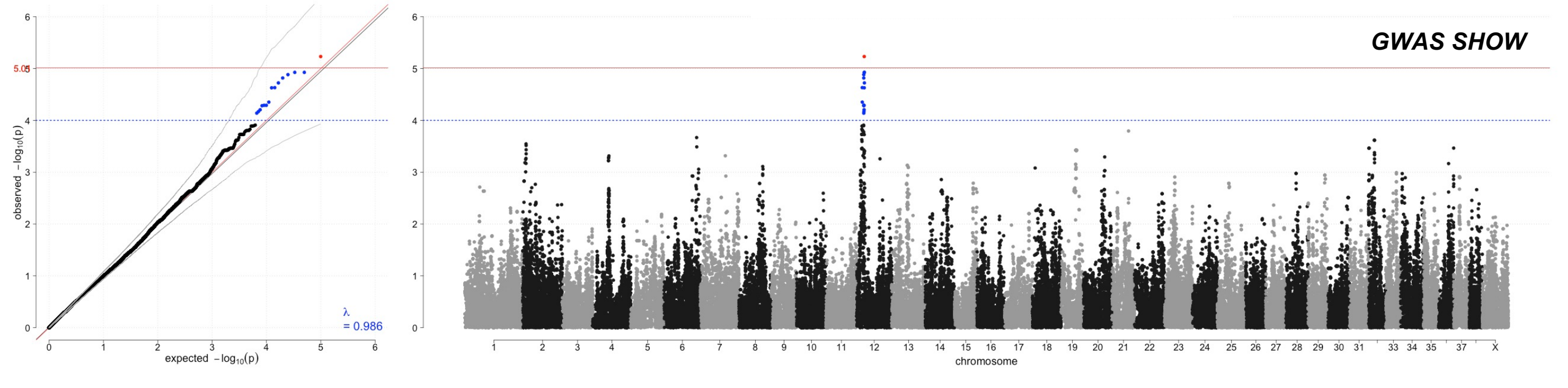

FOR GWAS RESULTS OBTAINED FOR THE WORKING AND SHOW SUB-POPULATIONS:

META-ANALYSIS FIXED EFFECTS

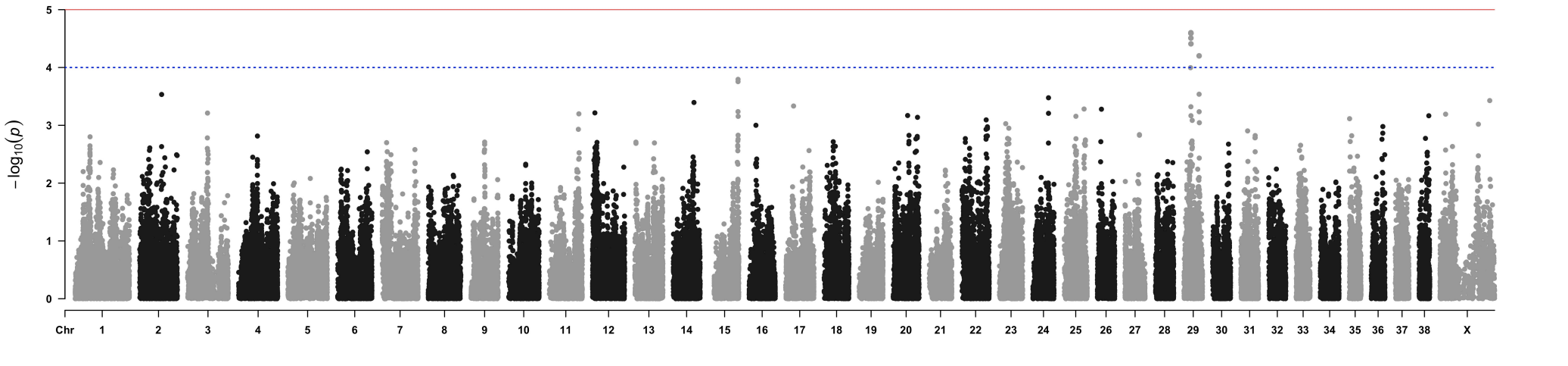

META-ANALYSIS RANDOM EFFECTS

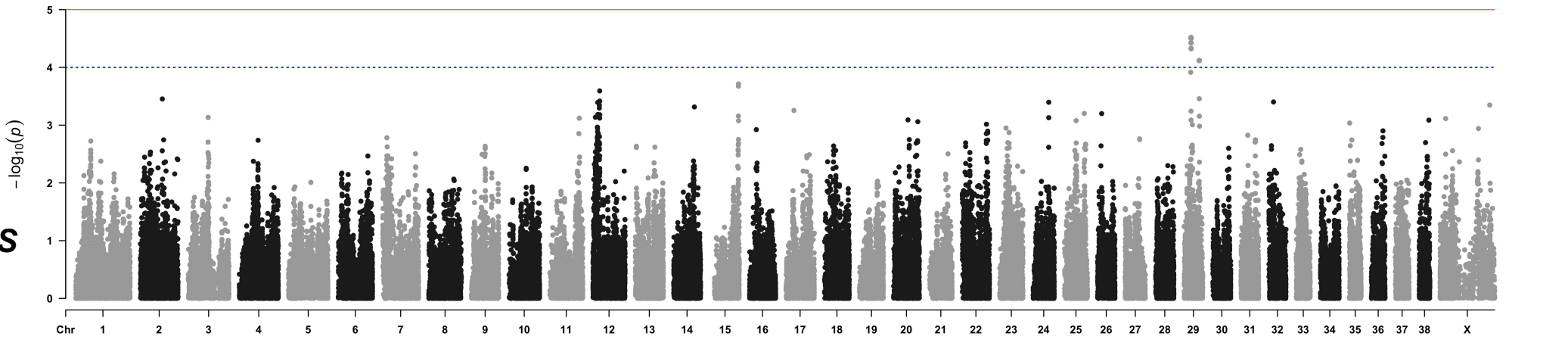

Behavioral variable: 6d. Remaining avoidance behavior

SCORE DISTRIBUTIONS

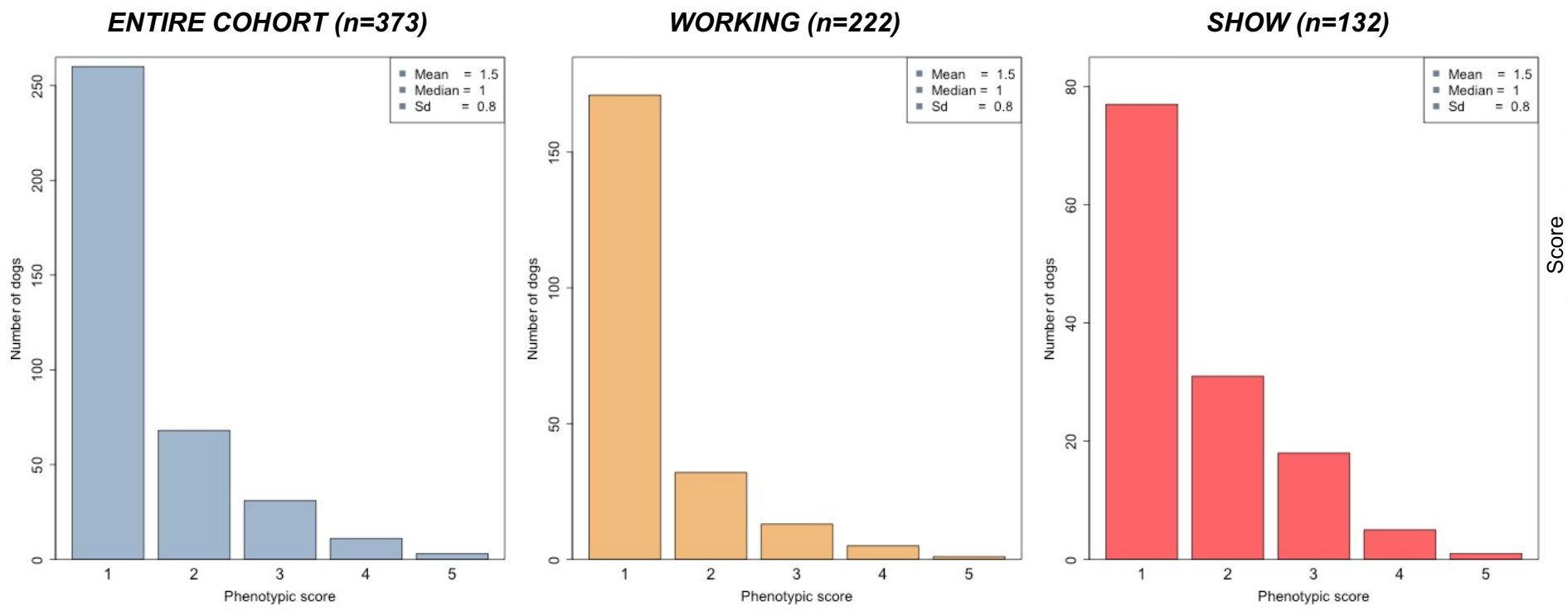

SCORE GRADIENT

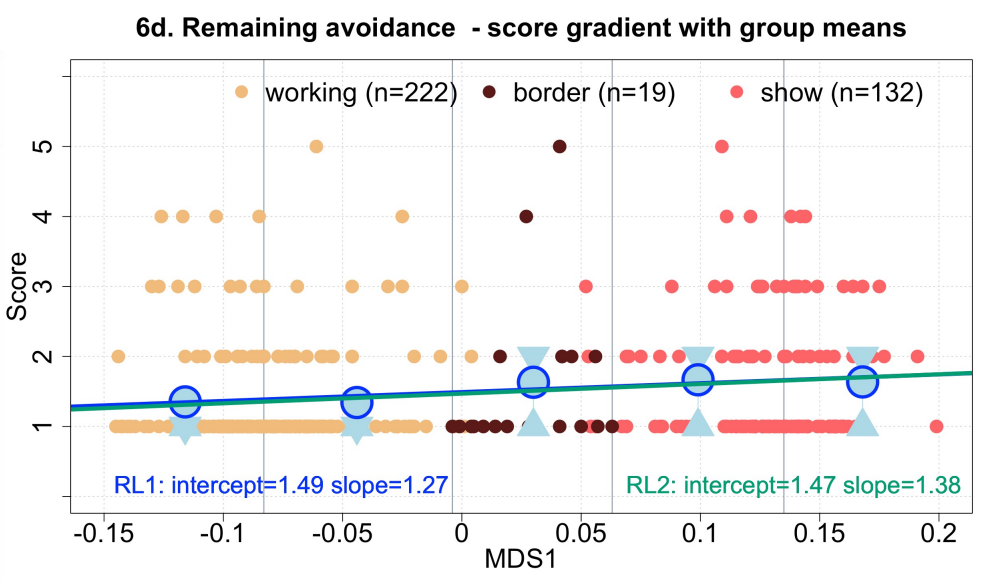

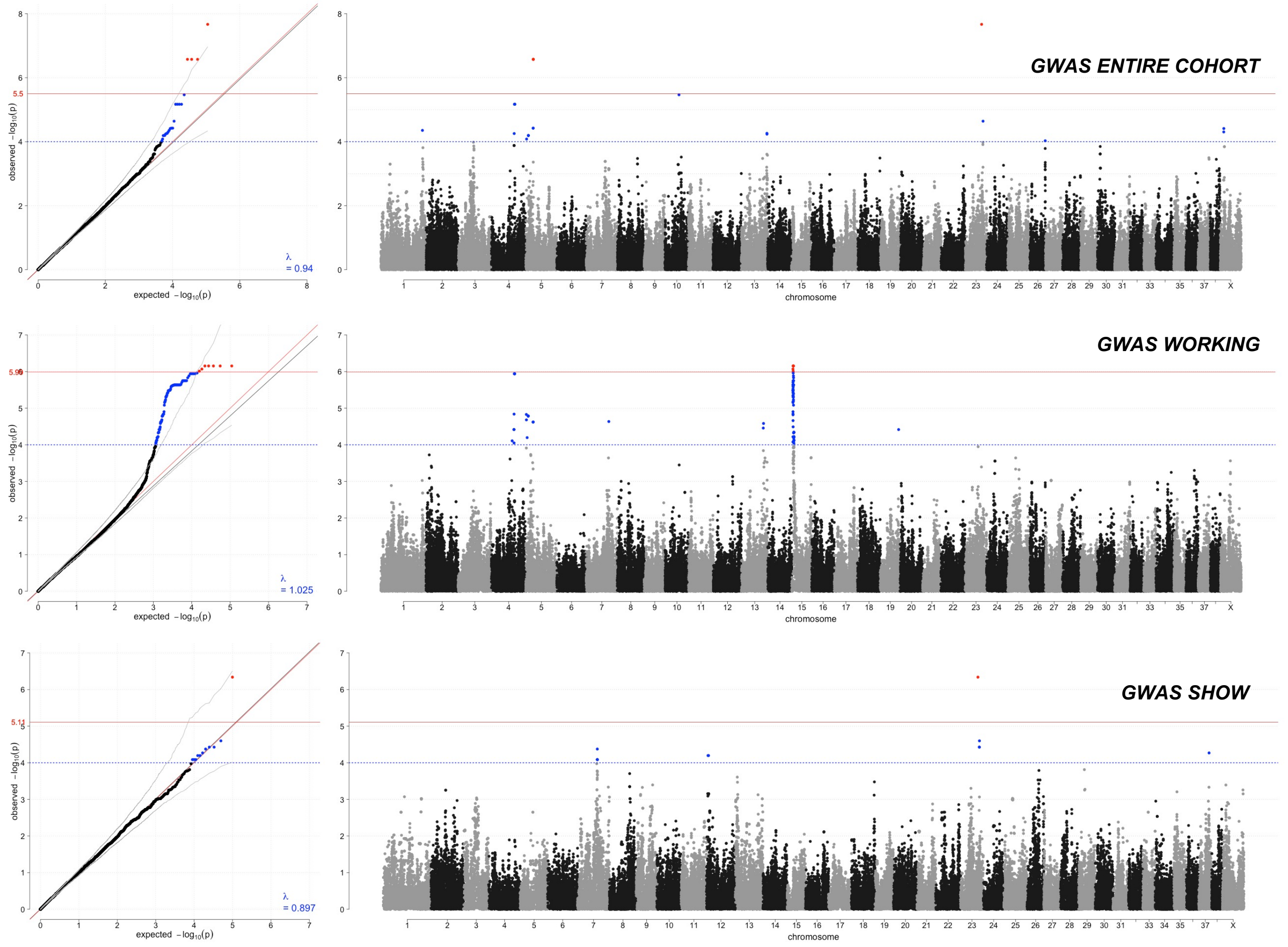

FOR GWAS RESULTS OBTAINED FOR THE WORKING AND SHOW SUB-POPULATIONS:

META-ANALYSIS FIXED EFFECTS

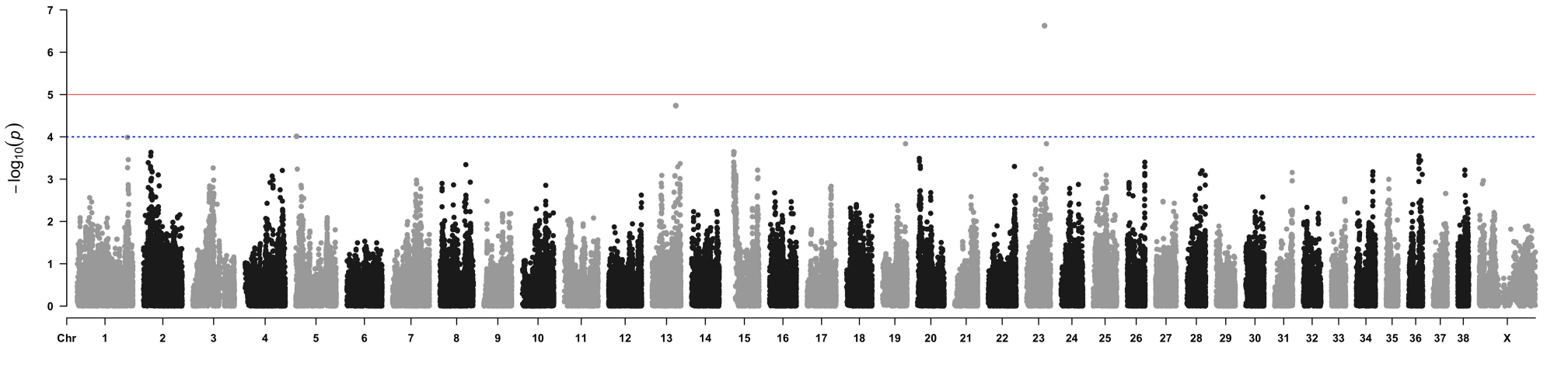

META-ANALYSIS RANDOM EFFECTS

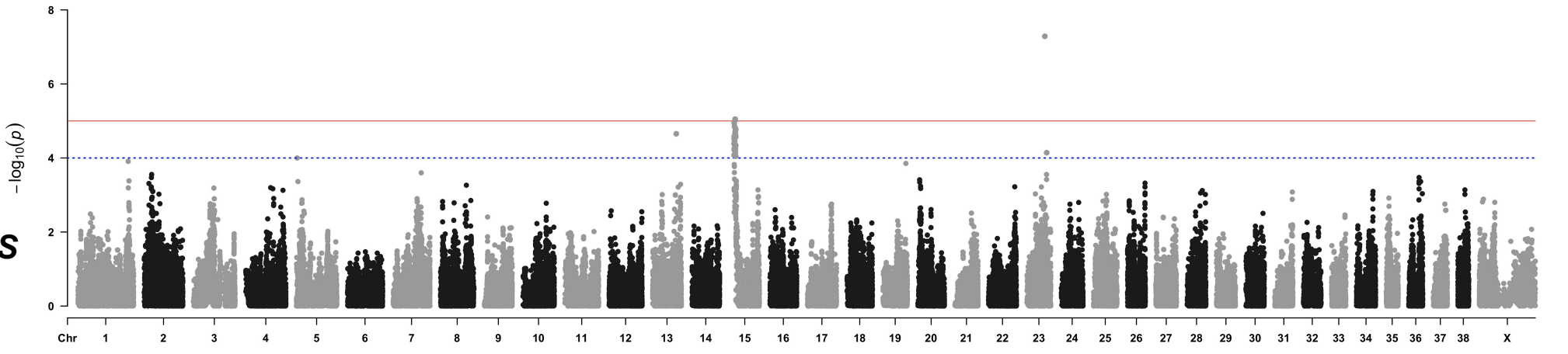

Supplement: Supplementary file 2 [file AGE-57-0-s002.pdf]
