# Supplementary material for: The Genetic Architecture of Behavior in the German Shepherd Dog
Source: Anim Genet. 2026 Nov 10;57(6):e70221. doi: 10.1002/age.70221 (PMC13752456; doi:10.1002/age.70221)

SUPPLEMENTARY FIGURES

Figure S1. Overview of the project. Boxed sections marked with letters correspond to the Results sections in parentheses. They describe the major aims and methodological steps within each section along with the corresponding figures and tables listed in the order presented in the text. In brief, DMA test scores for 23,109 SKK-registered GSD dogs were used to perform EFA identifying six first-order traits and one second-order trait, and the factor loadings were used to compute aggregated trait scores **(A).** From these, standardized trait/behavioral variable scores for a cohort of 373 genotyped GSDs were extracted. A population stratification analysis of this cohort indicated the presence of two main sub-populations, a *working*-type and a *show*-type. We analyzed the differences in allele frequencies across the genome and the differences in DMA trait/behavioral variable scores between the two sub-populations **(B)**. GWAS analyses **(C)** were carried out for each trait/behavioral variable in three separate runs: for the entire cohort (to identify associations that are common across all dogs in the cohort) and for the *working* and the *show* sub-populations separately (to identify associations that are specific to each sub-population). For 28 of the 40 traits/behavioral variables at least one genome-wide significant association was found in any of the three GWAS runs, amounting to a total of 737 unique significant (p lower than a threshold computed in each run based on 1000 permutations) and suggestive (p lower that 10^{-4}) SNPs. The strongest association signals were found for traits/behavioral variables that are most correlated (positively and negatively) with BOLDNESS **(D)**. These include, at the boldness end, *5d. Tug-of-war*, *DISTANCE PLAYFULNESS* and BOLDNESS, *PLAYFULNESS* and its components, *3b. Grabbing 1,* and *SOCIABILITY* and its components, while, at the shyness end, *6d. Remaining avoidance behavior*. The association signals were analyzed for their genomic location and genotype-phenotype relationships. Genomic regions of interest in our study were also cross-referenced with previously reported regions of selection and domestication. Genes contained within the regions of association were annotated based on phenotypic information available in mice and humans, and positions of SNPs within selected signals were cross-referenced with variation data reported for comparable human personality traits.

Figure S2. Factor diagram and correlation coefficients between traits. (a) Factor diagram showing only behavioral variables with loadings greater than 0.35 or smaller than -0.35. In agreement with previous reports, the first-order factors correspond to *SOCIABILITY, PLAYFULNESS, DISTANCE PLAYFULNESS, CHASE-PRONENESS, CURIOSITY/FEARLESSNESS* and *AGGRESSIVENESS*, and the second-order factor to BOLDNESS. **(b-c)** Correlation coefficients between the computed scores for the first and second order factors and radar plots of mean score

values for the behavioral variables presented in Panel A across the SKK-registered GSDs analyzed (*n=23,109*) and the genotyped cohort used for GWAS (*n=373*), respectively.

Figure S3. GWAS results. **(a)** The twenty-eight traits/behavioral variables showing at least one genome-wide significant association in any of the three runs (for the entire cohort and the *working* and *show* sub-populations, respectively, Table S6). **(b-c)** Chromosomal distribution as density within a 1Mb window of the significant SNPs **(b)** and of the significant plus the suggestive SNPs **(c)** identified.

Figure S4. Genotype-phenotype relationships for SNPs associated with *5d. Tug-of-war*, *DISTANCE PLAYFULNESS* and BOLDNESS (pertaining to Supporting Information Results SR3). **(A-E)** Genotype-phenotype relationships between the *5d. Tug-of-war* phenotype (as the DMA score on a 1-5 scale) and the genotype of selected SNPs in the *show* (red) and *working* (beige) sub-populations as follows: **(A)** top SNP on CFA1 (BICF2S23248133), **(B)** the significant SNP on CFA2 (BICF2S23535265), **(C)** by-way relationships between the BICF2S23248133 and BICF2S23535265 SNPs, **(D)** the suggestive SNP in the *MC2R* promoter (BICF2P491494), **(E)** by-way relationships between the top SNP on CFA1 (BICF2S23248133) and the suggestive SNP in the *MC2R* promoter (BICF2P491494). The major (M) and minor (m) alleles are indicated within square brackets followed by the resulting genotypes. In all boxplot distributions, mean and median values are indicated by square black dots and black lines, respectively. **(F-G)** Genotype-phenotype relationships between the *DISTANCE PLAYFULNESS* and BOLDNESS phenotypes (as standardized scores) and the genotype of the top SNP for these traits on CFA1 (BICF2P1398938).

Figure S5. Significant and suggestive association signals for *PLAYFULNESS* and its contributing behavioral variables. **(a)** LD Manhattan plots for *PLAYFULNESS* and its contributing behavioral variables *2a. Interest in play*, *2b. Grabbing*, *2c. Tug-of-war*, *9a. Interest in play* and *9b. Grabbing*. Each plot refers to the GWAS performed in the dataset in which a significant or suggestive signal was found (*i.e.* entire cohort, *show* and *working* sub-populations, respectively). Only the regions surrounding the significant/suggestive SNPs are shown. The top SNP is represented in magenta and with a cross; the remaining SNPs are color coded based on their degree of LD (r^2) with the top SNP. Dotted bars represent the thresholds for classifying a SNP as significant (red, based on 1000 permutations) or suggestive (blue, $-\log_{10}p >= 4$). Gray boxes highlight the genes in which the significant/suggestive SNPs are located (bold italics font) or the genes immediately upstream or downstream of the SNPs (regular italics font). Some association signals containing only

one suggestive/significant SNP for which no other SNPs in LD were found were omitted from this representation. For complete results, see Table S6 and Table S8. **(b)** Genomic region for the *2b. Grabbing* signal on CFA16 around the *UNC5D* locus showing, as custom UCSC tracks, the -log10*p* values and the suggestive/significant SNPs in LD with the top SNP obtained in the GWAS performed in the entire cohort (EC, light blue) and the *show* sub-population (red). **(c)** Genotype-phenotype plots for the *2b. Grabbing* top SNP BICF2P811419.

Figure S6. Significant GWAS results in the *show* sub-population for the *3b. Grabbing 1* behavioral variable contributing to *CHASE-PRONENESS*. **(a)** Q-Q and Manhattan plots for *3b. Grabbing1* in the *show* sub-population showing a genome-wide significant association on CFA34. **(b)** LD-Manhattan plot of the CFA34:27-33Mb region showing the SNPs in LD with the top SNP BICF2S23516853 (represented in magenta and marked with a cross). **(c)** Genotype-phenotype plots for the top SNP BICF2S23516853 in the entire cohort and the *show* and *working* sub-populations, respectively. Phenotypic values are original DMA ratings on a 1 to 5 scale. The major allele (M) and the minor allele (m) are presented in square brackets. In the boxplot distributions, mean and median values are indicated by square black dots and black lines, respectively. **(d)** The region of association presented alongside two UCSC custom tracks displaying the -$\log_{10}p$ values (first track, green) for the identified significant and suggestive SNPs (second track, red). SNP names are annotated to reflect the significance and the rank in the GWAS (by *p*) of the significant or suggestive SNPs (*TS* stands for top SNP, *S* for significant and *s* for suggestive).

Figure S1. Overview of the project.

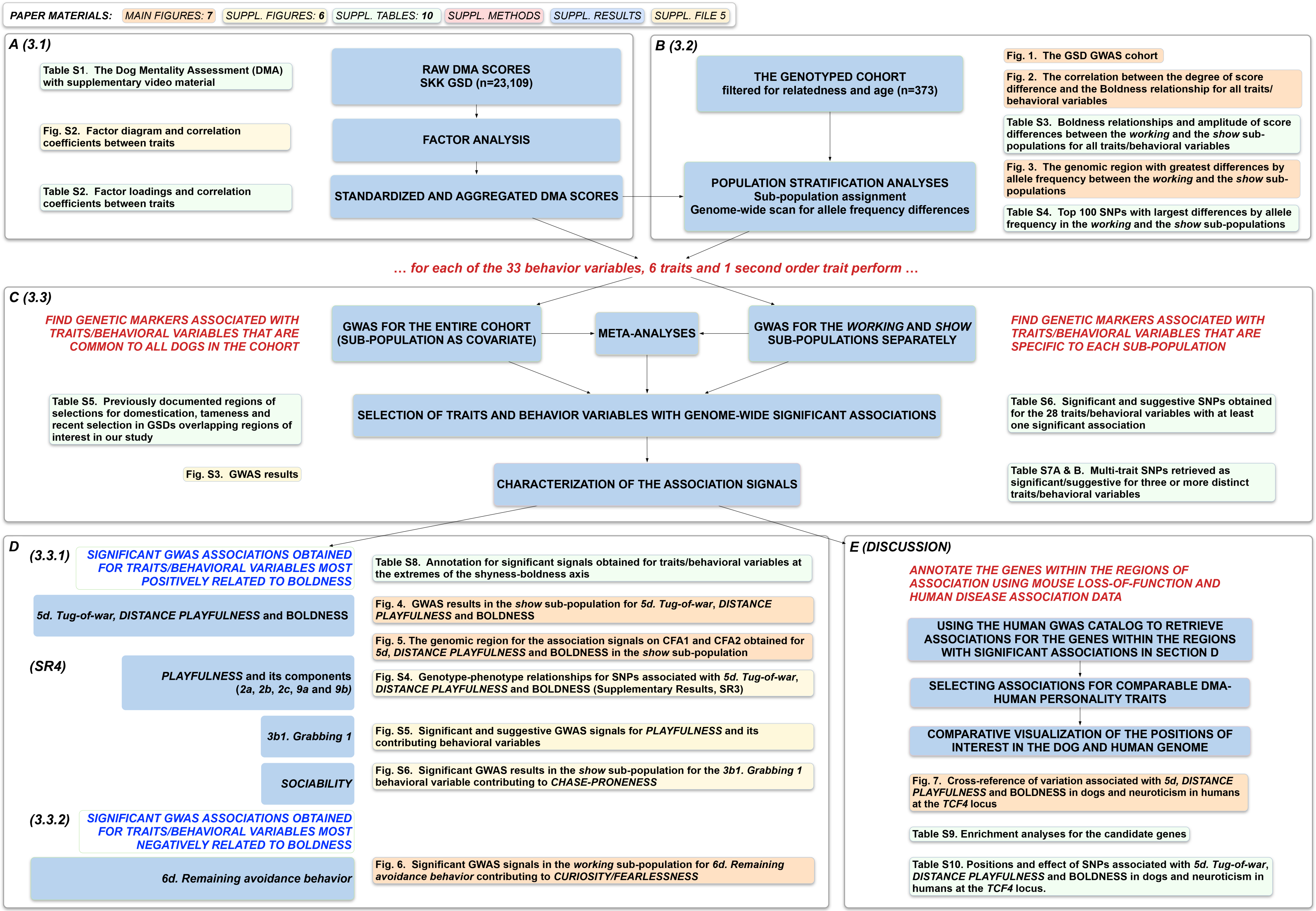

Figure S2. Factor diagram and correlation coefficients between traits.

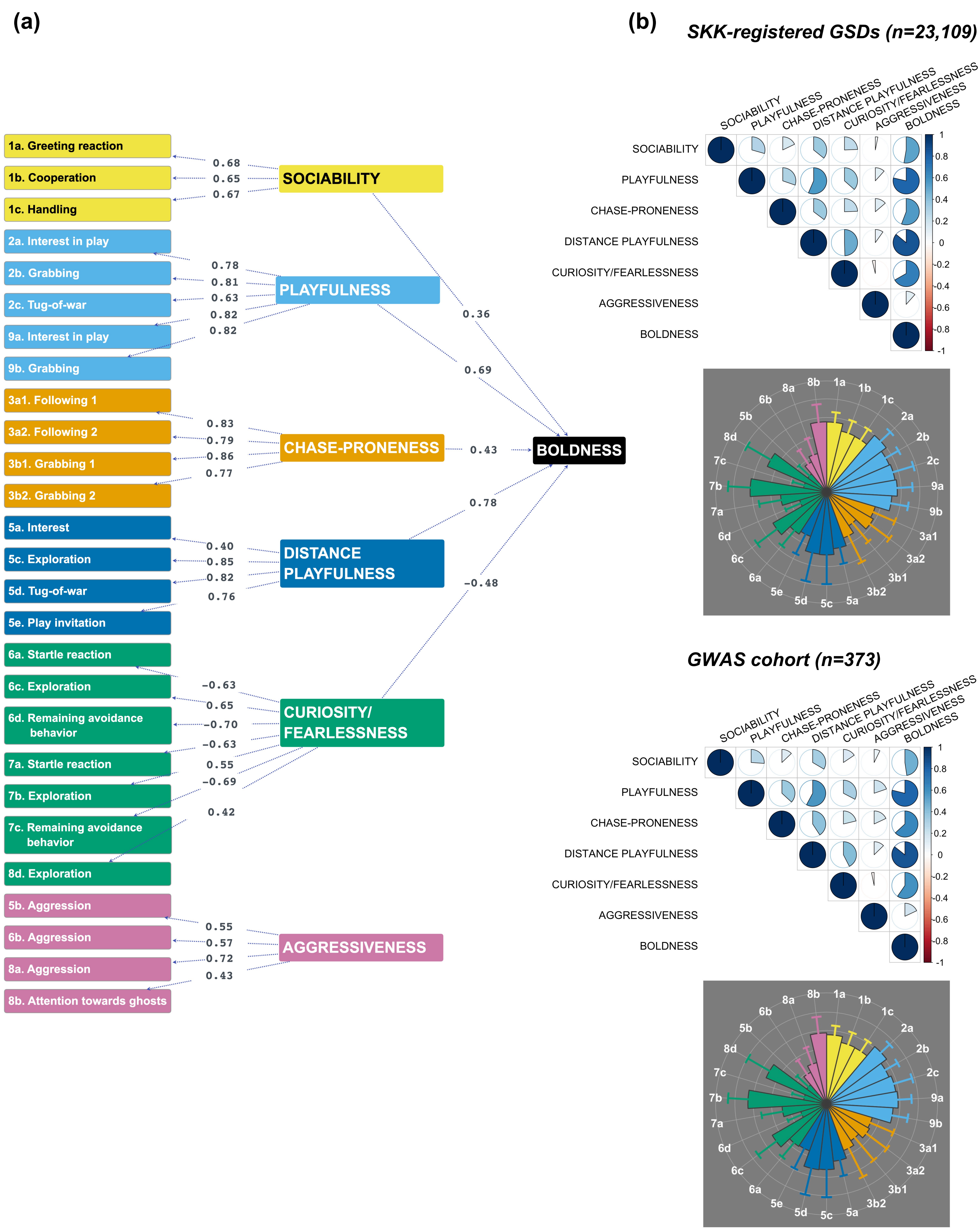

Figure S3. GWAS results.

(a)

TRAIT/BEHAVIORAL VARIABLE	GENOME-WIDE SIGNIFICANT RESULTS OBTAINED FOR THE ENTIRE COHORT	GENOME-WIDE SIGNIFICANT RESULTS OBTAINED FOR THE *WORKING* SUB-POPULATION	GENOME-WIDE SIGNIFICANT RESULTS OBTAINED FOR THE *SHOW* SUB-POPULATION
2c. Tug-of-war	Yes	Yes	Yes
6d. Remaining avoidance behavior			
5b. Agression			
8b. Attention towards ghosts			
2b. Grabbing	Yes	Yes	
3a2. Following 2			
6b. Aggression			
CHASE-PRONENESS	Yes		Yes
1a. Greeting reaction			
2a. Interest in play			
3b2. Grabbing 2	Yes		
5c. Exploration			
6c. Exploration			
8c. Avoidance behavior			
9b. Grabbing			
SOCIABILITY		Yes	Yes
5a. Interest			
7d. Remaining approach behavior			
8d. Exploration			
**BOLDNESS**			Yes
DISTANCE PLAYFULNESS			
5d. Tug-of-war			
5e. Play invitation			
9a. Interest in play			
1c. Handling			
3b1. Grabbing 1			
6e. Remaining approach behavior			
8a. Aggression			

(b)

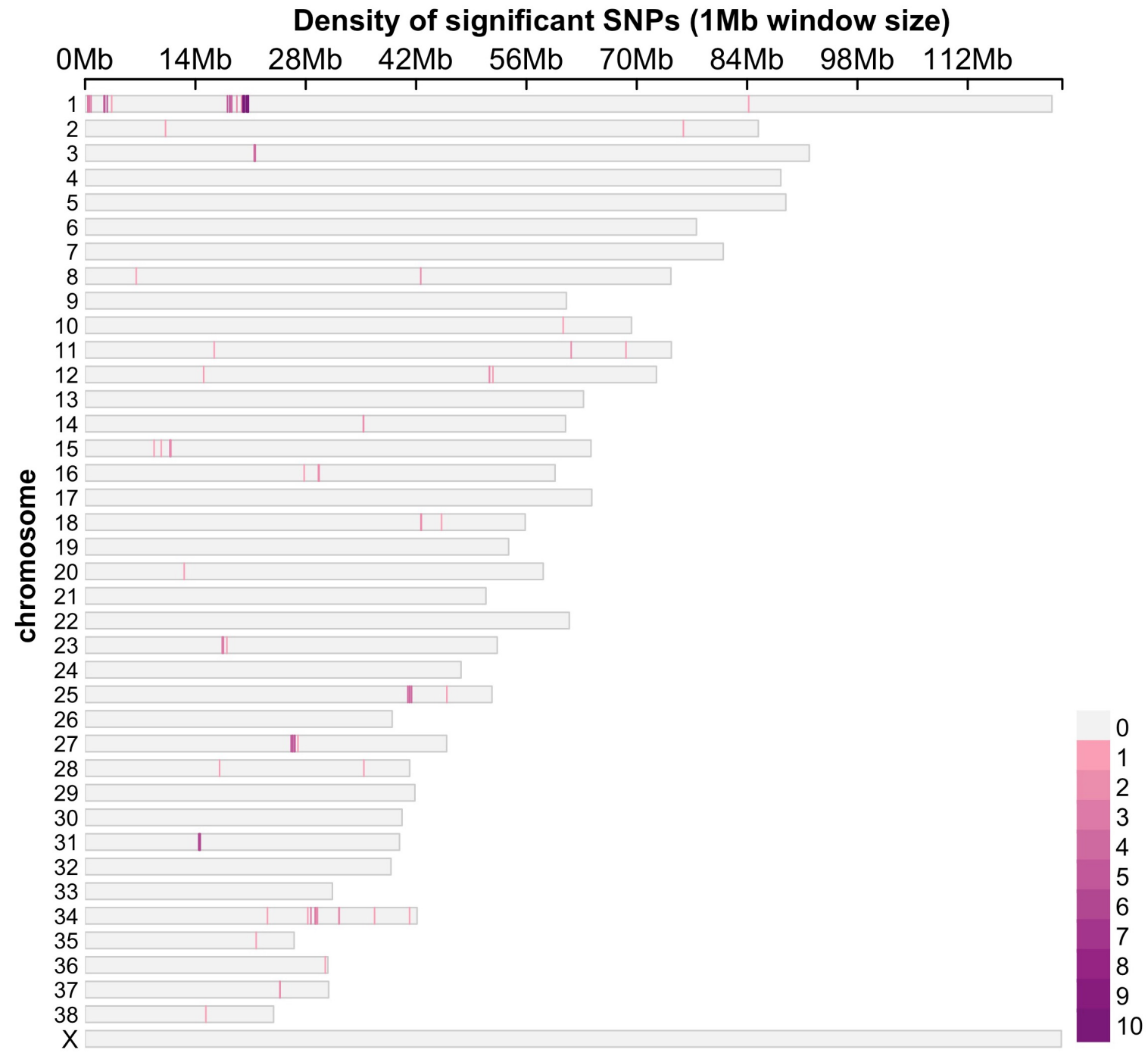

(c)

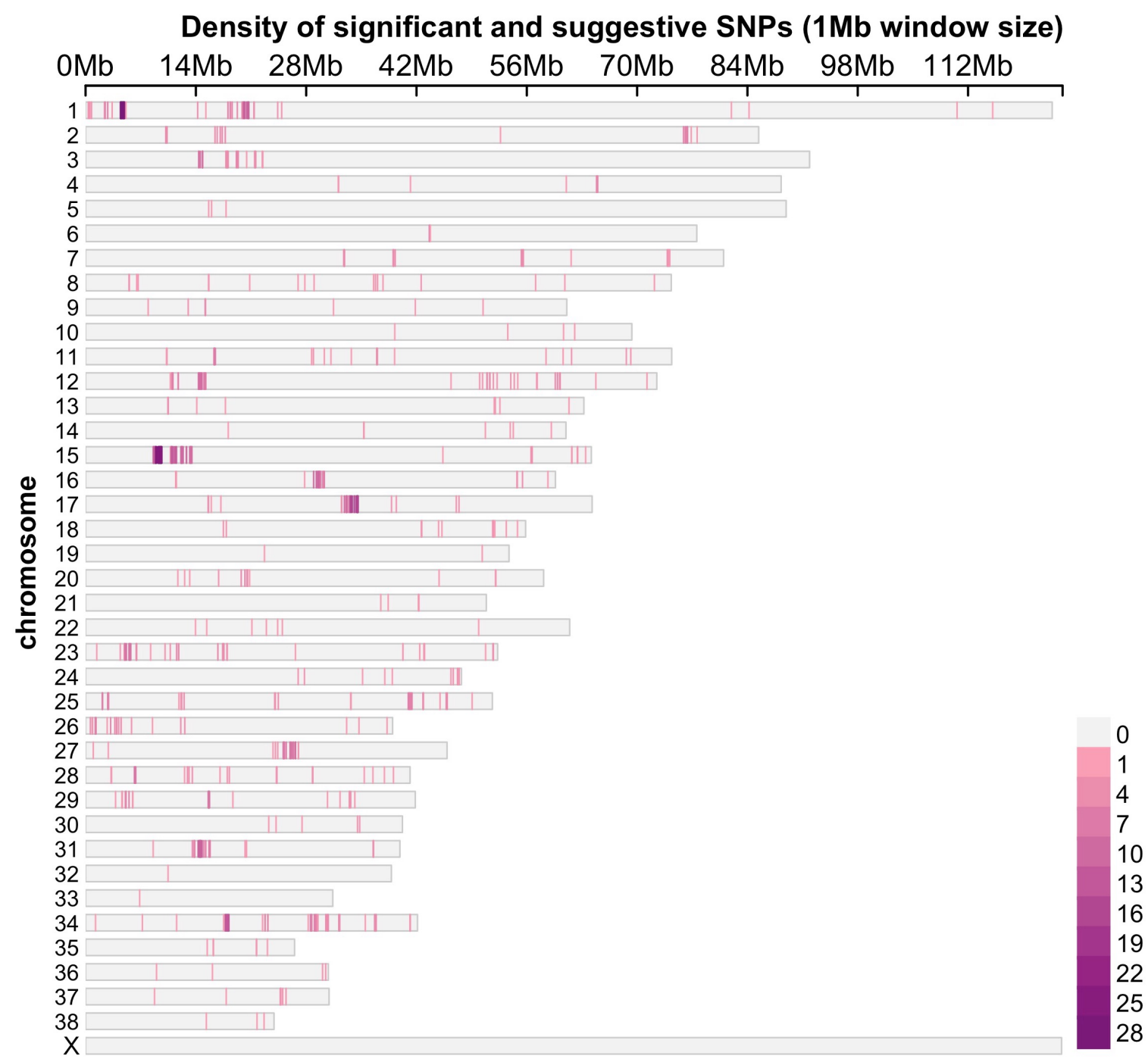

Figure S4. **Genotype-phenotype relationships for SNPs associated with *5d. Tug-of-war*, *DISTANCE PLAYFULNESS* and BOLDNESS.**

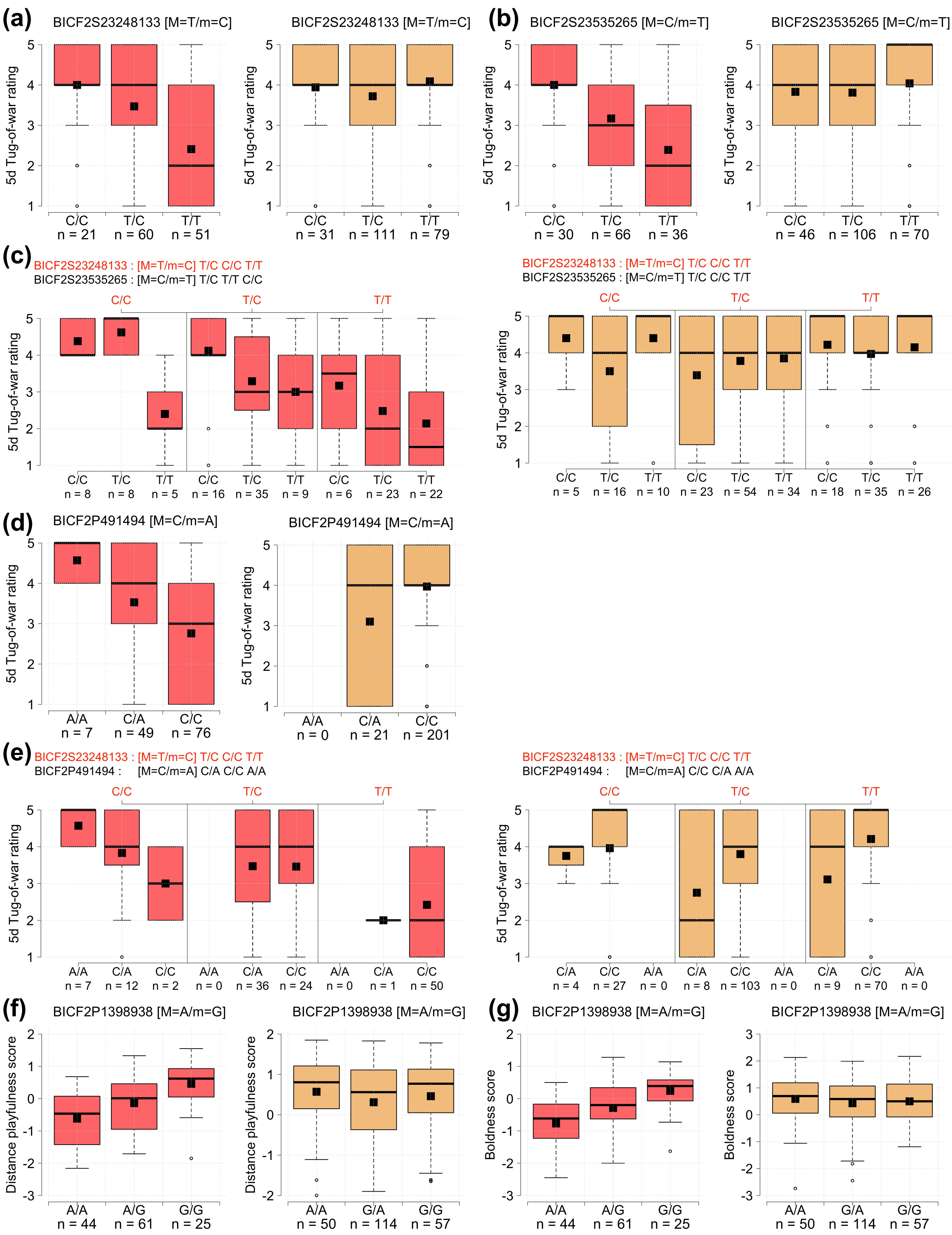

Figure S5. Significant and suggestive association signals for *PLAYFULNESS* and its contributing behavioral variables.

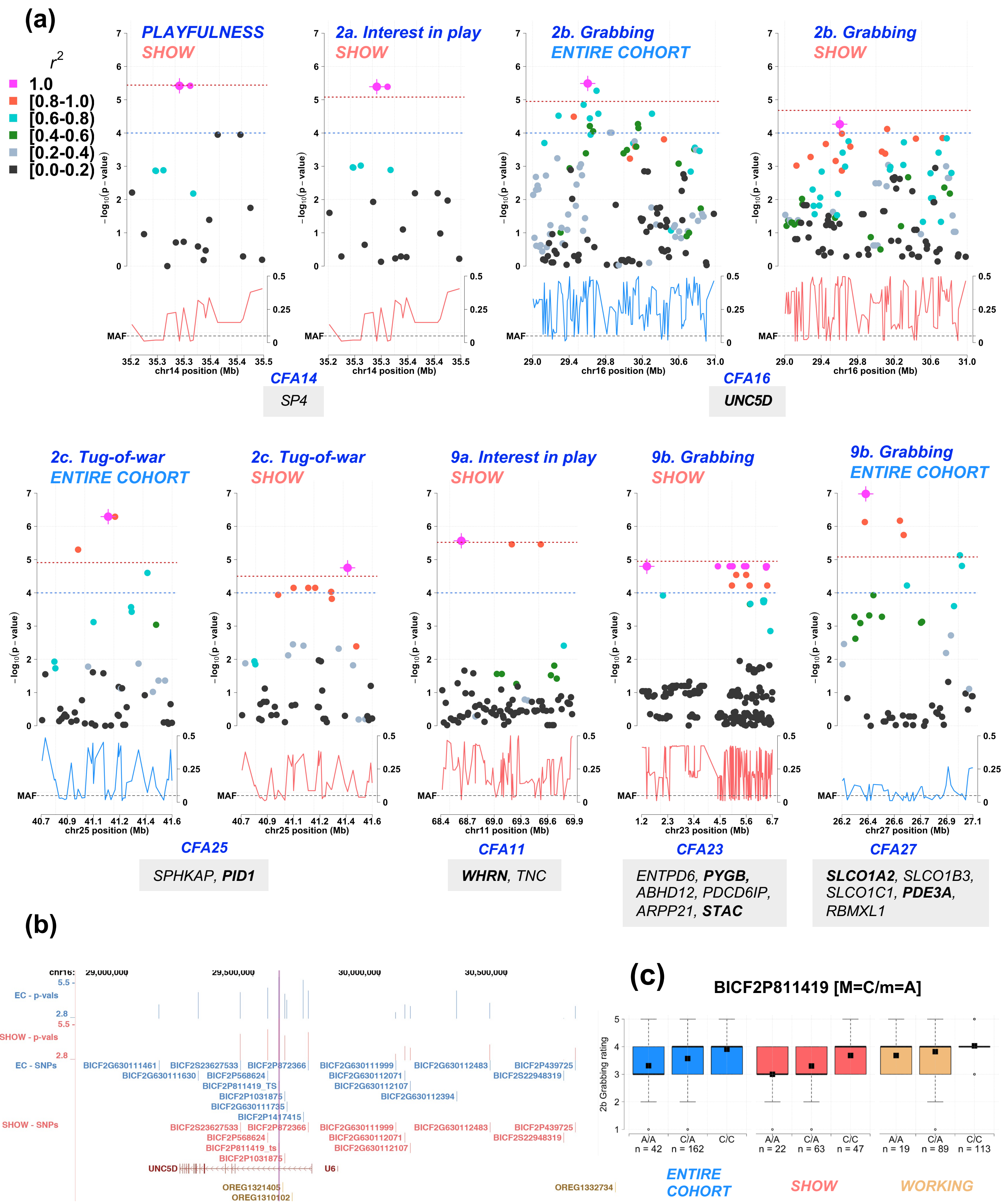

Figure S6. Significant GWAS results in the show sub-population for the *3b. Grabbing 1* behavioral variable contributing to *CHASE-PRONENESS*.

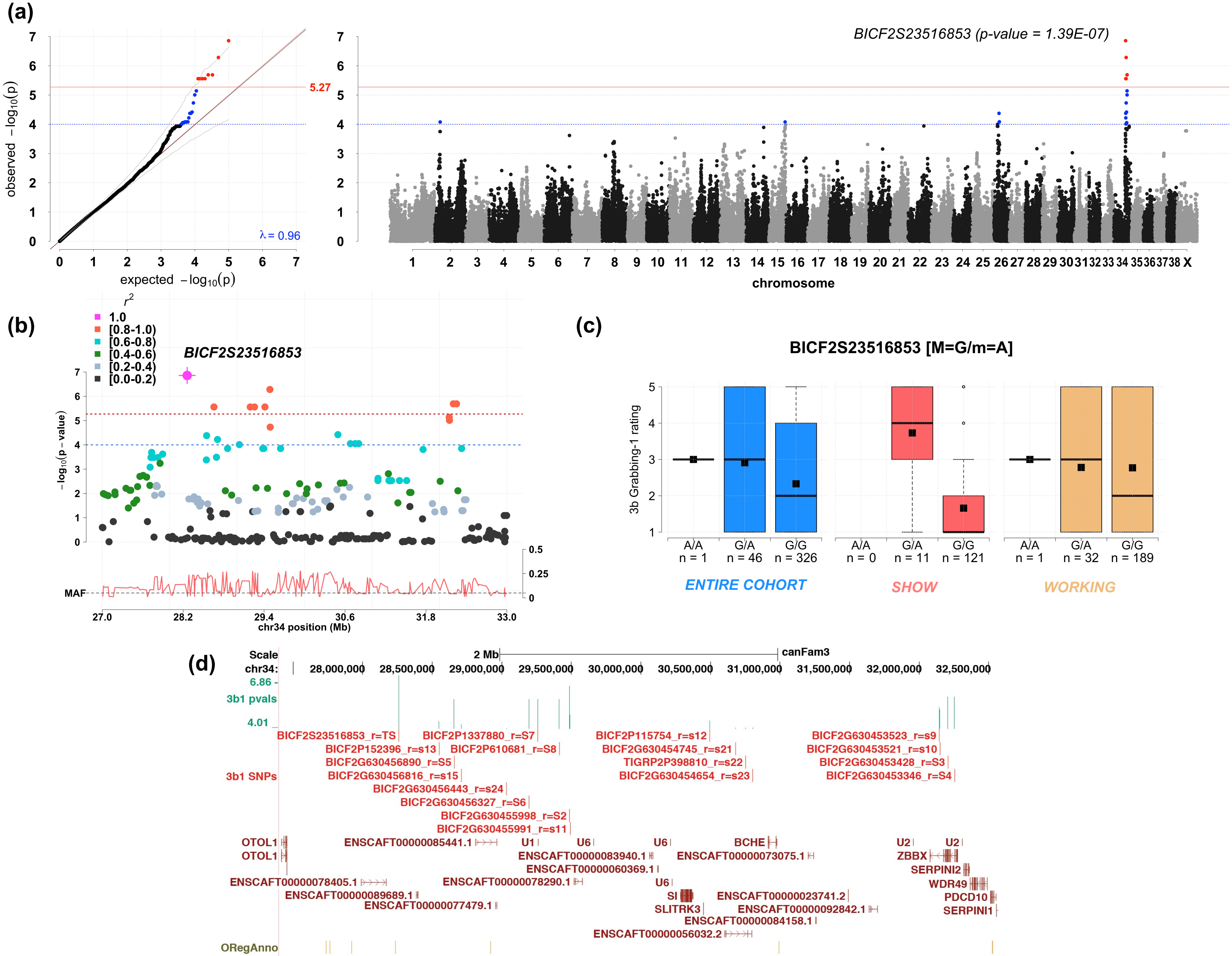

Supplement: Supplementary file 3 [file AGE-57-0-s003.pdf]
