# Supplementary material for: The Genetic Architecture of Behavior in the German Shepherd Dog
Source: Anim Genet. 2026 Nov 10;57(6):e70221. doi: 10.1002/age.70221 (PMC13752456; doi:10.1002/age.70221)
Supplement: Supplementary file 4 [file AGE-57-0-s004.pdf]

SUPPLEMENTARY RESULTS

SR1. Previously reported candidate regions of selection in GSDs, dog domestication and tameness in foxes mapping within the regions of interest in this study

a) *The CFA24:22-26Mb region with highest allele frequency differences between the working and the show sub-populations*

The seventeen most divergent SNPs (-log10$p \geq 20$) and the SNPs in highest LD ($r^2 \geq 0.8$) with the top-divergent SNP occur along the DNA sequence in four distinct groups (Fig. 3F). These groups fell either within or proximal to three regions of recent differential selection between UK and Swedish GSD populations studied (Friedrich, J. *et al*. 2020), *RS1*, *RS2* and *RS3 (*Table S5, Fig. 3F) that were found to be significantly associated with coat color in UK GSDs. A genomic region, *RT1* (Table S5, Fig. 3F), overlapping and extending *RS2* was identified as having nearly complete fixation in tame foxes in a three-way comparison between the genomes of the conventional farm-bred foxes with that of aggressive and tame foxes selectively bred in the long-term Russian farm-fox experiment (Kukekova, A.V. *et al*. 2018). A matching region, *RD1* (Table S5, Fig. 3F), was among those found to undergo recent positive selection in dog domestication (Freedman, A.H. *et al*. 2016).

In addition, a second signal in the allele frequency difference scan found on CFA8 (Fig. 3A) consists of four SNPs scoring -log10$p > 20$ that are intronic to the *protein kinase C eta* (*PRKCH*) gene. These SNPs map close to two regions, *RT2* and *RT3* (Table S5), shown by F_{ST} analyses to have significant differences in allele counts between the genome of tame versus aggressive foxes (Kukekova, A.V. *et al*. 2018); these two regions overlap a region of domestication found in dogs, *RD2* (Wang, G.-d. *et al*. 2013) (Table S5).

b) *The CFA1:20-26Mb region (from the TCF4 to the CCN2 loci) containing signals of association for 5d. Tug-of-war, DISTANCE PLAYFULNESS and BOLDNESS*

This region contains several previously reported regions of selection for dog domestication and regions of recent selection in GSDs, represented as gray bars in Fig. 5A. For simplicity, these are labelled below with *RD* and *RS*, respectively, in the order given in Table S5 (presenting their coordinates and the studies in which they were reported). Firstly, three candidate regions, *RD3*, *RD4*, *RD5* (Fig. 5A, Table S5), were identified as selection signatures of domestication rather than breed formation (Pendleton, A.L. *et al*. 2018). Secondly, a genomic segment, *RD6* (containing the genes *STARD6*, *POLI* and *MBD2*, Fig. 5A), one of 68 regions retrieved by scanning six canid genomes, was found to undergo recent positive selection (Freedman, A.H. *et al*. 2016). Thirdly, a genomic segment *RD7* (47kb upstream of

the suggestive SNP in the *MC2R* promoter), was one of several signals of positive selection during dog domestication identified based on low diversity between seven breeds tested and their high divergence between dogs and wolves (Wang, G.-d. *et al.* 2013). Fourthly, a region of selection, labelled here *RS4*, overlapping the suggestive SNP in the *MC2R* promoter, was identified in a systematic scan for signatures of recent differential selection for morphological and behavioral traits between UK and Swedish GSDs and was found to be associated with chasing in UK GSDs (Friedrich, J. *et al.* 2020).

SR2. Multi-trait SNPs

Twenty SNPs distributed across six chromosomes were retrieved as significant/suggestive for three or more traits/behavioral variables in a set of seventeen that include components of *SOCIABILITY, PLAYFULNESS, DISTANCE PLAYFULNESS, CHASE-PRONENESS* and BOLDNESS. We refer to these SNPs as multi-trait significant/suggestive SNPs. Table S7A shows the traits/behavioral variables for which they were retrieved and the GWAS runs in which they were identified. Additional details on their chromosomal location, significance/suggestive rank in the GWAS (by *p* value) and the GWAS runs in which they were retrieved are provided in Table S7B. Of particular importance, a cluster of predominantly significant SNPs was found within a ~3Mb region on CFA1 for *5d. Tug-of-war*, *DISTANCE PLAYFULNESS*, and BOLDNESS. Multi-trait SNPs were also retrieved for 1) *DISTANCE PLAYFULNESS* and its component *5c. Exploration* on CFA8; 2) for *PLAYFULNESS* and its components *2b. Grabbing* and *9a. Interest in play* on CFA26; 3) for *SOCIABILITY* and its components *1a. Greeting reaction* and *1b. Cooperation* on CFA18; and 4) for *CHASE-PRONENESS* and its components *3a. Following 2*, *3b. Grabbing 1* and *3b. Grabbing 2* on CFA11 and CFA16.
Retrieving the same SNPs as significant/suggestive for both the traits and the behavioral variables that load on them is reassuring, however, it is likely due to the high loadings of the corresponding behavioral variables on the trait. Nevertheless, this highlights the complex phenotypic relationships between the DMA traits/behavioral variables reflected in many cases in the high correlation between their values and could also point to a common architecture that their genetic determinants may share.

SR3. Genotype-phenotype relationships for the top SNPs associated with BOLDNESS, DISTANCE PLAYFULNESS and 5d. Tug-of-war

The distribution of phenotypic scores for different genotype combinations for selected SNPs associated with these traits on CFA1 and CFA2 are presented in Fig. S4. For the top SNP for *5d* on

CFA1 (BICF2S23248133), the minor allele C confers higher phenotypic scores in the *show* sub-population but has little effect in the *working* sub-population (Fig. S4A). For the significant SNP for *5d* on CFA2 (BICF2S23535265), the minor allele T confers lower phenotypic scores in *show* dogs but has little effect in *working* dogs (Fig. S4B). However, when the effects of all possible genotype combinations for these two SNPs are broken down in a by-way analysis (Fig. S4C), it becomes apparent that the minor allele T of BICF2S23535265 on CFA2 lowers the phenotypic score in the *show* sub-population even if the dogs carry the minor allele C of BICF2S23248133 on CFA1 (that otherwise would confer higher phenotypic scores). These suggests a possible interaction between variation at the *TCF4* and *E2F2* gene loci affecting the *5d* phenotypic scores in the *show* sub-population.

The minor allele A of the suggestive SNP in the promoter of *MC2R* (BICF2P491494) confers in the *show* sub-population, higher *5d* phenotypic scores and is present as homozygous in only seven *show* dogs (Fig. S4D), all of which are homozygous for the minor allele C of the *5d* top SNP BICF2S23248133 as shown in by-way analyses (Fig. S4E). In the *working* sub-population, dogs homozygous for the minor allele C of the top SNP BICF2S23248133 that also carry the minor allele A of BICF2P491494 have lower *5d* scores, as shown in the by-way analyses (Fig. S4E). This also points towards a possible interaction between variation at the *TCF4* and *MC2R* loci with respect to the *5d* phenotypic scores.

In the *show* sub-population, the minor allele G of BICF2P1398938 (the top SNP for *DISTANCE PLAYFULNESS* and BOLDNESS), confers higher phenotypic scores for these traits; *show* dogs homozygous for the allele have comparable or slightly higher scores than *working* dogs that display approximately the same score values for all genotype combinations (Fig. S4F and S4G).

SR4. GWAS results for additional traits/behavioral variables most positively related to BOLDNESS

PLAYFULNESS and its contributing behavioral variables

We found numerous significant and suggestive SNPs for *PLAYFULNESS* and its contributing behavioral variables in the three GWAS runs (Table S8). For the strongest signals by *p*-value, the top SNPs and the SNPs in LD with them are presented in Fig. S5A. In the *show* sub-population, SNPs retrieved as suggestive for *PLAYFULNESS* and significant for *2a. Interest in play,* point to a region on CFA14 upstream of the *Sp4 transcription factor* (*SP4*) gene.

For *2b. Grabbing*, SNPs significant in the entire cohort and suggestive in the *show* sub-population map to the *unc-5 netrin receptor D* (*UNC5D*) locus on CFA16. The top SNP for *2b* in the entire cohort (BICF2P811419) is in moderately high LD (r^2>=0.7) with eight other SNPs

within a region of ~1.5Mb (Fig. S5B); five of these SNPs, including the top SNP are intronic to *UNC5D* while the remaining three map immediately upstream of the gene. The minor allele of the top SNP is associated with a decrease in *2b* score in the entire cohort and in the *show* sub-population (Fig. S5C).

For *2c. Tug-of-war,* significant SNPs in the entire cohort and in the *show* sub-population map to a region of CFA25 containing the *SPHK1 interactor, AKAP domain containing* (*SPHKAP*) and the *phosphotyrosine interaction domain containing 1* (*PID1*) genes (Fig. S5A). A significant SNP for *9a. Interest in play* in the *show* sub-population maps to *whirlin* (*WHRN*) on CFA11; the region also contains *tenascin C* (*TNC*) harboring an additional suggestive SNP.

For *9b. Grabbing*, an association signal was detected in the entire cohort on CFA27 with the significant SNPs mapping to the *solute carrier organic anion transporter family member 1A2* (*SLCO1A2*) and *phosphodiesterase 3A* (*PDE3A*) genes.

For *PLAYFULNESS*, *2b* and/or *9a* three suggestive multi-trait SNPs were retrieved as presented in Supplementary Results section SR1 and Tables S7A and S7B.

3b1. Grabbing 1 contributing to *CHASE-PRONENESS*

A significant association signal was obtained on CFA34 in the *show* sub-population for *3b1. Grabbing 1* (Fig. S6A). Within a span of ~3.5 Mb, the top SNP (BICF2S23516853, -log10p = 6.9) is in high LD ($r^2 \geq 0.8$) with 10 other significant and suggestive SNPs (Fig. S6B). Homozygotes for the major allele G of BICF2S23516853 constitute the majority in the *show* and *working* sub-populations (~92% and ~85%, respectively). Compared to heterozygotes, their scores are notably lower in the *show* sub-population and have a lower median in the *working* sub-population. The significant and suggestive SNPs are distributed in two clusters on CFA34, one located in the intragenic region between *otolin 1* (*OTOL1*) and *sucrase isomaltase* (*SI*) (Fig. S6D), the other containing SNPs intronic to the *Zinc finger B-box domain containing* (*ZBBX*) gene.

SOCIABILITY and its contributing behavioral variables

In the *show* sub-population, multiple significant signals for *SOCIABILITY* and its components *1a. Greeting reaction* and *1b. Cooperation* were found within the CFA18:42.6-43.5Mb region (Table S8). These include a multi-trait SNP (BICF2P245560, Table S7B) representing the top SNP for both *SOCIABILITY* and *1a* that is also the highest ranking suggestive SNP for *1b*, mapping near the *low density lipoprotein receptor-related protein 4* (*LRP4*) and *cytoskeleton associated protein 5* (*CKAP5*) genes. A significant SNP for both *SOCIABILITY* and *1a* (BICF2P517207) is located upstream of BICF2P245560, in the intragenic region between *C18H11orf49* and *LRP4*.

SR5. Additional significant/suggestive SNPs mapping to or close to genes for which an association with neuroticism in humans has also been reported

Several other significant/suggestive SNPs retrieved in different GWAS runs for BOLDNESS and BOLDNESS-related traits/behavioral variables map to or in the proximity of genes that have previously been associated with neuroticism in humans (Table S8). These include: 1) suggestive signals for BOLDNESS on CFA16 (mapping to *CSMD1*), 2) significant signals for *2a. Interest in play* on CFA14 (upstream of *SP4*), *2b. Grabbing* on CFA16 (mapping to *UNC5D*) and CFA28 (mapping to *SORCS3*), *1a. Greeting reaction* and *SOCIABILITY* on CFA18 (upstream of *LRP4*), and 3) suggestive signals for *PLAYFULNESS* on CFA14 (upstream of *SP4*), *9b. Grabbing* on CFA23 (upstream of *ARPP21*) and *6d. Remaining avoidance behavior* on CFA15 (mapping to *AGBL4*).

SR6. Enrichment analyses for the identified candidate genes presented in Table S8

Enrichment analysis (Table S9) for the set of 54 unique genes overlapping or located immediately upstream and downstream of the significant and suggested markers in Table S8 performed with *Enrichr* (Kuleshov, M.V. *et al*. 2016) against knowledge databases showed the following top significant results (for complete results see Table S9): 1) For KEGG pathways: *Cushing's syndrome* ($p = 7.9\times10^{-03}$, overlap 3 genes-in-set /151 genes-in-pathway, genes involved *MC2R*, *E2F2, WNT4*), *glutathione metabolism* ($p = 1.1\times10^{-02}$, 2/59, *GPX7*, *TXNDC12*) and *insulin resistance* ($p = 3.4\times10^{-02}$, 2/107, *PYGB*, *PPP1R3C*), 2) for Gene Ontology (GO) Biological Process: *positive regulation of steroid hormone biosynthetic process* (GO:0090031, $p = 7.12\times10^{-05}$, 2/5, *MC2R*, *WNT4*); for GO Molecular Function: *neuropeptide receptor activity* (GO:0008188, $p = 7.45\times10^{-03}$, 2/48, *MC2R*, *SORCS3*); and for GO Cellular Compartment: *synaptic membrane* (GO:0097060, $p = 6.29\times10^{-03}$, 2/44, *SLITRK3*, *LRP4*).

Enrichment analyses performed against the ARCHS4 Tissues database containing the 2316 most highly or specifically expressed genes for every tissue profile in human and mouse (Lachmann, A. *et al*. 2018) indicated that a total of 25 out of the 54 candidate genes were highly expressed in the *prefrontal cortex* (PFC) and the *spinal cord* (SC) ($p = 2.07\times10^{-05}$, 18/2316) as follows: 1) candidate genes highly expressed in both PFC and SC: *ARPP21, BCHE, CSMD1, CYP4X1, ELAVL4, LRP4, SLITRK3, SORCS3, SPHKAP, UNC5D, ZBBX*; 2) additional candidate genes highly expressed in the PFC: *AGBL4, MC2R, PID1, SLCO1A2, TCF4, TNC, ZYG11B*; and 3) additional candidate genes highly expressed in the spinal cord: *ARMC3, OTOL1, PDE3A, TRABD2B, WHRN, WNT4, ZFYVE9*.

For the DisGeNET database of human gene-disease associations (Pinero, J. *et al*. 2020) four genes (*KDM1A, SP4, TCF4, CSMD1*) were found to be statistically overrepresented in *other specified types of schizophrenia* ($p = 1.26\times10^{-05}$, 4/53) and *schizophrenia 1 disorder* ($p = 1.81\times10^{-05}$, 4/58).

References

Freedman, A.H., Schweizer, R.M., Ortega-Del Vecchyo, D., Han, E., Davis, B.W., Gronau, I., . . . Novembre, J. (2016). Demographically-based evaluation of genomic regions under selection in domestic dogs. *PLoS Genet* **12**(3): e1005851. *10.1371/journal.pgen.1005851*.

Friedrich, J., Talenti, A., Arvelius, P., Strandberg, E., Haskell, M.J. and Wiener, P. (2020). Unravelling selection signatures in a single dog breed suggests recent selection for morphological and behavioral traits. *Adv Genet (Hoboken)* **1**(1): e10024. *10.1002/ggn2.10024*.

Kukekova, A.V., Johnson, J.L., Xiang, X., Feng, S., Liu, S., Rando, H.M., . . . Zhang, G. (2018). Red fox genome assembly identifies genomic regions associated with tame and aggressive behaviours. *Nat Ecol Evol* **2**(9): 1479-1491. *10.1038/s41559-018-0611-6*.

Kuleshov, M.V., Jones, M.R., Rouillard, A.D., Fernandez, N.F., Duan, Q., Wang, Z., . . . Ma'ayan, A. (2016). Enrichr: A comprehensive gene set enrichment analysis web server 2016 update. *Nucleic Acids Res* **44**(W1): W90-97. *10.1093/nar/gkw377*.

Lachmann, A., Torre, D., Keenan, A.B., Jagodnik, K.M., Lee, H.J., Wang, L., . . . Ma'ayan, A. (2018). Massive mining of publicly available rna-seq data from human and mouse. *Nat Commun* **9**(1): 1366. *10.1038/s41467-018-03751-6*.

Pendleton, A.L., Shen, F., Taravella, A.M., Emery, S., Veeramah, K.R., Boyko, A.R. and Kidd, J.M. (2018). Comparison of village dog and wolf genomes highlights the role of the neural crest in dog domestication. *BMC Biol* **16**(1): 64. *10.1186/s12915-018-0535-2*.

Pinero, J., Ramirez-Anguita, J.M., Sauch-Pitarch, J., Ronzano, F., Centeno, E., Sanz, F. and Furlong, L.I. (2020). The disgenet knowledge platform for disease genomics: 2019 update. *Nucleic Acids Res* **48**(D1): D845-D855. *10.1093/nar/gkz1021*.

Wang, G.-d., Zhai, W., Yang, H.-c., Fan, R.-x., Cao, X., Zhong, L., . . . Zhang, Y.-p. (2013). The genomics of selection in dogs and the parallel evolution between dogs and humans. *Nature Communications* **4**(1): 1860. *10.1038/ncomms2814*.
